# Supplementary material for: Clinicopathological-Associated Regulatory Network of Deregulated circRNAs in Hepatocellular Carcinoma
Source: Cancers (Basel). 2021 Jun 2;13(11):2772. doi: 10.3390/cancers13112772 (PMC8199648; doi:10.3390/cancers13112772)
Supplement: Supplementary file 1 [file cancers-13-02772-s001.zip › cancers-1214052-supplementary.pdf]

SUPPLEMENTAL INFORMATION

Clinicopathological-associated regulatory network of deregulated circRNAs in hepatocellular carcinoma

Jian Han[1], Thomas Thurnherr[3], Alexander Y.F. Chung[4], Brian K.P. Goh[4], Pierce K.H. Chow[4,5,6] , Chung Yip Chan[4], Peng Chung Cheow[4], Ser Yee Lee[4], Tony K.H. Lim[7], Samuel S. Chong[8], London L.P.J.Ooi[4,5,6]Caroline G. Lee [1,2,3,5*]

*Corresponding author's email: bchleec@nus.edu.sg

This file includes:

SUPPLEMENTARY MATERIALS AND METHODS 3
Figure S1 16
Figure S2 16
Figure S3 17
Figure S4 18
Figure S5 19
Figure S6 20
Figure S7 21
Figure S8 22
Figure S9 23
Figure S10 24
Figure S11 24
Table S1 25
Table S2 26
Table S3 27
Table S4 30
Table S5 33
Table S6 39
Table S7 40
Table S8 55

SUPPLEMENTARY MATERIALS AND METHODS

circRNA, miRNA AND mRNA EXPRESSION PROFILING

Frozen tissue samples were homogenized by gentleMACS dissociator (Miltenyi Biotec, Germany) in RLT buffer (Qiagen, Germany) with 1% β-mercaptoethanol. Total RNA was extracted from tissues samples using RNeasy mini Kit (Qiagen, Germany) following the manufacturer's protocol and quantified using NanoDrop ND-1000 spectrometer (Nanodrop Products, USA).

Total RNA from each sample was quantified, with the NanoDrop ND-1000, and RNA integrity was assessed using the Agilent 2100 Bioanalyzer. All RNA samples showed OD A260/280 ratio between 1.8 and 2.1 and an OD A260/230 ratio >1.8. RNA integrity (RIN) score were above 7. For circRNA's microarray, the sample preparation and microarray hybridization were performed based on the Arraystar's standard protocols. Briefly, total RNAs were digested with RNase R (Epicentre Inc, USA) to remove linear RNAs and enrich circular RNAs. Then, the enriched circular RNAs were amplified and transcribed into fluorescent cRNA utilizing a random priming method-Arraystar Super RNA Labeling Kit (Rockville, MD, USA). The labeled cRNAs were hybridized onto the Arraystar Human circRNA Array V2 (8x15K, Arraystar). After quantile normalization of the raw data, low intensity filtering was performed. circRNA that have flags in Present or Marginal in at least 50% samples were used for data analysis. miRNA sequencing was performed on Illumina NextSeq 500 using TruSeq Rapid SBS Kits (#FC-402-4002, Illumina). For miRNA alignment, the maximum mismatch was 1. Reads with counts less than 2 were discarded when calculating the miRNA expression. For mRNA microarray analysis, the labeled cRNAs were hybridized onto the Human LncRNA Array v4.0 (8 x 60K, Arraystar). mRNA that have flags in Present or Marginal in at least 50% samples were used for data analysis.

DEREGULATED circRNAs, miRNAs AND mRNAs

Paired student's t-test was performed to determine the differentially expressed circRNAs, miRNAs and mRNAs between tumorous and adjacent non-tumorous tissues of 49 HCC patients. To account for multiple testing, we employed the Benjamini-Hochberg (BH) method to calculate false discovery rate (FDR) values. miRNAs and circRNAs with $|FC|>1.5$ and $FDR<0.05$ were considered significantly differentially expressed. And mRNA with an $|FC|>2$ and $FDR<0.05$ were considered significantly differentially expressed. The R package "heatmap3" was employed to do the unsupervised hierarchical clustering analysis.

ASSOCIATED CLINICAL FEATURES OF DEREGULATED circRNAs, miRNAs AND mRNAs

To identify clinically relevant deregulated circRNAs, miRNAs and mRNAs, tumor samples were divided into two groups for each clinical phenotype (Supporting Table S1). For each clinical phenotype, we compared the expression of circRNAs, miRNAs and mRNAs in the tumor of the group with unfavourable clinical characteristics versus the tumors of patients with favourable clinical characteristics (e.g. patients with high Edmondson grade vs. low Edmondson grade), and identified those with $P<0.05$, $|FC|>1.5$ using a t-test. For overall survival analyses, the 49 patients were divided into two groups using the median survival time as a cut-off. The Kaplan–Meier plot was used to illustrate the difference in overall survival time between the two groups. The significance was assessed using the log-rank test. circRNAs, miRNAs and mRNAs that showed a significant increase or decrease in expression in both tumor compared to non-tumor and unfavourable clinical characteristics compared to favourable clinical characteristics in tumor tissues were grouped as potentially worse prognostic RNAs or better prognostic RNAs, respectively.

CLINICALLY RELEVANT, DEREGULATED, CO-EXPRESSED circRNA-mRNA NETWORKS

For potentially worse and better prognostic circRNAs, the associated mRNAs were evaluated via "Guilt-by-association" principle [30,31]. circRNA-mRNA pairs with a Pearson correlation coefficient $|r|>0.6$ and $FDR<0.05$ were considered strong correlated [26,58]. Co-expression networks for clinically relevant co-expressed circRNAs-mRNAs across tumor and adjacent non-tumor tissues were built by Cytoscape [27]. The biological function of the clinically relevant circRNA-mRNA network was predicted using ConsensusPathDB [28,59]. The enriched pathways with a value of $P<0.01$ are considered significant.

COMPUTATIONAL PREDICTION OF CLINICALLY RELEVANT circRNA-miRNA-mRNA ceRNA NETWORK

Differentially expressed miRNAs, mRNAs and circRNAs between tumor and normal were identified based on $FDR<0.05$ and $|FC|>1.5$ for miRNAs and circRNAs, $|FC|>2$ for genes. BH method was used to correct the P value for multiple comparisons.

Then "Association" and "Prediction" were used to identify the potential circRNAs and genes targeted by miRNA. "Association" is analysed using Spearman's rank correlation. "Prediction" is performed using two miRNA target prediction tools: miRanda and PITA. miRanda predicts mainly based on seed match, free energy and conservation between miRNA and its targets [22,60], while PITA predicts mainly based on target site accessibility [23,24].

Once the miRNA-mRNA, and miRNA-circRNA pairs were identified, hypergeometric test [25] was employed to calculate the significance of sharing of miRNAs by the two ceRNAs, to preliminary identify all the significant ceRNA pairs. Thereafter, additional 4 statistical methods (Pearson positive correlation (PC) [25,61], Partial Pearson correlation (PPC)[62], sensitive partial Pearson correlation (SPPC) [62], and conditional mutual information (CMI) [63]) were employed to identify highly confident ceRNAs that are significant based on all 4 statistical tests. Since 2 ceRNAs competing for the same miRNAs should exhibit similar expression direction tendency [26,64], only positively correlated pairs with $p<0.05$ based on Pearson Correlation (PC) are considered as reliable ceRNA pairs [25]. Partial Pearson correlation (PPC) was employed as it measures the extent to which an observed correlation between two variables X and Y (i.e. the expression profiles of a mRNA and a circRNA) relies on the presence of a third controlling variable Z (here, the expression profile of a miRNA) [62]. Hence, miRNA sponge interaction pairs with statistical significance ($p<0.05$) are considered as significant miRNA sponge interaction pairs. Sensitive Partial Pearson Correlation (SPPC) measures the sensitivity (S) of the miRNA in the circRNA-miRNA-mRNA triplet and is the difference between PC and PPC [62]. The miRNA sponge interaction-pairs with $S > 0.3$ are regarded as significant miRNA sponge interaction pairs. Conditional Mutual Information (CMI) estimate how one miRNA sponge affect its competing miRNA sponges. It measures the statistical significance of the difference between the CMI of conditional expression of miRNA and its target RNA (e.g. circRNA/mRNA), and the (unconditional) mutual information of the miRNA and target expression levels. Significant miRNA sponge pairs are those with CMI statistical significance of $p<0.05$ [63].

ceRNA pairs (namely, circRNA-circRNA, circRNA-mRNA and mRNA-mRNA) which are statistically significant ($p<0.05$) for all 4 statistical (PC, PPC, SPPC and CMI) tests are considered as the highly confident ceRNA pairs while ceRNA pairs which exhibit statistical significance for 2 of the 4 statistical tests (ie. PC and PPC), are regarded as moderately confident ceRNA pairs.

CELL CULTURE

The immortalized liver cells LO2 was a gift to our lab by Professor Guan Xin Yuan, Director of Laboratory of Cancer Genetics, Hong Kong University. It was authenticated before using. The human hepatocellular carcinoma transformed cell Huh7 was purchased from Thermofisher. HCC cell lines HepG2 and SNU449, and the human embryonic kidney cell HEK293T were purchased from ATCC. All the cells were grown in Dulbecco's Modified Eagle's medium (St.louis MO, USA) with the addition of 10% Fetal Bovine Serum (FBS) (Biological Industries, Isreal). The cells were grown in a humidified 37 °C incubator with 5% CO2.

TOTAL RNA EXTRACTION FROM CELLS AND REVERSE TRANSCRIPTION QUANTITATIVE REAL-TIME PCR ANALYSIS

Total RNA was extracted using RNeasy mini Kit (Qiagen, Germany) following the manufacturer's protocol. Total RNA for miRNA detection was extracted using mirVana™ miRNA Isolation Kit (Invitrogen, USA) following the manufacturer's protocol.

The cDNAs of circRNAs and mRNAs were synthesized from 1.5 µg of total RNA with the SuperScript™ II Reverse Transcriptase (Invitrogen, USA) and random primers (Invitrogen, USA). Before qRT-PCR is performed, primer specificity is ensured through observing a single clear band on agarose gel as well as single peak in a melting curve analyses. Real-time was performed using SYBRTM Green PCR Master Mix (Life Technologies, UK) on 7500 Real-Time PCR System (Applied Biosystem, USA). The differential expression

of mature miRNAs was validated using Taqman MicroRNA Individual Assays (Applied Biosystems, USA). Reverse transcription reaction was carried out on 75 ng of template total RNA using the High Capacity cDNA Archive Kit (Applied Biosystems, USA) and miRNA specific reverse transcription primers. Real-time PCR was performed on 7500 Real-Time PCR System (Applied Biosystem, USA) using Taqman 2X Universal PCR Master Mix without UNG Amperase and miRNA specific primers. The expression levels were normalized to GAPDH (for circRNAs and mRNAs) or RNU48 (for miRNAs). Expression was measured by threshold cycle (CT), which was determined as the fractional cycle number at which the fluorescence intensity exceeded a fixed threshold, and the ΔΔCT method was employed to calculate relative expression [22]. All the primers for RNAs, siRNAs for circRNAs and miRNAs, and probes for circRNAs were listed in Supporting Table S2.

RNASE R TREATMENT

For RNase R treatment, 1.5 μg total RNA was incubated for 30 minutes at 37°C with or without 3 U/mg RNase R (Epicenter Technologies, Madison, WI, USA). After removal of RNASE R, the expression levels of circRNAs were determined by reverse transcription quantitative PCR (RT-qPCR).

CELL TRANSFECTION

LO2, Huh7, HepG2 or SNU449 cells were seeded 500,000 to 6 wells. When cells reached ~70% confluency, Lipofectamine™ RNAiMAX Transfection Reagent (Invitrogen, USA) was then employed to introduce siRNAs and small oligos, such as control Oligos, miRNA mimics and miRNA inhibitors. Lipofectamine™ 3000 Transfection Reagent (Invitrogen, USA) was used to introduce plasmid constructs. The siRNAs, miRNA mimics and miRNA inhibitors for these studies as well as primers for amplifying the various

genes/circRNAs are shown in Supporting Table S2.

GENETIC ENGINEERING OF circRNA PLASMID CONSTRUCTS AND GENERATION OF STABLE CELL LINES

To construct circGPC3 plasmid, the linear transcript of circGPC3 (including upstream 835bp and downstream 525bp) was amplified from cDNA of HepG2 cell lines using primers as shown in Supporting Table S2 and Expand™ High Fidelity PCR System (Roache, USA). The full-length 2055bp fragment was cloned into vector pCDH-CMV-MCS-EF1-GFP+Puro via T4 ligation (Thermo Scientific, USA).

For circW3, circW7 and circW8, the linear parts of circRNAs were amplified from cDNA of Huh7 using primers as shown in Supporting Table S2 and Expand™ High Fidelity PCR System (Roache, USA), which include the AG accept, GT donor, and the front/back circular frame. The circRaly was amplified from the gDNA of HepG2. The linear circRNA fragments were inserted into vector pCD-ciR (Guangzhou, China).

FLUORESCENCE IN SITU HYBRIDIZATION (FISH) AND CELL FRACTION

20,000 HepG2 cells were seeded into 24 wells. The FISH probe was designed across the junction site of circGPC3 as listed in Supporting Table S2. The hybridization was performed overnight with the circGPC3 FISH probes (5'-AAAGGCCTAGTGGTGGTCAGCT-3'biotin). Nuclei were stained using Hoechst 33342 (2'-[4-ethoxyphenyl]-5-[4-methyl-1-piperazinyl]-2,5'-bi-1H-benzimidazole trihydrochloride trihydrate) (Thermofisher, USA). All the procedures were conducted according to the manufacturer's instruction (Genepharma, Shanghai, China). All images were acquired on Zeiss microscope system (Leica Microsystems, Mannheim, Germany).

Nuclear and cytoplasmic fractions of cells were separated using a PARIS™ Kit (Life Technologies, USA) according to the manufacturer's protocol. Expression of circRNAs were assessed in cytoplasmic and nuclear fraction using real-time PCR. lncMalat1 and β-actin were used as control to indicate the successful extraction of nuclear and cytoplasmic RNAs respectively.

CELL PROLIFERATION ASSAY

Treated cells were seeded 100,000 to 24-wells and placed into the automated real-time cell imaging (ACRI) system of IncuCyte™ ZOOM (Essen BioScience, USA), which measures the relative cell confluency at every 6 hours interval over 5~6 days. The cell doubling time during the exponential growth phase was calculated using this formula: Doubling Time = duration ∗ log(2)/ [log(FinalConcentration)−log(InitalConcentration)][65].

CELL CYCLE PROFILE

Cells were seeded in 6 wells and transfected with siRNA or plasmids. Cells were harvested using trypsin at 24h, 48h and 72h, washed in phosphate-buffered saline (PBS), and resuspended in 70% ethanol for storage at -20˚C overnight. Cells were stained with propidium iodide 10 g/ml (Invitrogen, USA) for 20 min in the dark at room temperature and their DNA content was analyzed with a flow cytometry (BD Biosciences, San Jose, CA, USA). The ratios of cells in the G0/G1, S, G2 phases were counted and compared.

ANCHORAGE INDEPENDENT GROWTH

100,000 LO2, Huh7 and 50,000 HepG2 cells transfected with various siRNAs/plasmids were mixed with 0.6% low melting point agar (Thermo Scientific, USA) and then they were introduced on top of a solid 0.8% agar layer in 6 wells. Cell culture media was then added when

the agar layer solidified and the cells were then placed in an incubator at 37’C, 5% CO2 for 28 days (LO2 and Huh7) or 14 days (HepG2) with media change every 3 days. The colonies of cells were stained with methyl green at the end of the assay, taken photos and counted manually.

WOUND HEALING ASSAY

50,000 LO2, Huh7, SNU449 and 100,000 HepG2 cells were added into wells of a 96-well ImageLock^{TM} plate and left to attach overnight. A scratch wound was made using the WoundmakerTM 96 (Essen BioScience, USA). The 96-well plate was placed into an ACRI system for each well were taken at 3 hours’ intervals, up to 96 hours was collected and analyzed. The measurement parameter used was relative wound density. The relative wound density is self-normalizing and corrects for changes in cell density due to other confounding effects like cell proliferation and/or pharmacological effects. It is a more reliable metric than the conventional wound width measurement [24].

TRANSWELL INVASION ASSAYS

The LO2 and SNU449 cells (30,000 cells/well), Huh7 and HepG2 cells (50,000 cells/well) were added to the upper chamber with 500 µl of DMEM without fetal bovine serum, and 1000 µl of DMEM medium with 10% FBS was added to the lower chamber. The chambers were separated by a porous membrane. After 72 h of incubation, the cells in the lower chamber were fixed with 3.7% formaldehyde and 100% methanol. After washing with PBS twice, the cells were stained with KaryoMAX™ Giemsa Stain Solution (Invitrogen, USA) and de-stained with Gurr buffer. The number of penetrated cells was assessed using a microscope at 200x magnification and analyzing three random fields.

LUCIFERASE REPORTER ASSAY

Wild-type circGPC3 (circGPC3 WT), mutant circGPC3 (circGPC3 Mut) with the single predicted miRNA-378a-3p binding site, wild-type ASPM (ASPM WT), mutant ASPM (ASPM Mut) with all three predicted miRNA-378a-3p binding sites were amplified/synthesized, and inserted into pGL3-promoter vectors (Promega, WI, USA). The circGPC3 Mut and ASPM Mut were synthesized by IDT (IDT corporation, US). The plasmid pRL-TK with Renilla luciferase (Rluc) was used as the internal control (Promega, WI, USA). The constructed pGL3-promoter vectors were co-transfected with miR-378a-3p mimics and control miRNA into HEK293 cells. Thirty-six hours after transfection, firefly and Renilla luciferase activities were measured continuously using a dual luciferase reporter assay system (Promega, WI, USA) according to the manufacturer's instructions. The firefly luciferase activities were normalized to Renilla luciferase activity.

RNA IMMUNOPRECIPITATION (RIP) ASSAY

The RIP assay was performed as per manufacturer's instruction (MagnaRIP RNA-Binding Protein Immunoprecipitation Kit (Millipore, MA, USA)). Lysates from $1x10^7$ Huh7 cells were incubated with beads coated with 5 μg of control rabbit IgG or antibody against Argonaute-2 (AGO2) (Sigma Aldrich, St. Louis, MO, USA) with rotation at 4 °C overnight. Thereafter, total RNA was isolated for the detection of circRNAs and miRNA expression by qRT-PCR.

BIOTINYLATED RNA PULL-DOWN ASSAY

The pull-down assay was performed with biotinylated RNA probe as previously described with minor modification [64]. The biotinylated-circGPC3 probe and/or control probe were incubated with M280 magnetic beads (Life Technologies, Carlsbad, CA, USA) at room temperature for 2 h to generate probe-coated beads. Huh7 lysates was incubated at room

temperature overnight with the 3'-terminal-biotinylated-circGPC3 probe and control probe. Both circGPC3-expressing LO2 and vector control LO2s' cell-lysates were probed with 3′ - terminal-biotinylated-circGPC3 probe. Then RNAs were eluted with the wash buffer from the M280 magnetic beads and analyzed by qRT-PCR. Biotinylated-circGPC3 probe and control probe (Supporting Table S2) were synthesized by IDT (IDT corporation, US).

WESTERN BLOT ANALYSIS

Briefly, cells were first homogenized and lysed with RIPA buffer (Sigma Aldrich, St. Louis, MO, USA) supplemented with protease and phosphatase inhibitor cocktail (Roche, Germany). The lysed cells were sonicated using the Bioruptor sonicator (Diagenode) at 'high' setting for 5 minutes at intervals of 30 secs 'on' and 30 secs 'off' and centrifuged at 13,000 rpm at 4 °C for 20 minutes to obtain the total cell lysate. Total proteins were resolved with sodium dodecyl sulfate polyacrylamide gel electrophoresis (SDS-PAGE) and transferred to polyvinylidene difluoride (PVDF) membranes. The blots were blocked with 2 % ECLTM advanced blocking agent (GE health) in 1X PBS with 0.1 % Tween-20 and probed with corresponding primary antibodies (ASPM (26223-1- AP, Proteintech, USA), alpha-tubulin (sc-8035, Santa Cruz, USA), GPC3 (sc-65443, Santa Cruz, USA), β-catenin (sc-7963, Santa Cruz, USA), followed by incubation with secondary detection antibodies (Horseradish peroxidase (HRP) conjugated goat-anti-rabbit or goat-anti- mouse secondary antibody). The blots were then washed and visualized with Enhanced Chemiluminescence Reagent Kit (Amersham Bioscience, UK).

PLASMA RNA EXTRACTION

Total plasma RNA was extracted using TRIzol LS Reagent (Invitrogen, Carlsbad, CA), according to the manufacturer's instructions. As an internal control, 200 ng RNA fragment from D. melanogaster was artificially added into each plasma (400 μL) before RNA extraction.

The purity and concentration of RNA samples were determined with the NanoDrop ND-1000 spectrometer (Nanodrop Products, USA).

RNA SEQUENCING OF CELLS TRANSFECTED WITH THE VARIOUS NODAL circRNAs or si-RNAs AGAINST THE VARIOUS circRNAs

Total RNA extraction of cells was carried out using RNeasy Mini Kit (Qiagen, Germany) according to manufacturer's protocol. Quality of total RNA was evaluated using Nanodrop, Agarose gel electrophoresis and Agilent 2100. The first cDNA strand was synthesized using random hexamers and M-MuLV reverse transcriptase. The second cDNA strand was synthesized using DNA Polymerase I after RNase H treatment. The cDNAs are ligated with sequencing adaptors. Selection of fragments of 150-200 bp length was then performed using AMPure XP system (Beckman Coulter, USA). The selected fragments were then sequenced (Illumina Inc, USA). The raw sequencing was filtered before mapping the clean raw reads to human reference genome using STAR software. Differential analysis was carried out by EdgeR R package.

GENE ONTOLOGY AND PATHWAY ANALYSIS

Gene ontology and pathway analysis were performed using ConsensusPathDB based on the Kyoto Encyclopedia of Genes and Genomes (KEGG) [66] and Reactome pathways [67] databases. The enriched pathways with a value of $p<0.01\&q<0.05$ are considered significant. For common pathways of 5 key nodes, the enriched pathways with a value of $p<0.01$ are considered significant.

STATISTICAL ANALYSES

Experimental results are presented as the means ± standard error of three biological replicates. Statistical significance is indicated if P value < 0.05 using the Student's t-test which was used to compare the difference between the experimental group and the control.

Figure S1

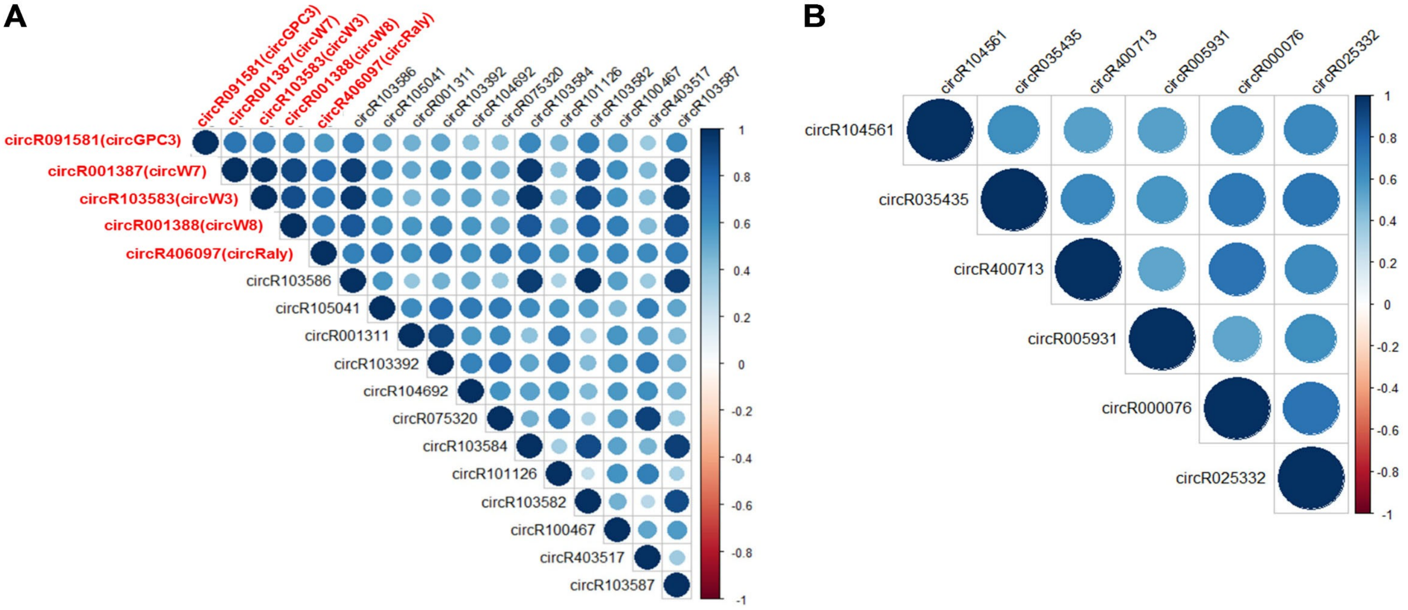

Figure S1. Correlation matrix plots for nodes in the prognostic circRNA-mRNA co-expressed networks. **A,** Correlation matrix for 17 worse prognostic circRNAs (including the five nodal/master circRNAs with red and bold labels). **B,** Correlation matrix for 6 better prognostic circRNAs.

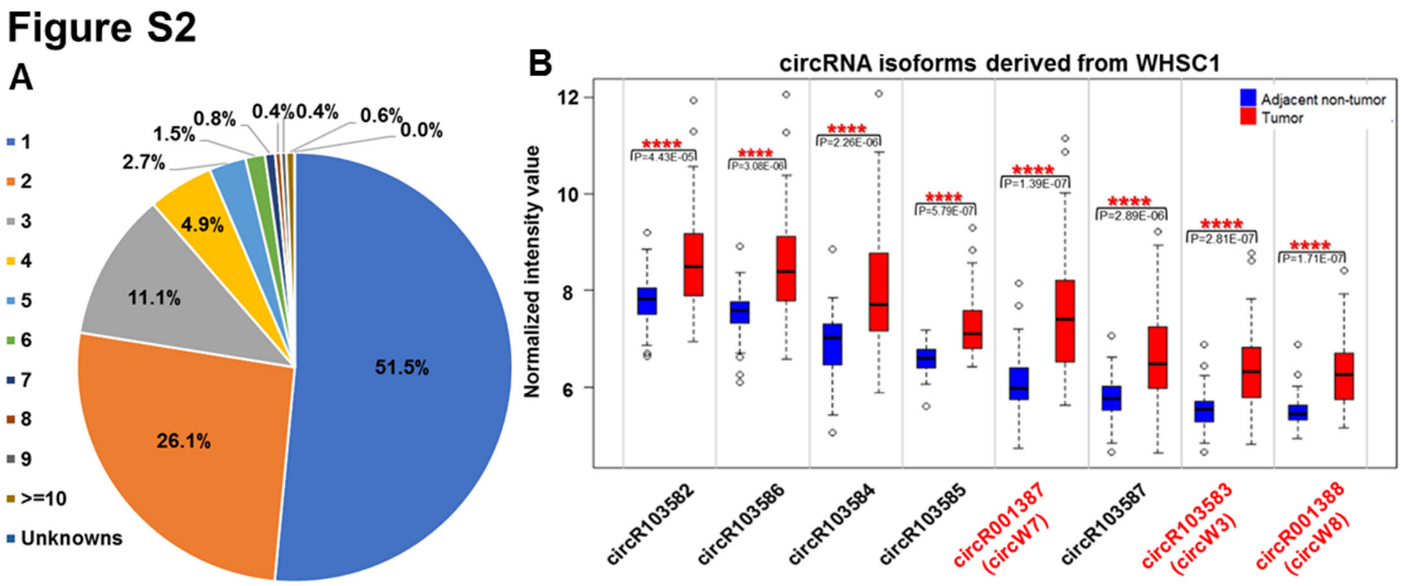

Figure S2. Distribution and example of circRNAs' isoforms. A, Numbers of circRNAs produced by the same gene. **B,** Eight alternative circRNA isoforms derived from the same gene WHSC1(including three of the oncogenic nodal circRNAs with red labels). Box plots showing the abundances of differentially expressed circRNA isoforms.

Figure S3

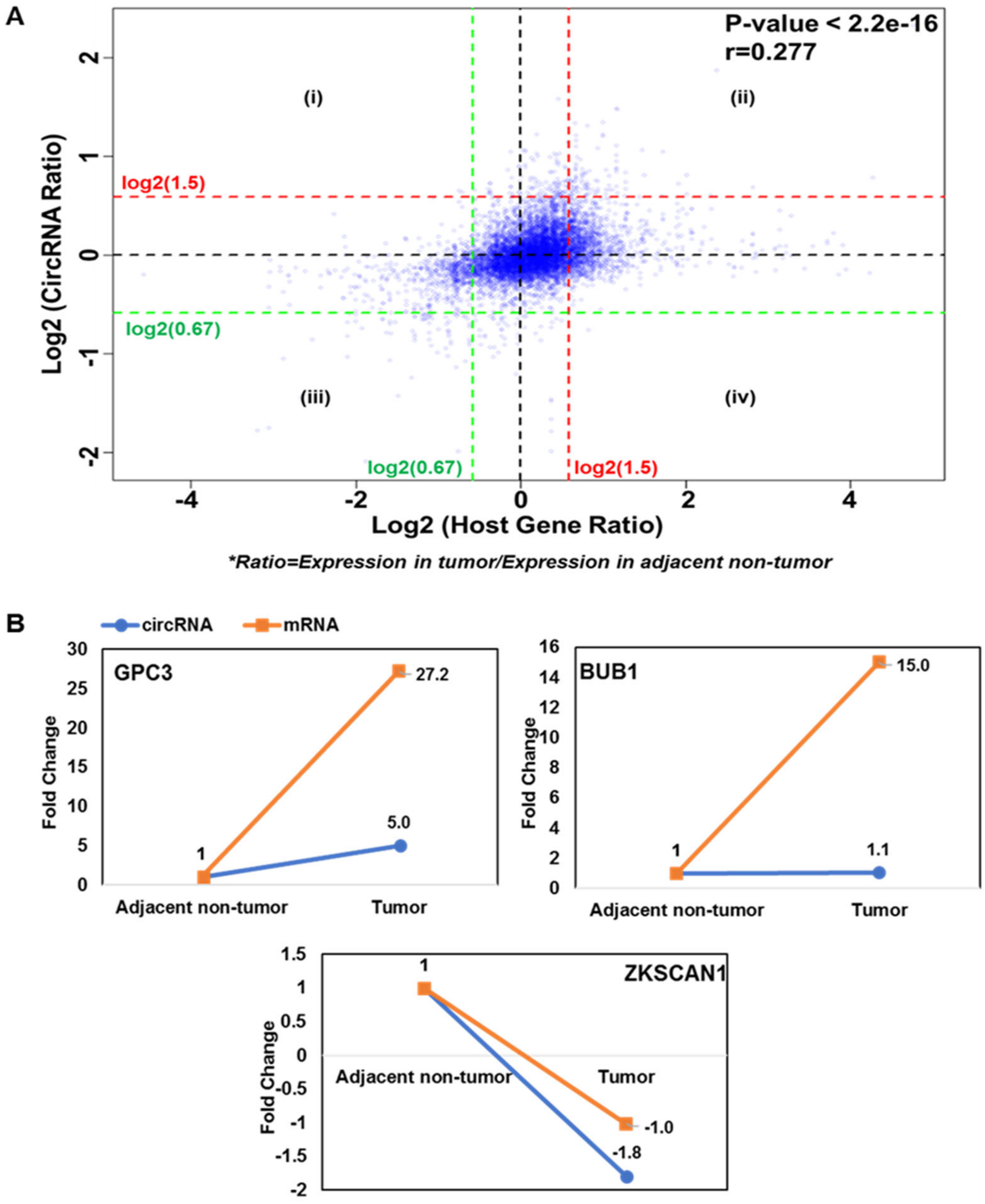

Figure S3. Correlation between the expression of circRNA and mRNA. A, circRNAs' expression change between tumor and adjacent non-tumor is generally positively correlated with the expression change of host genes (r=0.277, p=2.2e-16), with many exemptions. **B,** Examples of circRNAs and their parental mRNA/transcript whose expression are not correlated.

Figure S4

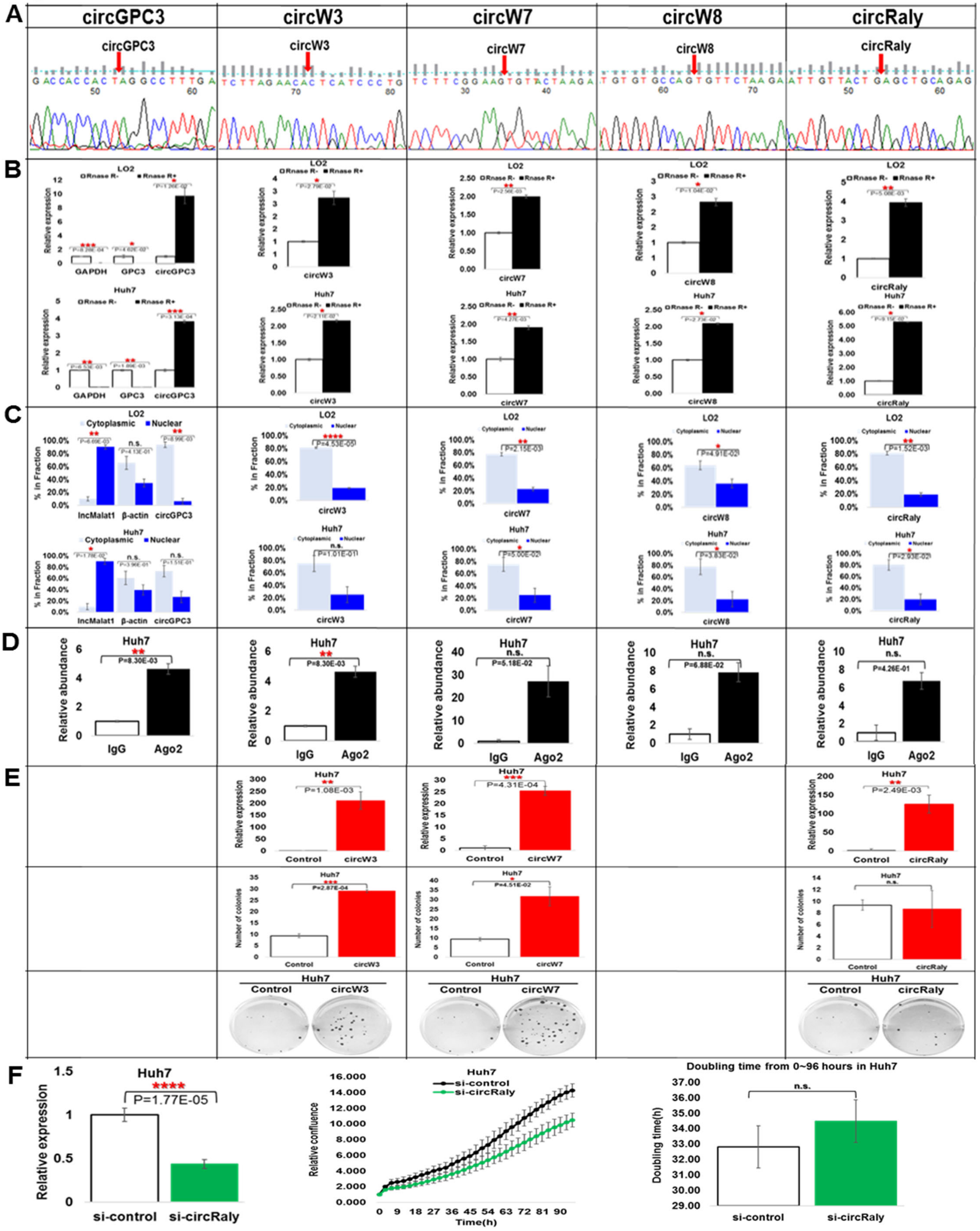
A
circGPC3
circW3
circW7
circW8
circRaly
B
C
D
E
F
Huh7
Relative expression
si-control
si-circRaly
P=1.77E-05
Relative confluence
Time(h)
Doubling time from 0~96 hours in Huh7
Doubling time(h)
n.s.
Relative abundance
IgG
Ago2
Control
Number of colonies
% in Fraction
Cytoplasmic
Nuclear
LO2
lncMalat1
β-actin
GAPDH
GPC3

Figure S4. Validation of five nodal circRNAs. A, Sanger sequencing of the junction site of circGPC3, circW3, circW7, circW8 and circRaly. **B,** Resistance to Rnase R digestion of LO2 / Huh 7 expressing the five nodal circRNAs or GPC3/GAPDH genes. **C,** Sub-cellular fractionation of circGPC3, circW3, circW7, circW8 and circRaly in LO2 and Huh7. **D,** Binding of the various circRNAs to AGO2 in Huh7 cells. **E,** Top panel: Expression of the 3 nodal circRNAs in Huh7 transfected with the respective circRNAs (circW3, circW7 and circRaly). Middle panel: Number of colonies in soft agar in cells transfected with the 3 nodal circRNAs. Bottom panel: Representative figures taken from soft agar plates. **F,** Cell proliferation of Huh7 cells transfected with si-circRaly.

Figure S5

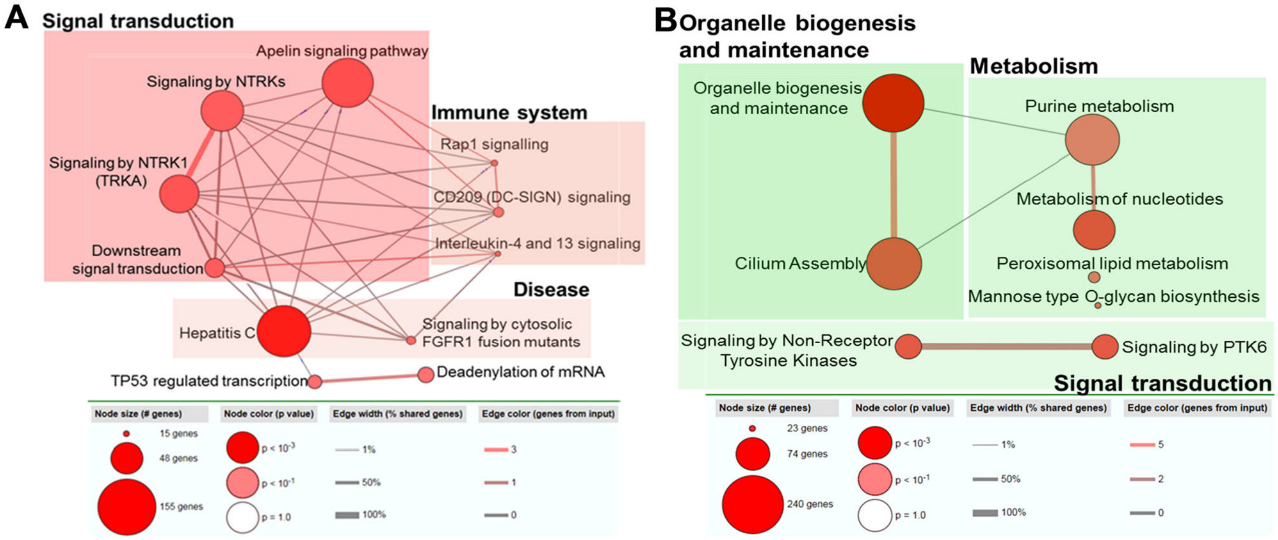

Figure S5. Network of pathways of genes that are commonly and consistently deregulated in LO2 / Huh7 cells transfected with either the respective cirRNAs or siRNA against the circRNAs.
A, Network of pathways of 98 upregulated genes that are commonly associated with the 5 nodal circRNAs.
B, Network of pathways of 96 down-regulated genes that are commonly associated with 5 nodal circRNAs.

Figure S6

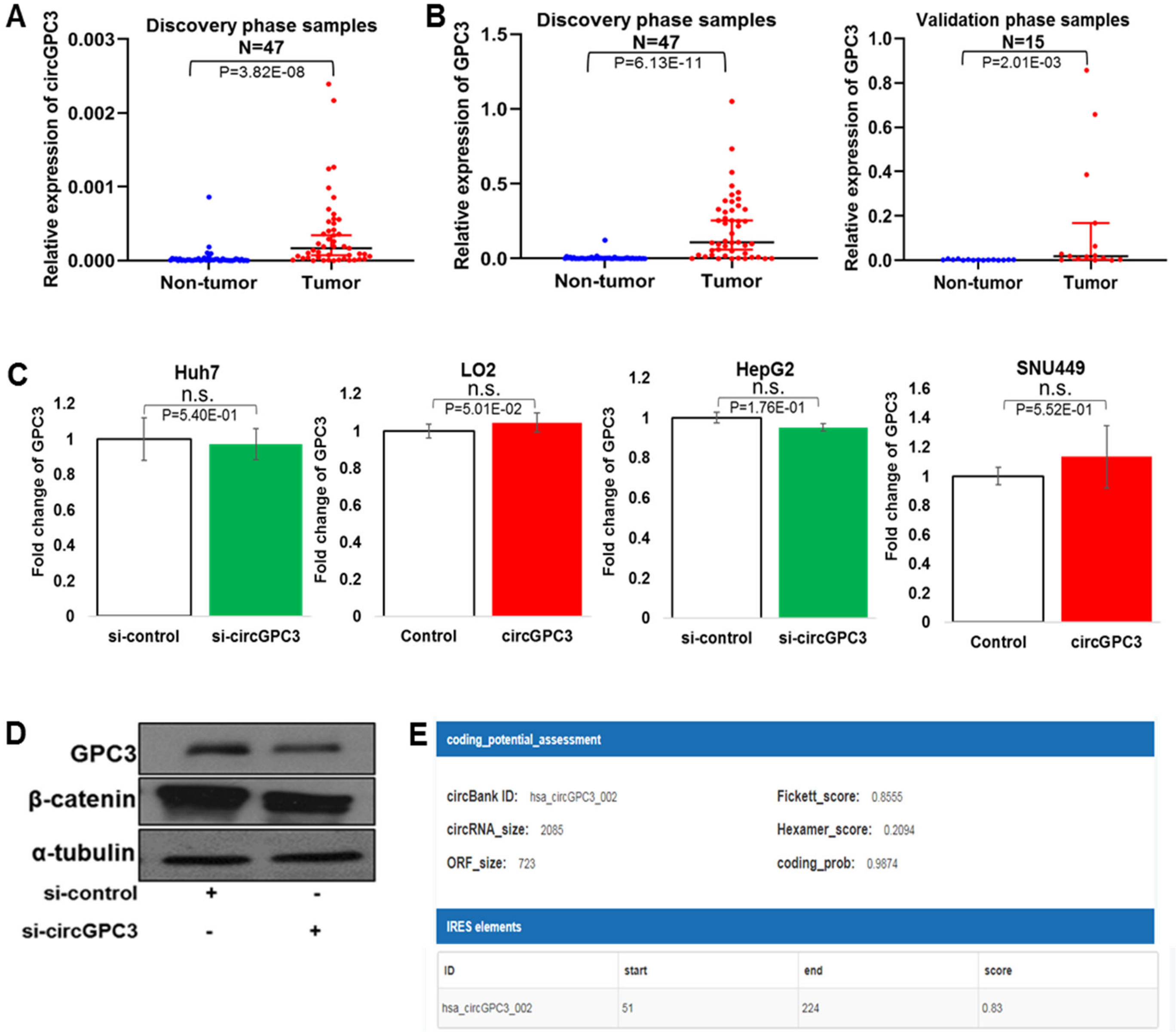

Figure S6. Host gene GPC3 of circGPC3. A, Expression of circGPC3 in 47 discovery phase samples from HCC patients. **B,** Expression of GPC3 in 47 discovery phase samples and 15 additional independent samples from HCC patients. **C,** The expression of GPC3 after circGPC3 knockdown in Huh7 and HepG2, and circGPC3-overexpressed LO2 and SNU449 cells. **D,** Protein levels of GPC3 and β-catenin in Huh7 cells transfected with si-circGPC3. **E,** Predicted translation probability of circGPC3 using http://www.circbank.cn/.

Figure S7

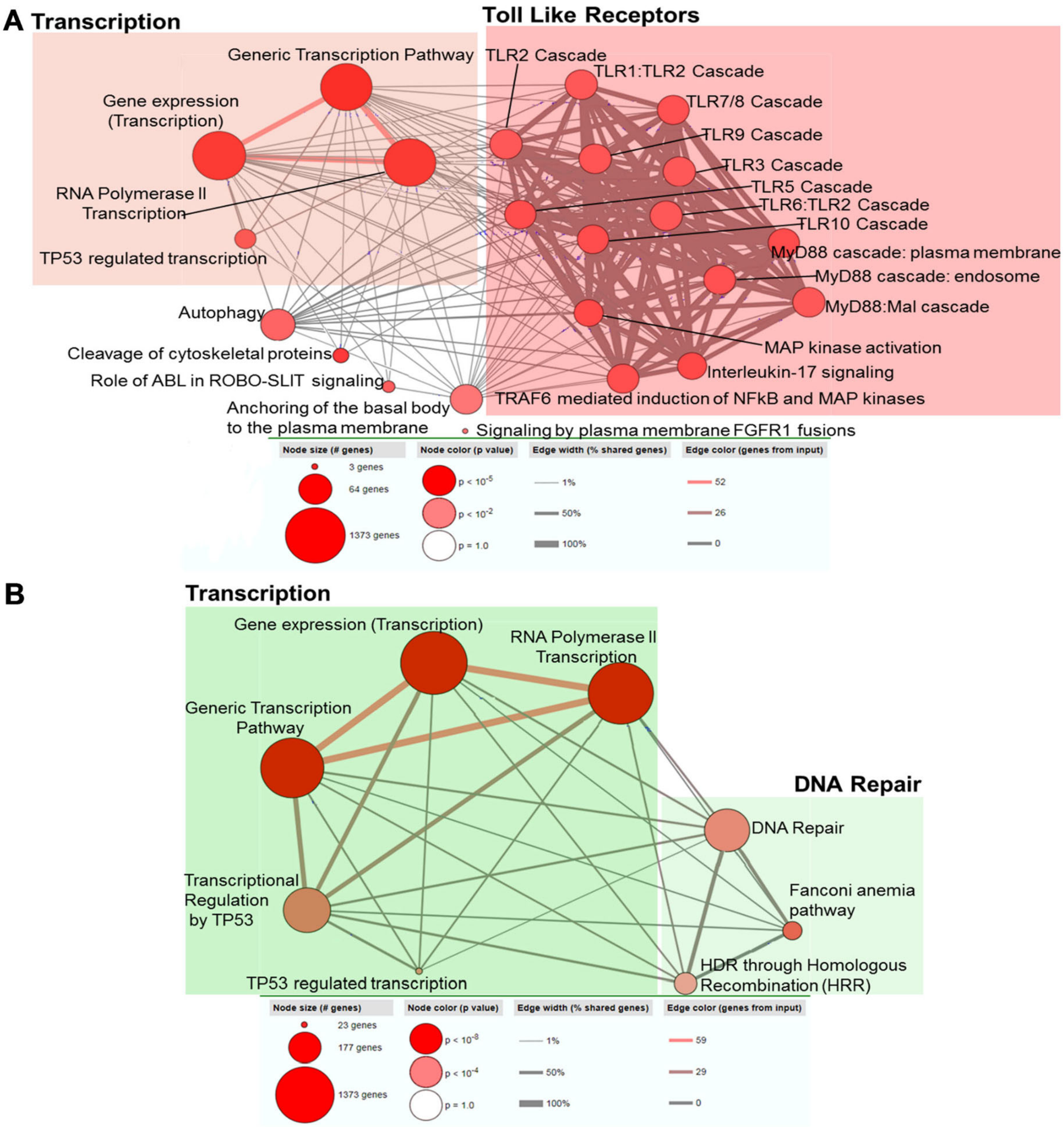

Figure S7. Network of pathways of genes deregulated in LO2 cells transfected with circGPC3 and Huh7 cells transfected with si-circGPC3. A, Pathways of the 467 up-regulated genes that are associated with circGPC3 both in LO2 which overexpressed circGPC3 and Huh7 where the expression of circGPC3 is inhibited. **B,** Pathways of the 409 down-regulated genes that are associated with circGPC3 both in LO2 which overexpressed circGPC3 and Huh7 where the expression of circGPC3 is inhibited.

Figure S8

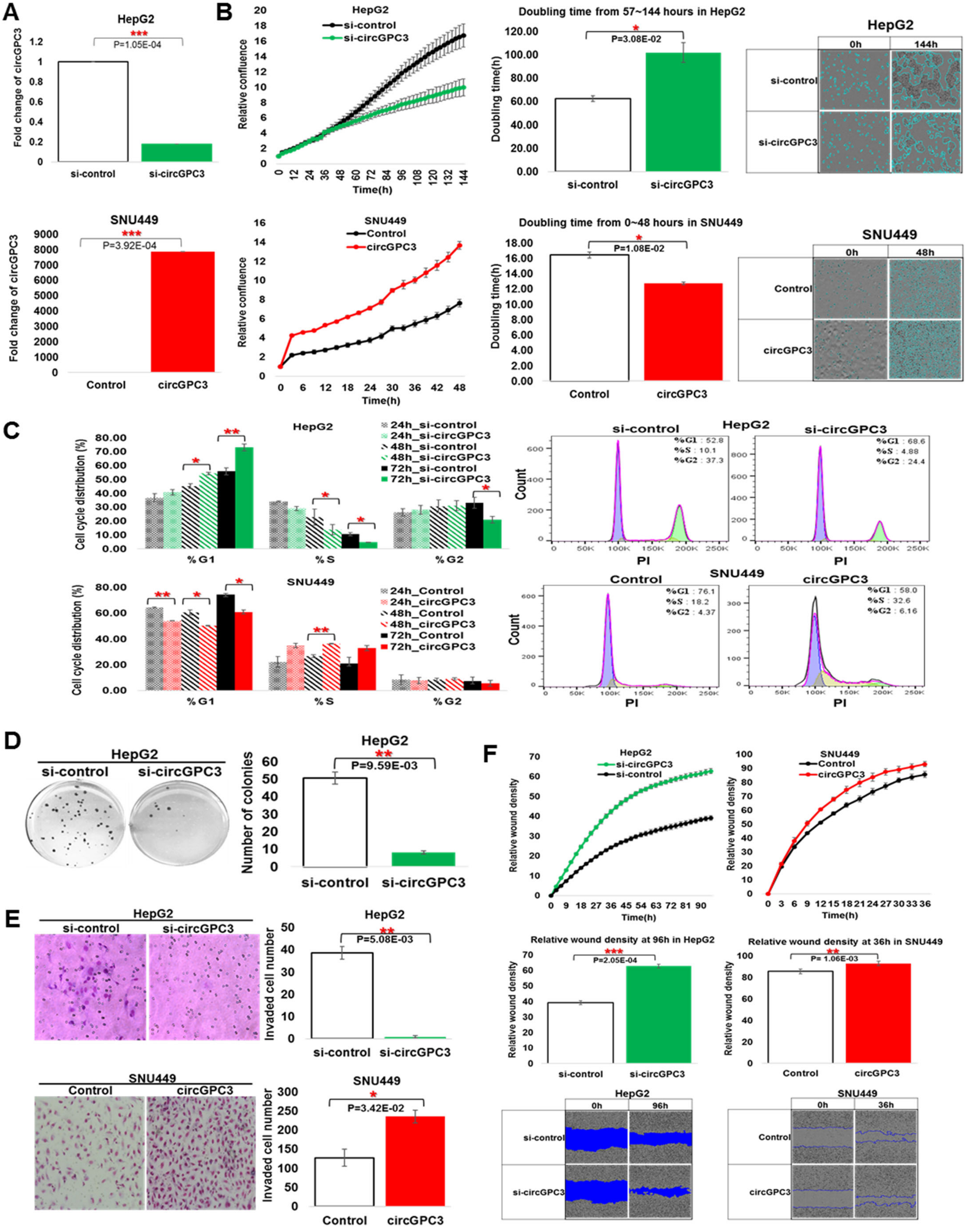
A
HepG2
P=1.05E-04
Fold change of circGPC3
si-control
si-circGPC3
SNU449
P=3.92E-04
Control
circGPC3
B
HepG2
si-control
si-circGPC3
Relative confluence
Time(h)
Doubling time from 57~144 hours in HepG2
P=3.08E-02
Doubling time(h)
0h
144h
SNU449
Control
circGPC3
Doubling time from 0~48 hours in SNU449
P=1.08E-02
48h
C
HepG2
24h_si-control
24h_si-circGPC3
48h_si-control
48h_si-circGPC3
72h_si-control
72h_si-circGPC3
Cell cycle distribution (%)
% G1
% S
% G2
Count
PI
SNU449
24h_Control
24h_circGPC3
48h_Control
48h_circGPC3
72h_Control
72h_circGPC3
D
HepG2
si-control
si-circGPC3
Number of colonies
P=9.59E-03
E
HepG2
Invaded cell number
P=5.08E-03
SNU449
Control
circGPC3
P=3.42E-02
F
HepG2
Relative wound density
Time(h)
SNU449
Relative wound density at 96h in HepG2
P=2.05E-04
Relative wound density at 36h in SNU449
P= 1.06E-03
0h
96h
36h

Figure S8. Experimental characterization of circGPC3 in HepG2 and SNU449. A, Expression of circGPC3 in HepG2 cells transfected with si-circGPC3 and in SNU449 cells transfected with circGPC3.**B,** Cell proliferation of HepG2 cells transfected with si-circGPC3 and SNU449 cells transfected with circGPC3. **C,** Cell-cycle phase distribution of HepG2 and SNU449 after knockdown (KD) and overexpression (OE) of circGPC3, respectively. **D,** Soft-agar colony formation of HepG2 cells transfected with si-circGPC3. **E,** Cell invasion assay of HepG2 and SNU449 cells after KD and OE of circGPC3. **F,** Cell migration for HepG2 and SNU449 cells after KD and OE of circGPC3.

Figure S9

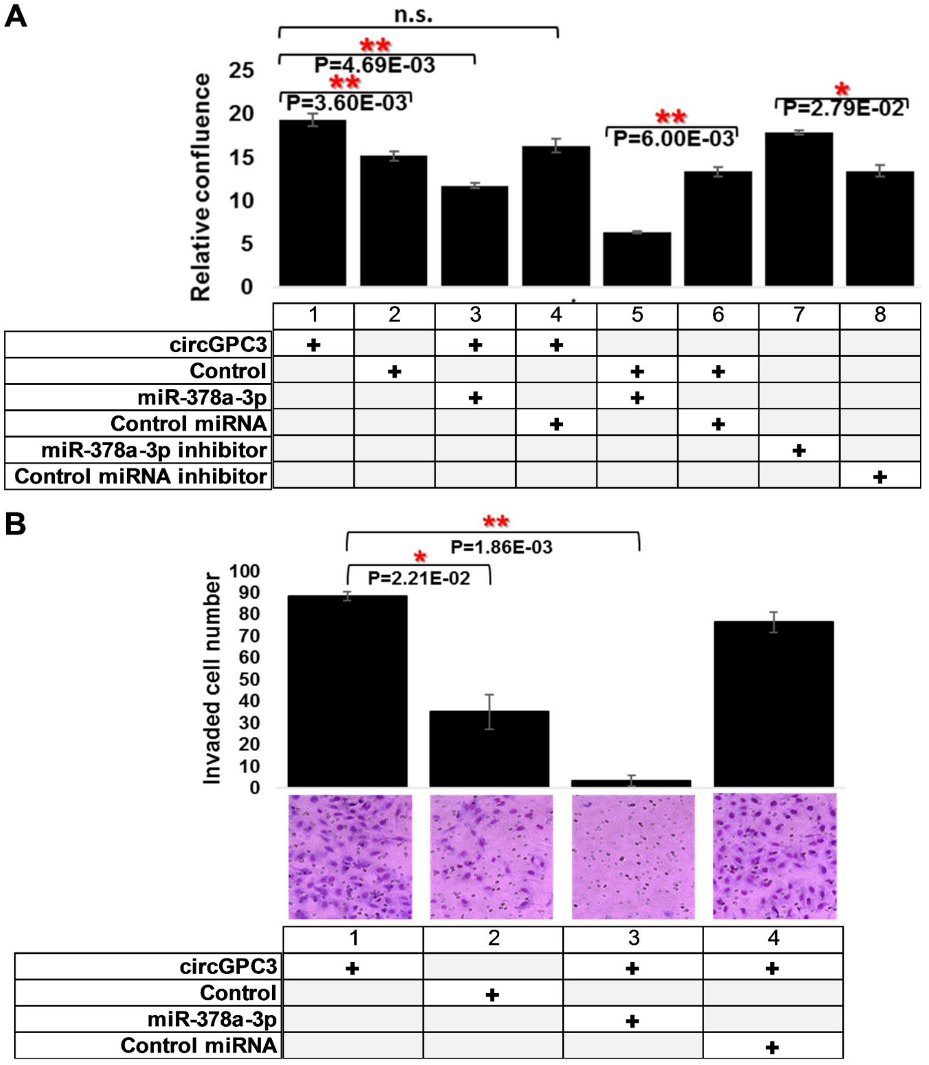

Figure S9. CircGPC3 acts as the sponge of miR-378a-3p to regulate cell proliferation and invasion. **A,** CircGPC3 acts as the sponge of miR-378a-3p to regulate cell proliferation in LO2. **B,** CircGPC3 acts as the sponge of miR-378a-3p to regulate cell invasion in LO2.

Figure S10

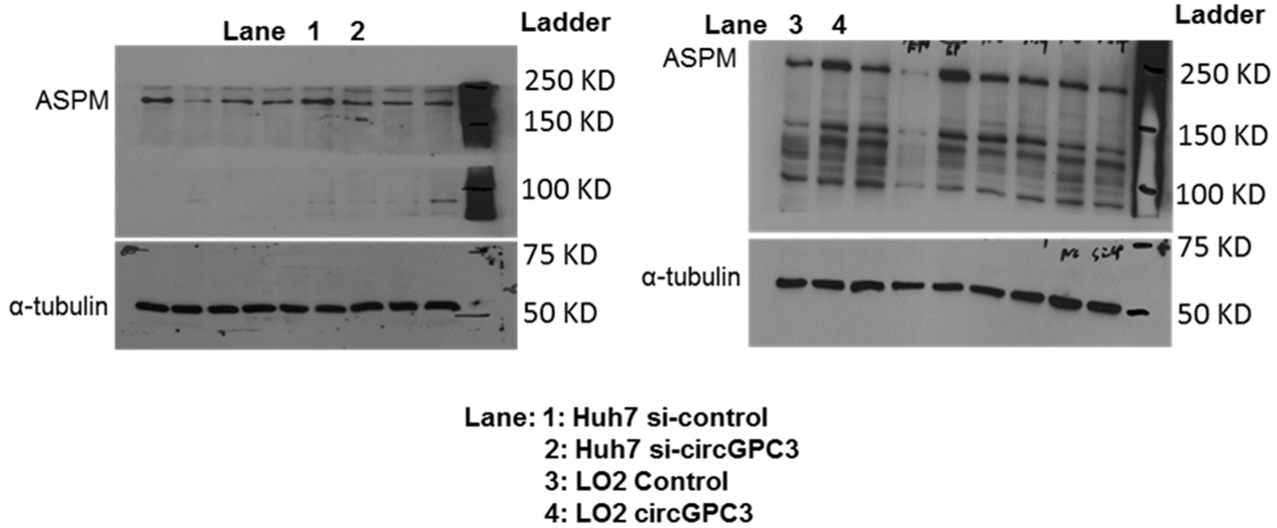

Figure S10. Raw Western blot data related to Figure 6F.

Figure S11

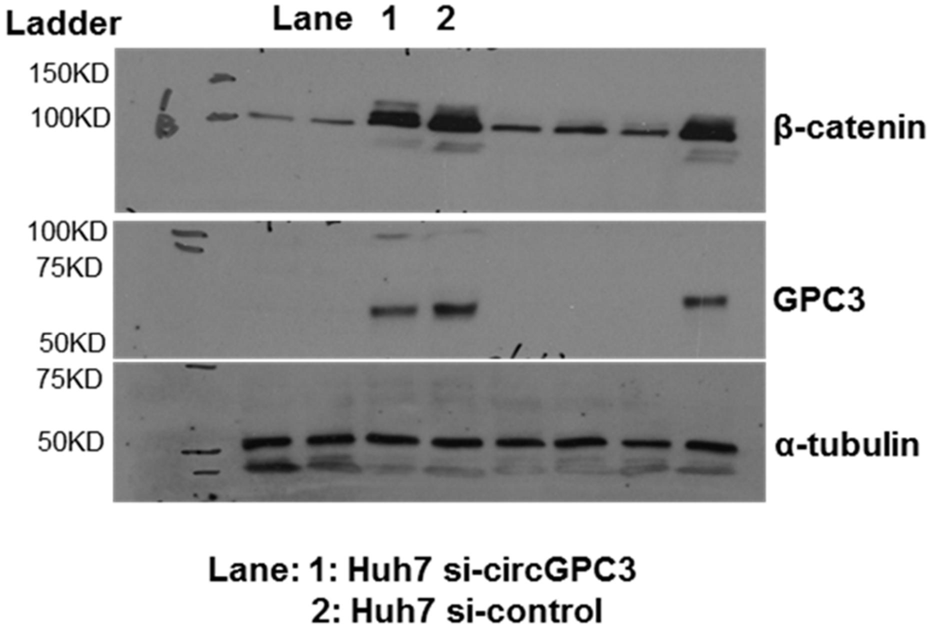

Figure S11. Raw Western blot data related to Figure S6D.

Table S1. Demographic and clinical information of 49 HCC patients.

Clinical phenotypes		Groups	Number of patients	Percentage of patients
General Characteristics	Age	>60	33	67%
		<=60	16	33%
	Gender	Male	35	71%
		Female	14	29%
	Race	Chinese	45	92%
		Indian	2	4%
		Others	2	4%
Tumor properties	Tumor size	<=5cm	25	51%
		>5cm	24	49%
	Tumor grade	Low (1,2)	22	45%
		High (3,4)	27	55%
	Encapsulation	Yes	33	67%
		No	16	33%
	Degree of encapsulation	Complete	15	31%
		Incomplete	18	37%
		Unknown	16	33%
Invasion and metastasis	Vascular invasion	No	27	55%
		Yes	22	45%
	Tumor invasion	No	18	37%
		Yes	26	53%
		Unknown	5	10%
Cancer stage and Survival	Cancer stage	Low (1,2)	37	76%
		High (3,4)	12	24%
	Overall survival	Alive	35	71%
		Deceased	10	20%
		Unknown	4	8%

FOOTNOTE: Yellow: Tumor properties (including Tumor size, Tumor Grade, Encapsulation and Degree of encapsulation); Blue: Invasion and metastasis (including Vascular invasion and Tumor invasion); Purple: Cancer stage and overall survival (including Cancer stage and Survival). Light orange background for groups: Better prognostic clinical features; Light grey background for groups: Worse prognostic clinical features.

Table S2. Primers, siRNAs and probes used in this study.

Name	Primers for circRNAs, genes, miRNAs	
	F	R
circGPC3 Divergent primer	AGAACGTACTGCTTGGTCTCTTTT	GAACAACAATTTCAAAGGCCTAGT
circGPC3 Convergent primer	CAAATACTTTCAGGTCACGTCTTG	AGCCTGTTTCCAGTCATCTATACC
GPC3	AAC TCC GAA GGA CAA CGA GA	GCA CCA GGA AGA AGA AGC AC
circW3 Divergent primer	TTTTTGGTCTTCTGTCAAAAACAC	CCTGCTTCATCTTTATGCACTTTA
circW7 Divergent primer	TTTTTGGTCTTCTGTCAAAAACAC	AAGCTACGAGGCTCTTCTCAAATA
circW8 Divergent primer	CCATACGAAAGTGCAGACGA	CCCAGATGCTTCCGTTCTTA
circRaly Divergent primer	GGT GAG CCC TAT TCC CAG AG	GAA CAA AGC AGC AAG CAC AA
lncMalat1	AAA GCA AGG TCT CCC CAC AAG	GGT CTG TGC TAG ATC AAA AGG CA
β-actin	GGG AAA TCG TGC GTG ACA TTA AG	TGT GTT GGC GTA CAG GTC TTT G
GAPDH	TGCACCACCAACTGCTTAGC	GGCATGGACTGTGGTCATGAG
ASPM	TGCAGTGGGTGAACATGAAAA	CGAAGAGGGTGTTACCTCGTTT
CENPW	AAGCCTCAACTTCGTCTGGAG	CACAAGCGTTTGTCCTGGACT
KIF14	TGTAGGTAGATTGGCACTTCAGA	CGACGTTGTAATGTAAGACGTGT
NEK2	GATGACTCAGTTGACTCTGGC	TCCCGATGCAATACGGTATGA
POLQ	CTG CGT CGG AGT GGG AAA C	CTG TAG GCT TGC ATT CTC CTG
TOP2A	ACCATTGCAGCCTGTAAATGA	GGGCGGAGCAAAATATGTTCC
DBF4	GGG CAA AAG AGT TGG TAG TGG	ACT TAT CGC CAT CTG TTT GGA TT
ERCC6L	CAAGGATGAACGGACCAGAAA	GCTTGAAAGTTGCTGCCAGTTA
E2F7	AAAGGGACTATTCCGACCCAT	ACTTGGATAGCGAGCTAGAAACT
GPC3	AAC TCC GAA GGA CAA CGA GA	GCA CCA GGA AGA AGA AGC AC
MMS22L	TGG ACA CCT ATC TGA ACT TCC C	ATG CCA TGA CGG TGG AAA AAG
cANRIL	GCTGGGATTACAGGTGTGAGACACC	GAATCAGAATGAGGCTTATTCTTCTCATC
miR-378a-3p	cat no: 001314	
RNU48	cat no: 001006	

Name	siRNA, mimic and inhibitor for miRNAs	
	sense（5'-3'）	antisense（5'-3'）
si-circGPC3	GACCACCACUAGGCCUUUGTT	CAAAGGCCUAGUGGUGGUCTT
si-circW8	UGUGCCAGUGUUCUAAGAATT	UUCUUAGAACACUGGCACATT
si-circRaly	CAUCAUUGUUACUGAGCUGTT	CAGCUCAGUAACAAUGAUGTT
NC	UUCUCCGAACGUGUCACGUTT	ACGUGACACGUUCGGAGAATT
hsa-mir-378a-3p mimics	ACUGGACUUGGAGUCAGAAGGC	CUUCUGACUCCAAGUCCAGUUU
hsa-mir-378a-3p inhibitor	GCCUUCUGACUCCAAGUCCAGU	
miRNA NC	UUCUCCGAACGUGUCACGU	

Name	Primers for full length amplication			Restriction digestion			T4 ligation		
	F	R	Size	Restriction enzymes	Time	Amount of DNA	Time	Amount of DNA	Vector DNA
circGPC3	*EcoRI* Original Primer<br>GG*GAATTC* **GACTATATGTATTGGCTGTAGGTC**	*BamHI* Original Primer<br>CG*GGATCC* **CATGGCTCTTCCACTAACTTTACG**	2055bp	EcoRI,BamHI	20 minutes	1ug	4°C, 16h	125.4 ng	100ng
circW3	*KpnI* Forward circularization mediating sequence Acceptor Original Primer<br>GGG *GTACC* TGAAATATGCTATCTTACAG**TGTTCTAAGAACGGAAGCAT**	*BamHI* Backward circularization mediating sequence Donor Original Primer<br>CG*GGATCC* TCAAGAAAAAATATATTCAC**CTCATCCCTGTGTTTTTGAC**	1439bp	KpnI, BamHI	20 minutes	1ug	4°C, 16h	116.0 ng	100ng
circW7	*KpnI* Forward circularization mediating sequence Acceptor Original Primer<br>GGG *GTACC* TGAAATATGCTATCTTACAG**TGTTCTAAGAACGGAAGCATCT**	*BamHI* Backward circularization mediating sequence Donor Original Primer<br>CG*GGATCC* TCAAGAAAAAATATATTCAC**CTTCCGAAGACTGTGCTTGTCC**	1703bp	KpnI, BamHI	20 minutes	1ug	4°C, 16h	137.3 ng	100ng
circW8	*KpnI* Forward circularization mediating sequence Acceptor Original Primer<br>GGG *GTACC* TGAAATATGCTATCTTACAG**TGTTCTAAGAACGGAAGCATCT**	*BamHI* Backward circularization mediating sequence Donor Original Primer<br>CG*GGATCC* TCAAGAAAAAATATATTCAC**CTGGCACACATACTCCTTTTTG**	2042bp	KpnI, BamHI	20 minutes	1ug	4°C, 16h	164.7 ng	100ng
circRaly	*KpnI* Forward circularization mediating sequence Acceptor Original Primer<br>GGG *GTACC* TGAAATATGCTATCTTACAG**AGCTGCAGAGATCCTTTGGTTC**	*BamHI* Backward circularization mediating sequence Donor Original Primer<br>CG*GGATCC* TCAAGAAAAAATATATTCAC**CAGTAACAATGATGAGCTTAGC**	1836bp	KpnI, BamHI	20 minutes	1ug	4°C, 16h	148.1 ng	100ng

Name	FISH probes
circGPC3	5'-AAAGGCCTAGTGGTGGTCAGCT-biotin-3'
Control	5'-TGGGCGT+AT+AGACGTGTT+ACAC-biotin-3'

Name	RNA pull down probes
circGPC3	5'-CGAACAACAATTTCAAAGGCCTAGTGGTGGTCAGCTTTCCTGCATTCT-biotin-3'
Control	5'-GCTTGTTGTTAAAGTTTCCGGATCACCACCAGTCGAAAGGACGTAAGA-biotin-3'

Table S3. 460 clinically-relevant co-expressed circRNA-mRNA pairs (|r|>0.7).

			Worse prognosis																	Better prognosis						Unclear				
		circRNA	circR103392	circR105041	circR001311	circR001387(circW7)	circR103584	circR103587	circR103582	circR001388	circR103580	circR103583	circR100467	circR091581	circR104692	circR075320	circR406097	circR403511	circR101120	circR035435	circR400713	circR000070	circR025333	circR005931	circR104561	circR400670	circR034537	circR103828	circR007940	circR100178
		Clinical association	TG DC CS OS	TG DC OS	TG DC OS	TG DC	TG DC	TG DC	TG DC	TG DC	TG DC	TG DC	TG	TG	TG OS	TG OS	TG OS	OS	CS	TS	TS	TS	TC	TC OS	OS	TC	TS	TC	TG TI	TG CS
Gene	Clinical association	FC	2.78	2.09	1.54	2.80	2.16	1.93	1.83	1.79	2.02	1.84	2.06	5.01	1.89	2.34	1.66	2.54	1.52	-1.82	-1.82	-3.17	-2.69	-1.84	-2.05	-4.30	-1.73	1.85	1.63	-2.07
FAM72B	TG DC	2.94	0.61	0.67	0.51	0.81	0.73	0.73	0.72	0.81	0.76	0.78	0.65	0.76	0.64	0.52	0.73	0.48	0.50	-0.49	-0.58	-0.61	-0.52	-0.42	-0.60	-0.52	-0.35	0.40	0.55	-0.65
FAM72A	TG DC	5.37	0.59	0.63	0.50	0.79	0.71	0.71	0.69	0.80	0.73	0.75	0.66	0.72	0.64	0.51	0.70	0.48	0.50	-0.47	-0.59	-0.60	-0.50	-0.42	-0.60	-0.52	-0.36	0.41	0.55	-0.63
KIF2A	TG DC OS	2.16	0.65	0.68	0.53	0.76	0.73	0.73	0.72	0.73	0.74	0.74	0.59	0.57	0.67	0.54	0.69	0.53	0.46	-0.37	-0.50	-0.43	-0.26	-0.36	-0.43	-0.42	-0.13	0.38	0.54	-0.51
MDK	TG	10.95	0.59	0.60	0.54	0.77	0.67	0.71	0.65	0.76	0.71	0.75	0.66	0.70	0.65	0.53	0.73	0.49	0.53	-0.49	-0.55	-0.59	-0.42	-0.36	-0.55	-0.52	-0.27	0.37	0.58	-0.60
SNRPE		2.17	0.55	0.58	0.50	0.78	0.70	0.71	0.70	0.76	0.72	0.77	0.64	0.74	0.57	0.45	0.64	0.43	0.49	-0.41	-0.53	-0.52	-0.46	-0.42	-0.56	-0.48	-0.28	0.36	0.45	-0.58
FAM72C	TG DC	5.95	0.61	0.65	0.52	0.78	0.70	0.69	0.68	0.80	0.72	0.74	0.67	0.73	0.64	0.53	0.71	0.48	0.50	-0.48	-0.61	-0.61	-0.51	-0.42	-0.60	-0.53	-0.33	0.41	0.55	-0.64
FAM72D	TG DC	5.86	0.61	0.64	0.52	0.78	0.70	0.70	0.67	0.79	0.72	0.74	0.66	0.73	0.64	0.53	0.70	0.48	0.51	-0.48	-0.60	-0.60	-0.50	-0.42	-0.59	-0.52	-0.32	0.41	0.55	-0.63
CENPL	TG	5.30	0.60	0.65	0.50	0.76	0.69	0.68	0.66	0.77	0.70	0.73	0.64	0.71	0.62	0.55	0.70	0.53	0.51	-0.46	-0.60	-0.62	-0.51	-0.42	-0.64	-0.56	-0.39	0.43	0.50	-0.59
MYBL2	TG	8.84	0.60	0.64	0.53	0.77	0.68	0.72	0.69	0.77	0.72	0.75	0.68	0.68	0.65	0.51	0.72	0.45	0.51	-0.52	-0.58	-0.64	-0.53	-0.43	-0.55	-0.47	-0.26	0.38	0.58	-0.55
FOXM1	TG	2.96	0.51	0.59	0.40	0.73	0.68	0.72	0.71	0.76	0.72	0.74	0.58	0.67	0.52	0.43	0.66	0.42	0.42	-0.45	-0.48	-0.54	-0.50	-0.45	-0.52	-0.40	-0.28	0.28	0.42	-0.56
PCNA	DC	2.58	0.52	0.63	0.40	0.74	0.73	0.70	0.74	0.71	0.76	0.74	0.53	0.63	0.53	0.46	0.69	0.43	0.43	-0.29	-0.46	-0.42	-0.40	-0.29	-0.46	-0.43	-0.30	0.35	0.46	-0.54
TXNRD1	TG DC CS OS	3.29	0.80	0.65	0.74	0.54	0.49	0.50	0.42	0.57	0.48	0.52	0.59	0.54	0.61	0.72	0.64	0.71	0.81	-0.43	-0.51	-0.40	-0.27	-0.33	-0.43	-0.57	-0.12	0.43	0.54	-0.52
TOP2A	TG DC	20.25	0.62	0.63	0.50	0.73	0.66	0.67	0.64	0.75	0.69	0.71	0.63	0.72	0.61	0.55	0.71	0.53	0.53	-0.48	-0.57	-0.61	-0.51	-0.42	-0.62	-0.57	-0.38	0.43	0.50	-0.59
CDCA3	TG	7.94	0.61	0.66	0.50	0.72	0.64	0.65	0.62	0.75	0.66	0.70	0.60	0.71	0.61	0.53	0.70	0.50	0.52	-0.54	-0.63	-0.64	-0.58	-0.45	-0.67	-0.53	-0.36	0.40	0.49	-0.65
HJURP	TG	8.40	0.62	0.64	0.52	0.76	0.69	0.70	0.65	0.78	0.70	0.74	0.65	0.70	0.62	0.55	0.69	0.53	0.51	-0.48	-0.55	-0.57	-0.48	-0.40	-0.58	-0.48	-0.26	0.44	0.52	-0.60
SNRPB	TG	2.45	0.63	0.64	0.55	0.74	0.68	0.70	0.70	0.75	0.73	0.73	0.62	0.63	0.59	0.51	0.70	0.49	0.52	-0.42	-0.56	-0.46	-0.42	-0.41	-0.52	-0.48	-0.17	0.36	0.57	-0.59
DTYMK	TG	2.88	0.56	0.63	0.45	0.73	0.69	0.69	0.68	0.71	0.72	0.72	0.60	0.63	0.64	0.50	0.73	0.48	0.46	-0.43	-0.52	-0.52	-0.48	-0.40	-0.56	-0.44	-0.28	0.40	0.52	-0.54
KIF20B		2.48	0.51	0.53	0.43	0.71	0.67	0.68	0.70	0.71	0.73	0.72	0.57	0.59	0.52	0.43	0.67	0.39	0.37	-0.39	-0.46	-0.49	-0.40	-0.28	-0.49	-0.35	-0.30	0.30	0.50	-0.58
CCT3		2.05	0.73	0.68	0.63	0.73	0.66	0.65	0.60	0.75	0.64	0.71	0.63	0.67	0.60	0.62	0.70	0.59	0.60	-0.45	-0.62	-0.52	-0.48	-0.39	-0.58	-0.51	-0.21	0.38	0.50	-0.65
TRIM16L	TG TC DC TI CS OS	4.38	0.84	0.72	0.75	0.52	0.46	0.46	0.39	0.56	0.43	0.49	0.51	0.54	0.61	0.67	0.62	0.64	0.75	-0.55	-0.56	-0.47	-0.38	-0.40	-0.61	-0.62	-0.23	0.45	0.42	-0.63
TKT	TG DC CS OS	2.25	0.86	0.73	0.75	0.47	0.41	0.42	0.36	0.54	0.39	0.44	0.52	0.46	0.69	0.61	0.59	0.61	0.71	-0.60	-0.59	-0.44	-0.39	-0.48	-0.56	-0.51	-0.09	0.43	0.55	-0.58
NEK2	TG DC	14.44	0.60	0.63	0.50	0.74	0.67	0.68	0.64	0.76	0.69	0.72	0.64	0.72	0.61	0.53	0.68	0.51	0.55	-0.54	-0.59	-0.62	-0.56	-0.47	-0.64	-0.56	-0.39	0.41	0.47	-0.63
EXO1	TG DC	16.36	0.63	0.63	0.52	0.73	0.65	0.67	0.62	0.74	0.67	0.71	0.64	0.70	0.65	0.55	0.71	0.54	0.57	-0.52	-0.59	-0.61	-0.52	-0.43	-0.62	-0.52	-0.32	0.40	0.52	-0.63
KIF2C	TG DC	6.45	0.61	0.65	0.50	0.73	0.68	0.69	0.67	0.74	0.71	0.73	0.63	0.67	0.63	0.52	0.70	0.51	0.53	-0.49	-0.56	-0.57	-0.51	-0.45	-0.60	-0.50	-0.33	0.38	0.54	-0.60
ASPM	TG	11.68	0.56	0.59	0.46	0.73	0.65	0.66	0.64	0.75	0.69	0.72	0.60	0.73	0.55	0.49	0.66	0.47	0.50	-0.46	-0.56	-0.62	-0.54	-0.40	-0.65	-0.53	-0.41	0.42	0.44	-0.62
MTFR2	TG	5.77	0.65	0.67	0.55	0.74	0.65	0.66	0.64	0.75	0.67	0.70	0.63	0.72	0.65	0.55	0.72	0.49	0.52	-0.53	-0.58	-0.60	-0.54	-0.47	-0.58	-0.54	-0.30	0.40	0.55	-0.61
KIF14	TG	6.87	0.53	0.58	0.46	0.73	0.64	0.65	0.62	0.74	0.67	0.71	0.65	0.72	0.56	0.49	0.63	0.46	0.48	-0.45	-0.54	-0.61	-0.52	-0.40	-0.56	-0.51	-0.34	0.38	0.48	-0.56
NUF2	TG	18.54	0.60	0.61	0.50	0.73	0.64	0.66	0.64	0.75	0.68	0.71	0.65	0.72	0.60	0.52	0.70	0.49	0.51	-0.51	-0.59	-0.62	-0.54	-0.44	-0.64	-0.55	-0.36	0.39	0.49	-0.63
DLGAP5	TG	7.35	0.58	0.61	0.48	0.75	0.67	0.70	0.66	0.75	0.70	0.73	0.62	0.69	0.58	0.48	0.68	0.46	0.45	-0.49	-0.57	-0.60	-0.51	-0.43	-0.59	-0.49	-0.32	0.38	0.52	-0.58
CCNB1	TG	9.04	0.63	0.64	0.53	0.73	0.66	0.67	0.64	0.75	0.68	0.71	0.63	0.68	0.67	0.58	0.70	0.54	0.54	-0.53	-0.60	-0.62	-0.53	-0.41	-0.62	-0.52	-0.32	0.38	0.55	-0.62
MCM6	TG	3.50	0.60	0.65	0.46	0.72	0.68	0.66	0.68	0.70	0.70	0.71	0.56	0.67	0.61	0.51	0.66	0.50	0.50	-0.41	-0.49	-0.49	-0.44	-0.43	-0.51	-0.40	-0.19	0.41	0.50	-0.56
GINS1	TG	3.88	0.57	0.62	0.48	0.72	0.66	0.70	0.64	0.73	0.69	0.71	0.64	0.67	0.62	0.50	0.67	0.48	0.50	-0.44	-0.55	-0.58	-0.50	-0.41	-0.55	-0.48	-0.33	0.41	0.49	-0.50
ARHGAP11A	TG	7.43	0.54	0.59	0.42	0.72	0.67	0.68	0.67	0.74	0.70	0.72	0.59	0.66	0.63	0.50	0.65	0.49	0.46	-0.46	-0.55	-0.56	-0.50	-0.41	-0.57	-0.46	-0.31	0.37	0.51	-0.59
DEPDC1		13.32	0.58	0.63	0.47	0.71	0.66	0.65	0.63	0.72	0.68	0.71	0.59	0.70	0.59	0.54	0.66	0.53	0.53	-0.46	-0.53	-0.56	-0.52	-0.42	-0.57	-0.56	-0.44	0.41	0.46	-0.58
FLVCR1		4.03	0.62	0.62	0.50	0.73	0.68	0.66	0.66	0.73	0.69	0.73	0.58	0.70	0.62	0.51	0.59	0.53	0.50	-0.48	-0.56	-0.52	-0.51	-0.43	-0.55	-0.50	-0.22	0.46	0.45	-0.59
SMC2		2.38	0.52	0.60	0.39	0.74	0.71	0.68	0.69	0.69	0.74	0.73	0.53	0.67	0.56	0.45	0.67	0.43	0.41	-0.29	-0.46	-0.44	-0.36	-0.28	-0.46	-0.46	-0.33	0.39	0.43	-0.50
AURKB		2.31	0.50	0.53	0.46	0.72	0.63	0.71	0.64	0.73	0.68	0.72	0.61	0.64	0.54	0.41	0.67	0.36	0.48	-0.55	-0.54	-0.67	-0.56	-0.43	-0.56	-0.39	-0.26	0.23	0.49	-0.57
SUCO	TG DC TI	2.52	0.65	0.56	0.56	0.73	0.68	0.68	0.65	0.71	0.69	0.73	0.64	0.67	0.59	0.52	0.62	0.51	0.51	-0.36	-0.52	-0.47	-0.35	-0.34	-0.48	-0.52	-0.25	0.44	0.44	-0.56
ENAH	TG DC TI	3.31	0.63	0.54	0.59	0.71	0.64	0.68	0.61	0.73	0.65	0.69	0.82	0.62	0.61	0.53	0.66	0.50	0.51	-0.37	-0.49	-0.52	-0.29	-0.38	-0.48	-0.43	-0.14	0.40	0.51	-0.51
MELK	TG DC	18.85	0.60	0.59	0.49	0.71	0.62	0.64	0.61	0.72	0.66	0.68	0.63	0.70	0.61	0.52	0.70	0.50	0.53	-0.50	-0.58	-0.63	-0.51	-0.40	-0.62	-0.55	-0.34	0.39	0.51	-0.59
KIF15	TG DC	7.20	0.60	0.60	0.54	0.73	0.64	0.67	0.60	0.73	0.66	0.70	0.69	0.70	0.58	0.56	0.72	0.52	0.55	-0.43	-0.54	-0.56	-0.43	-0.36	-0.56	-0.53	-0.35	0.41	0.54	-0.56
BUB1	TG DC	15.00	0.61	0.62	0.51	0.74	0.66	0.68	0.65	0.74	0.69	0.72	0.63	0.70	0.63	0.53	0.70	0.51	0.52	-0.50	-0.58	-0.61	-0.52	-0.44	-0.61	-0.52	-0.33	0.40	0.53	-0.59
RAD51	TG DC	4.52	0.60	0.64	0.48	0.71	0.66	0.67	0.66	0.74	0.69	0.70	0.58	0.62	0.66	0.47	0.67	0.45	0.51	-0.52	-0.58	-0.55	-0.53	-0.47	-0.58	-0.45	-0.27	0.35	0.56	-0.62
WHSC1	TG VI	2.24	0.43	0.44	0.36	0.67	0.63	0.71	0.66	0.70	0.70	0.70	0.57	0.51	0.47	0.33	0.57	0.34	0.38	-0.38	-0.44	-0.48	-0.37	-0.34	-0.40	-0.35	-0.27	0.31	0.38	-0.42
SQSTM1	TG OS	2.00	0.69	0.62	0.61	0.37	0.32	0.29	0.23	0.41	0.30	0.35	0.50	0.40	0.59	0.80	0.54	0.82	0.75	-0.44	-0.41	-0.37	-0.29	-0.27	-0.42	-0.54	-0.13	0.35	0.52	-0.44
CDKN3	TG	18.92	0.58	0.59	0.47	0.71	0.63	0.65	0.61	0.72	0.66	0.69	0.61	0.71	0.61	0.53	0.69	0.51	0.51	-0.53	-0.58	-0.64	-0.54	-0.46	-0.64	-0.55	-0.33	0.38	0.52	-0.60
PARPBP	TG	7.64	0.62	0.62	0.51	0.71	0.63	0.65	0.64	0.74	0.67	0.69	0.60	0.71	0.61	0.55	0.68	0.50	0.53	-0.51	-0.62	-0.64	-0.54	-0.45	-0.61	-0.58	-0.37	0.40	0.49	-0.61
TTK	TG	14.55	0.60	0.60	0.51	0.70	0.61	0.63	0.59	0.72	0.64	0.68	0.65	0.71	0.62	0.53	0.70	0.49	0.54	-0.51	-0.56	-0.62	-0.52	-0.43	-0.61	-0.53	-0.36	0.40	0.51	-0.60
DBF4	TG	2.05	0.55	0.60	0.48	0.72	0.63	0.65	0.65	0.71	0.68	0.68	0.55	0.70	0.58	0.44	0.63	0.40	0.43	-0.48	-0.53	-0.52	-0.49	-0.48	-0.51	-0.43	-0.16	0.36	0.57	-0.62
ERCC6L	TG	14.52	0.63	0.65	0.51	0.71	0.63	0.65	0.62	0.73	0.66	0.69	0.61	0.70	0.65	0.54	0.68	0.53	0.55	-0.54	-0.59	-0.61	-0.55	-0.47	-0.66	-0.53	-0.36	0.41	0.51	-0.65
UBE2T	TG	5.46	0.58	0.63	0.48	0.74	0.66	0.68	0.65	0.73	0.68	0.72	0.62	0.70	0.62	0.51	0.69	0.50	0.53	-0.49	-0.58	-0.62	-0.54	-0.43	-0.62	-0.53	-0.36	0.40	0.53	-0.62
PBK	TG	12.78	0.60	0.61	0.49	0.71	0.65	0.66	0.63	0.75	0.67	0.70	0.61	0.70	0.60	0.52	0.66	0.52	0.54	-0.51	-0.59	-0.60	-0.53	-0.42	-0.59	-0.59	-0.38	0.40	0.50	-0.59
CDC25A	TG	4.62	0.55	0.58	0.44	0.71	0.63	0.66	0.63	0.73	0.66	0.70	0.55	0.70	0.57	0.45	0.65	0.43	0.46	-0.48	-0.57	-0.56	-0.52	-0.38	-0.54	-0.40	-0.28	0.35	0.49	-0.59
CENPF	TG	17.43	0.54	0.56	0.45	0.72	0.63	0.67	0.62	0.73	0.67	0.71	0.64	0.69	0.58	0.48	0.67	0.47	0.50	-0.51	-0.57	-0.64	-0.53	-0.43	-0.65	-0.54	-0.40	0.37	0.48	-0.59
CENPE	TG	7.24	0.58	0.62	0.46	0.71	0.63	0.63	0.61	0.71	0.66	0.68	0.63	0.69	0.61	0.54	0.71	0.52	0.49	-0.46	-0.56	-0.59	-0.49	-0.41	-0.57	-0.49	-0.33	0.39	0.52	-0.60
CHEK1	TG	7.35	0.58	0.61	0.49	0.72	0.66	0.68	0.67	0.73	0.70	0.71	0.58	0.69	0.63	0.51	0.67	0.48	0.54	-0.49	-0.56	-0.57	-0.52	-0.42	-0.55	-0.48	-0.28	0.37	0.52	-0.59
UBE2C	TG	7.14	0.52	0.60	0.43	0.73	0.65	0.69	0.66	0.75	0.69	0.72	0.59	0.69	0.57	0.44	0.68	0.41	0.47	-0.54	-0.56	-0.65	-0.61	-0.47	-0.61	-0.47	-0.38	0.33	0.48	-0.59
GPSM2	TG	10.70	0.60	0.65	0.49	0.72	0.65	0.66	0.63	0.74	0.67	0.70	0.61	0.68	0.62	0.54	0.68	0.52	0.53	-0.53	-0.62	-0.62	-0.57	-0.48	-0.62	-0.52	-0.31	0.39	0.51	-0.62
CDC20	TG	10.36	0.57	0.62	0.49	0.72	0.63	0.67	0.63	0.73	0.67	0.70	0.66	0.68	0.62	0.51	0.69	0.49	0.53	-0.53	-0.57	-0.64	-0.55	-0.47	-0.64	-0.52	-0.35	0.38	0.52	-0.58
CDK1	TG	13.05	0.61	0.62	0.51	0.72	0.65	0.66	0.64	0.74	0.68	0.70	0.63	0.68	0.65	0.53	0.70	0.51	0.50	-0.52	-0.59	-0.60	-0.52	-0.42	-0.61	-0.49	-0.29	0.39	0.55	-0.61
ANLN	TG	14.48	0.60	0.62	0.49	0.73	0.65	0.67	0.64	0.75	0.69	0.71	0.65	0.68	0.62	0.53	0.69	0.52	0.52	-0.53	-0.58	-0.63	-0.53	-0.45	-0.63	-0.57	-0.35	0.36	0.52	-0.59
SHCBP1	TG	6.29	0.67	0.70	0.56	0.72	0.65	0.64	0.63	0.74	0.67	0.69	0.65	0.67	0.69	0.54	0.72	0.53	0.51	-0.53	-0.58	-0.55	-0.47	-0.43	-0.55	-0.48	-0.17	0.40	0.62	-0.59
LAGE3	TG	2.15	0.63	0.75	0.50	0.72	0.67	0.63	0.63	0.66	0.66	0.68	0.56	0.66	0.62	0.58	0.72	0.56	0.52	-0.33	-0.53	-0.48	-0.43	-0.37	-0.57	-0.51	-0.27	0.39	0.48	-0.52
GPD2	TG	2.03	0.62	0.63	0.53	0.74	0.69	0.69	0.70	0.69	0.73	0.71	0.62	0.66	0.64	0.50	0.64	0.47	0.43	-0.38	-0.49	-0.46	-0.30	-0.36	-0.40	-0.39	-0.09	0.40	0.57	-0.50
ORC1	TG	6.04	0.56	0.61	0.49	0.71	0.64	0.67	0.63	0.71	0.68	0.70	0.61	0.66	0.61	0.51	0.70	0.48	0.51	-0.46	-0.52	-0.59	-0.52	-0.43	-0.57	-0.48	-0.30	0.38	0.54	-0.55
PTTG1	TG	6.51	0.61	0.62	0.48	0.71	0.64	0.66	0.61	0.74	0.66	0.70	0.61	0.65	0.61	0.56	0.69	0.55	0.52	-0.53	-0.58	-0.59	-0.53	-0.42	-0.64	-0.53	-0.34	0.37	0.50	-0.63
KIF11	TG	3.76	0.54	0.61	0.43	0.73	0.67	0.67	0.66	0.73	0.70	0.72	0.54	0.65	0.57	0.46	0.61	0.45	0.48	-0.51	-0.56	-0.57	-0.52	-0.41	-0.60	-0.46	-0.36	0.37	0.50	-0.58
CEP55	TG	8.67	0.62	0.65	0.52	0.70	0.61	0.64	0.61	0.72	0.65	0.68	0.62	0.63	0.69	0.51	0.70	0.50	0.49	-0.57	-0.62	-0.64	-0.52	-0.45	-0.63	-0.45	-0.26	0.34	0.61	-0.64
LIN9	DC	4.24	0.52	0.55	0.38	0.72	0.67	0.67	0.69	0.73	0.70	0.72	0.56	0.67	0.54	0.43	0.62	0.41	0.44	-0.41	-0.54	-0.51	-0.49	-0.42	-0.61	-0.45	-0.35	0.32	0.35	-0.62
NUP155		2.12	0.61	0.60	0.49	0.75	0.67	0.68	0.68	0.70	0.70	0.72	0.58	0.73	0.55	0.46	0.67	0.41	0.48	-0.37	-0.51	-0.48	-0.40	-0.40	-0.52	-0.47	-0.24	0.36	0.43	-0.52
ARHGEF39		10.29	0.54	0.56	0.47	0.71	0.61	0.65	0.59	0.71	0.64	0.69	0.61	0.72	0.57	0.48	0.67	0.46	0.53	-0.54	-0.58	-0.67	-0.57	-0.42	-0.68	-0.55	-0.43	0.38	0.46	-0.59
INCENP		2.37	0.58	0.65	0.48	0.73	0.66	0.66	0.63	0.74	0.68	0.71	0.64	0.69	0.59	0.54	0.69	0.52	0.56	-0.45	-0.53	-0.57	-0.52	-0.37	-0.61	-0.52	-0.40	0.44	0.45	-0.60
MSH2		2.88	0.58	0.62	0.45	0.71	0.66	0.66	0.68	0.71	0.71	0.70	0.59	0.68	0.59	0.48	0.67	0.46	0.47	-0.38	-0.55	-0.49	-0.45	-0.38	-0.61	-0.51	-0.32	0.43	0.47	-0.55
PLK4		3.73	0.56	0.62	0.45	0.72	0.66	0.66	0.65	0.73	0.69	0.70	0.58	0.67	0.61	0.50	0.69	0.47	0.48	-0.47	-0.53	-0.59	-0.49	-0.40	-0.56	-0.45	-0.30	0.38	0.53	-0.59

**SUV39H2**		2.20	0.62	0.61	0.49	0.71	0.70	0.67	0.68	0.73	0.70	0.72	0.57	0.66	0.57	0.49	0.63	0.50	0.49	-0.37	-0.45	-0.39	-0.35	-0.37	-0.51	-0.48	-0.25	0.40	0.40	-0.56
**NCAPG2**		2.80	0.48	0.57	0.34	0.71	0.66	0.67	0.65	0.71	0.69	0.71	0.55	0.62	0.54	0.47	0.67	0.45	0.42	-0.40	-0.53	-0.53	-0.49	-0.34	-0.57	-0.48	-0.41	0.35	0.43	-0.55
**PSMD10**		2.06	0.68	0.69	0.53	0.72	0.69	0.66	0.65	0.71	0.67	0.71	0.51	0.59	0.62	0.50	0.68	0.51	0.44	-0.39	-0.53	-0.43	-0.34	-0.37	-0.54	-0.49	-0.22	0.44	0.52	-0.54
**DARS2**		2.18	0.51	0.46	0.43	0.70	0.70	0.70	0.64	0.65	0.70	0.72	0.55	0.58	0.48	0.51	0.58	0.52	0.45	-0.18	-0.41	-0.38	-0.25	-0.16	-0.38	-0.46	-0.41	0.43	0.40	-0.40
**CCT6A**		2.00	0.63	0.60	0.55	0.72	0.66	0.70	0.62	0.72	0.67	0.70	0.63	0.58	0.66	0.55	0.66	0.54	0.55	-0.48	-0.59	-0.50	-0.39	-0.42	-0.51	-0.49	-0.17	0.32	0.62	-0.56
**SF3B4**	TG DC TI	2.41	0.59	0.59	0.50	0.71	0.69	0.67	0.67	0.69	0.69	0.71	0.61	0.64	0.54	0.53	0.60	0.53	0.49	-0.34	-0.45	-0.41	-0.37	-0.32	-0.54	-0.45	-0.28	0.36	0.38	-0.59
**MTBP**	TG DC	3.76	0.57	0.56	0.47	0.70	0.63	0.66	0.64	0.73	0.66	0.69	0.56	0.61	0.56	0.47	0.68	0.42	0.46	-0.44	-0.61	-0.58	-0.51	-0.30	-0.63	-0.51	-0.43	0.36	0.44	-0.62
**G6PD**	TG OS	2.77	0.68	0.76	0.65	0.60	0.53	0.56	0.49	0.59	0.55	0.57	0.66	0.51	0.70	0.65	0.74	0.62	0.67	-0.37	-0.42	-0.40	-0.29	-0.35	-0.42	-0.40	-0.15	0.38	0.63	-0.44
**MFSD2A**	TG OS	-7.34	-0.61	-0.63	-0.52	-0.62	-0.51	-0.51	-0.52	-0.65	-0.53	-0.57	-0.47	-0.67	-0.59	-0.45	-0.55	-0.43	-0.50	0.71	0.64	0.60	0.63	0.49	0.67	0.43	0.17	-0.28	-0.46	0.88
**E2F7**	TG	14.41	0.55	0.59	0.44	0.69	0.62	0.63	0.61	0.70	0.65	0.68	0.58	0.71	0.58	0.51	0.67	0.47	0.49	-0.47	-0.56	-0.60	-0.54	-0.43	-0.58	-0.52	-0.35	0.38	0.46	-0.60
**POLQ**	TG	9.67	0.58	0.62	0.50	0.68	0.59	0.62	0.58	0.70	0.62	0.65	0.61	0.70	0.62	0.53	0.68	0.49	0.55	-0.52	-0.60	-0.64	-0.57	-0.43	-0.61	-0.53	-0.35	0.40	0.51	-0.59
**C5orf34**	TG	5.58	0.59	0.63	0.47	0.70	0.61	0.62	0.62	0.72	0.66	0.67	0.60	0.70	0.59	0.51	0.67	0.47	0.48	-0.48	-0.62	-0.59	-0.56	-0.43	-0.67	-0.58	-0.39	0.41	0.45	-0.64
**KIF18A**	TG	11.67	0.61	0.62	0.51	0.71	0.63	0.64	0.63	0.74	0.67	0.69	0.64	0.70	0.60	0.54	0.69	0.50	0.52	-0.51	-0.56	-0.61	-0.54	-0.44	-0.63	-0.54	-0.34	0.37	0.51	-0.64
**SPC25**	TG	12.84	0.61	0.63	0.50	0.70	0.63	0.64	0.60	0.72	0.65	0.68	0.59	0.69	0.63	0.54	0.67	0.54	0.54	-0.52	-0.57	-0.58	-0.53	-0.45	-0.63	-0.57	-0.34	0.40	0.51	-0.61
**HELLS**	TG	5.39	0.57	0.61	0.46	0.70	0.64	0.65	0.66	0.72	0.68	0.69	0.56	0.69	0.61	0.45	0.62	0.44	0.46	-0.52	-0.57	-0.60	-0.54	-0.47	-0.60	-0.41	-0.23	0.37	0.52	-0.64
**PRR11**	TG	2.72	0.54	0.65	0.45	0.72	0.66	0.67	0.67	0.71	0.69	0.69	0.61	0.69	0.60	0.49	0.69	0.44	0.47	-0.52	-0.53	-0.62	-0.54	-0.45	-0.60	-0.43	-0.31	0.33	0.50	-0.57
**E2F8**	TG	18.43	0.59	0.61	0.48	0.70	0.63	0.63	0.60	0.71	0.65	0.68	0.59	0.69	0.60	0.55	0.67	0.53	0.53	-0.46	-0.57	-0.58	-0.50	-0.41	-0.59	-0.53	-0.32	0.40	0.49	-0.59
**UCK2**	TG	2.72	0.67	0.60	0.62	0.71	0.62	0.67	0.61	0.72	0.65	0.69	0.69	0.68	0.60	0.55	0.66	0.54	0.65	-0.54	-0.57	-0.59	-0.46	-0.46	-0.57	-0.51	-0.19	0.36	0.52	-0.59
**MAD2L1**	TG	5.73	0.62	0.65	0.50	0.70	0.65	0.63	0.63	0.72	0.67	0.68	0.56	0.68	0.63	0.54	0.70	0.50	0.51	-0.47	-0.56	-0.56	-0.50	-0.42	-0.59	-0.51	-0.32	0.42	0.54	-0.61
**CCNB2**	TG	14.45	0.57	0.59	0.47	0.71	0.63	0.65	0.62	0.72	0.66	0.69	0.63	0.68	0.62	0.51	0.68	0.50	0.50	-0.52	-0.59	-0.63	-0.55	-0.43	-0.63	-0.51	-0.34	0.37	0.52	-0.60
**DTL**	TG	9.31	0.52	0.57	0.44	0.71	0.64	0.66	0.64	0.71	0.67	0.70	0.60	0.68	0.56	0.49	0.66	0.47	0.53	-0.45	-0.54	-0.60	-0.53	-0.38	-0.60	-0.49	-0.37	0.37	0.46	-0.55
**GTSE1**	TG	3.62	0.51	0.61	0.42	0.71	0.64	0.68	0.66	0.73	0.68	0.69	0.59	0.67	0.59	0.45	0.65	0.43	0.45	-0.52	-0.56	-0.63	-0.56	-0.50	-0.62	-0.41	-0.29	0.35	0.48	-0.61
**BUB1B**	TG	13.15	0.54	0.55	0.46	0.70	0.62	0.66	0.62	0.72	0.67	0.69	0.66	0.67	0.59	0.50	0.66	0.49	0.52	-0.49	-0.55	-0.62	-0.52	-0.44	-0.60	-0.55	-0.39	0.38	0.51	-0.57
**KIAA0101**	TG	4.98	0.61	0.70	0.50	0.71	0.65	0.65	0.64	0.73	0.66	0.70	0.55	0.66	0.64	0.50	0.67	0.50	0.51	-0.54	-0.60	-0.60	-0.58	-0.47	-0.63	-0.46	-0.29	0.36	0.57	-0.62
**MCM10**	TG	3.89	0.58	0.62	0.49	0.70	0.63	0.67	0.63	0.73	0.66	0.69	0.64	0.66	0.63	0.52	0.69	0.48	0.54	-0.50	-0.56	-0.59	-0.51	-0.46	-0.56	-0.51	-0.24	0.35	0.53	-0.53
**PSMC3IP**	TG	3.76	0.63	0.70	0.47	0.69	0.64	0.62	0.63	0.73	0.65	0.66	0.60	0.63	0.62	0.57	0.72	0.54	0.51	-0.50	-0.60	-0.58	-0.48	-0.52	-0.54	-0.46	-0.17	0.35	0.51	-0.58
**POLA1**	TG	2.17	0.51	0.57	0.40	0.70	0.67	0.66	0.70	0.68	0.72	0.70	0.54	0.63	0.48	0.43	0.63	0.41	0.40	-0.33	-0.44	-0.43	-0.37	-0.34	-0.48	-0.39	-0.31	0.35	0.43	-0.51
**TACC3**	TG	2.85	0.45	0.51	0.41	0.71	0.62	0.70	0.62	0.73	0.68	0.70	0.61	0.63	0.56	0.41	0.64	0.36	0.48	-0.50	-0.50	-0.62	-0.54	-0.41	-0.53	-0.44	-0.35	0.33	0.49	-0.53
**SGOL1**	TG	2.32	0.64	0.66	0.52	0.70	0.66	0.67	0.65	0.71	0.67	0.70	0.57	0.62	0.65	0.51	0.69	0.50	0.53	-0.50	-0.49	-0.49	-0.50	-0.43	-0.47	-0.43	-0.25	0.34	0.52	-0.59
**MCM4**	TG	3.09	0.54	0.60	0.46	0.69	0.64	0.67	0.64	0.72	0.67	0.70	0.57	0.60	0.59	0.47	0.63	0.47	0.52	-0.44	-0.55	-0.52	-0.51	-0.39	-0.52	-0.45	-0.29	0.39	0.51	-0.58
**ECT2**	TG	4.51	0.56	0.58	0.46	0.70	0.66	0.67	0.67	0.69	0.71	0.71	0.61	0.59	0.59	0.50	0.68	0.47	0.42	-0.36	-0.45	-0.46	-0.37	-0.35	-0.49	-0.45	-0.31	0.35	0.51	-0.52
**GMNN**	DC	3.41	0.63	0.67	0.54	0.71	0.65	0.64	0.63	0.68	0.67	0.70	0.57	0.66	0.66	0.56	0.68	0.52	0.52	-0.49	-0.62	-0.61	-0.53	-0.42	-0.57	-0.45	-0.25	0.33	0.56	-0.61
**FXYD1**	OS	-6.53	-0.67	-0.67	-0.59	-0.56	-0.46	-0.45	-0.46	-0.60	-0.46	-0.51	-0.46	-0.60	-0.63	-0.46	-0.59	-0.45	-0.54	0.71	0.61	0.58	0.56	0.53	0.65	0.51	0.12	-0.26	-0.53	0.74
**TTC36**	TG	-12.57	-0.64	-0.72	-0.56	-0.67	-0.57	-0.58	-0.55	-0.67	-0.57	-0.63	-0.51	-0.68	-0.68	-0.49	-0.62	-0.47	-0.49	0.68	0.70	0.68	0.63	0.60	0.66	0.48	0.20	-0.36	-0.60	0.71
**EZH2**		3.78	0.52	0.58	0.43	0.71	0.64	0.65	0.61	0.71	0.66	0.69	0.59	0.70	0.55	0.53	0.64	0.51	0.51	-0.46	-0.54	-0.57	-0.52	-0.44	-0.56	-0.51	-0.31	0.33	0.49	-0.58
**MCM8**		3.05	0.51	0.60	0.41	0.70	0.63	0.66	0.65	0.72	0.68	0.68	0.59	0.68	0.53	0.48	0.64	0.44	0.48	-0.41	-0.57	-0.56	-0.48	-0.43	-0.60	-0.53	-0.37	0.43	0.44	-0.49
**CKS1B**		2.01	0.61	0.67	0.50	0.73	0.69	0.64	0.67	0.73	0.68	0.70	0.56	0.66	0.60	0.49	0.67	0.44	0.46	-0.40	-0.48	-0.45	-0.46	-0.37	-0.47	-0.39	-0.19	0.37	0.48	-0.59
**MRPS23**		2.26	0.70	0.70	0.54	0.66	0.63	0.60	0.60	0.66	0.63	0.64	0.51	0.61	0.61	0.62	0.70	0.59	0.52	-0.44	-0.54	-0.42	-0.41	-0.46	-0.54	-0.49	-0.17	0.40	0.49	-0.56
**PLOD3**		2.07	0.60	0.59	0.56	0.58	0.48	0.51	0.43	0.61	0.48	0.53	0.60	0.50	0.65	0.57	0.71	0.54	0.55	-0.47	-0.57	-0.51	-0.38	-0.30	-0.52	-0.46	-0.13	0.29	0.71	-0.53
**ATP6V1C1**	TG DC CS OS	2.21	0.73	0.63	0.59	0.60	0.56	0.57	0.48	0.61	0.53	0.57	0.45	0.46	0.62	0.58	0.65	0.59	0.61	-0.51	-0.57	-0.48	-0.36	-0.36	-0.57	-0.50	-0.23	0.38	0.53	-0.59
**GCGR**	TS TG DC CS	-2.79	-0.71	-0.72	-0.57	-0.60	-0.54	-0.50	-0.51	-0.61	-0.51	-0.55	-0.41	-0.54	-0.63	-0.52	-0.64	-0.48	-0.51	0.61	0.57	0.51	0.50	0.43	0.54	0.36	0.06	-0.29	-0.52	0.73
**CHML**	TS TG DC	2.85	0.61	0.59	0.57	0.73	0.64	0.67	0.64	0.69	0.65	0.69	0.60	0.68	0.62	0.42	0.62	0.40	0.52	-0.47	-0.58	-0.52	-0.40	-0.44	-0.47	-0.42	-0.13	0.38	0.54	-0.60
**FERMT1**	TG DC OS	2.82	0.71	0.66	0.64	0.56	0.49	0.50	0.49	0.62	0.50	0.53	0.55	0.51	0.60	0.48	0.63	0.44	0.53	-0.42	-0.48	-0.34	-0.27	-0.35	-0.47	-0.53	-0.05	0.37	0.38	-0.46
**THBS1**	TS TG VI	-2.17	0.07	0.03	0.04	-0.20	-0.17	-0.19	-0.13	-0.14	-0.19	-0.22	-0.13	-0.26	0.13	-0.08	-0.16	-0.03	-0.06	-0.10	0.04	0.18	0.13	-0.10	0.11	0.40	0.77	-0.09	0.15	-0.03
**PRC1**	TG OS	8.49	0.51	0.52	0.41	0.69	0.61	0.64	0.62	0.71	0.66	0.68	0.64	0.65	0.55	0.49	0.65	0.48	0.48	-0.44	-0.54	-0.59	-0.49	-0.37	-0.56	-0.49	-0.32	0.34	0.47	-0.56
**HN1**	TG OS	2.44	0.65	0.74	0.55	0.65	0.59	0.59	0.55	0.66	0.58	0.63	0.62	0.60	0.69	0.56	0.69	0.58	0.56	-0.56	-0.57	-0.53	-0.45	-0.49	-0.54	-0.43	-0.07	0.34	0.62	-0.54
**MAP2**	TG DC	3.04	0.74	0.59	0.70	0.44	0.38	0.38	0.29	0.46	0.35	0.40	0.50	0.36	0.59	0.59	0.57	0.62	0.67	-0.38	-0.44	-0.31	-0.20	-0.28	-0.46	-0.45	-0.06	0.30	0.53	-0.50
**C5orf30**	TG DC	2.11	0.67	0.71	0.62	0.46	0.41	0.42	0.37	0.43	0.41	0.42	0.48	0.35	0.69	0.58	0.60	0.57	0.62	-0.41	-0.46	-0.33	-0.26	-0.37	-0.33	-0.33	-0.02	0.28	0.62	-0.49
**SLITRK6**	TS TC	-25.69	-0.57	-0.48	-0.51	-0.56	-0.48	-0.51	-0.45	-0.59	-0.51	-0.53	-0.60	-0.61	-0.54	-0.53	-0.52	-0.53	-0.56	0.43	0.51	0.51	0.40	0.38	0.50	0.71	0.32	-0.48	-0.40	0.43
**MT3**	TI OS	-7.39	-0.44	-0.53	-0.36	-0.58	-0.48	-0.52	-0.50	-0.57	-0.52	-0.55	-0.49	-0.62	-0.44	-0.41	-0.50	-0.38	-0.45	0.51	0.62	0.67	0.62	0.53	0.73	0.46	0.40	-0.35	-0.27	0.62
**GPC3**	TG	27.21	0.48	0.40	0.44	0.65	0.55	0.58	0.56	0.62	0.60	0.62	0.56	0.88	0.41	0.37	0.51	0.35	0.43	-0.44	-0.50	-0.54	-0.45	-0.30	-0.47	-0.54	-0.36	0.38	0.31	-0.53
**MMS22L**	TG	3.15	0.57	0.63	0.45	0.60	0.53	0.50	0.55	0.62	0.57	0.57	0.59	0.72	0.59	0.47	0.60	0.44	0.46	-0.45	-0.51	-0.52	-0.47	-0.43	-0.53	-0.50	-0.24	0.43	0.45	-0.54
**CENPW**	TG	4.66	0.60	0.68	0.49	0.66	0.57	0.58	0.58	0.69	0.60	0.62	0.60	0.72	0.64	0.51	0.67	0.48	0.52	-0.56	-0.57	-0.61	-0.57	-0.49	-0.64	-0.51	-0.24	0.36	0.54	-0.63
**HIST2H2BF**	TG	4.51	0.52	0.56	0.45	0.69	0.59	0.64	0.60	0.72	0.63	0.67	0.61	0.70	0.60	0.43	0.63	0.39	0.46	-0.55	-0.53	-0.64	-0.53	-0.45	-0.59	-0.43	-0.31	0.34	0.47	-0.59
**CCNA2**	TG	10.29	0.57	0.58	0.50	0.70	0.61	0.64	0.58	0.72	0.64	0.68	0.62	0.69	0.60	0.53	0.69	0.48	0.52	-0.52	-0.59	-0.66	-0.55	-0.41	-0.60	-0.52	-0.37	0.36	0.52	-0.58
**KIF4A**	TG	15.38	0.61	0.65	0.49	0.69	0.63	0.63	0.60	0.72	0.65	0.68	0.61	0.69	0.63	0.54	0.66	0.53	0.54	-0.54	-0.60	-0.61	-0.56	-0.47	-0.63	-0.54	-0.36	0.39	0.49	-0.61
**ESCO2**	TG	5.60	0.54	0.60	0.47	0.70	0.64	0.65	0.59	0.72	0.65	0.68	0.62	0.68	0.61	0.51	0.68	0.50	0.51	-0.45	-0.53	-0.57	-0.46	-0.40	-0.51	-0.49	-0.29	0.40	0.52	-0.51
**ORC6**	TG	5.40	0.61	0.63	0.52	0.68	0.60	0.63	0.61	0.71	0.64	0.66	0.58	0.68	0.66	0.48	0.67	0.46	0.53	-0.59	-0.62	-0.64	-0.60	-0.47	-0.59	-0.46	-0.26	0.40	0.58	-0.59
**SGOL2**	TG	3.54	0.60	0.69	0.47	0.67	0.59	0.57	0.59	0.71	0.62	0.64	0.55	0.68	0.63	0.49	0.63	0.48	0.50	-0.56	-0.61	-0.60	-0.60	-0.48	-0.67	-0.53	-0.32	0.40	0.43	-0.67
**NEIL3**	TG	6.99	0.68	0.69	0.59	0.67	0.60	0.63	0.58	0.69	0.63	0.65	0.62	0.67	0.64	0.60	0.73	0.56	0.63	-0.52	-0.56	-0.61	-0.49	-0.49	-0.59	-0.53	-0.31	0.48	0.55	-0.62
**NCAPG**	TG	13.86	0.62	0.62	0.52	0.68	0.59	0.62	0.56	0.71	0.61	0.66	0.61	0.67	0.63	0.55	0.68	0.54	0.59	-0.56	-0.60	-0.63	-0.55	-0.46	-0.62	-0.56	-0.34	0.38	0.53	-0.62
**KIF20A**	TG	16.37	0.59	0.58	0.49	0.70	0.62	0.65	0.60	0.71	0.65	0.69	0.62	0.67	0.61	0.56	0.69	0.55	0.54	-0.50	-0.55	-0.60	-0.50	-0.38	-0.62	-0.54	-0.38	0.38	0.50	-0.60
**NDC80**	TG	6.90	0.57	0.61	0.48	0.70	0.61	0.62	0.56	0.71	0.62	0.67	0.59	0.67	0.60	0.51	0.69	0.48	0.48	-0.44	-0.57	-0.59	-0.50	-0.38	-0.58	-0.53	-0.35	0.42	0.54	-0.54
**OIP5**	TG	9.15	0.56	0.60	0.45	0.69	0.63	0.63	0.63	0.71	0.67	0.68	0.60	0.66	0.61	0.49	0.66	0.46	0.49	-0.47	-0.57	-0.59	-0.55	-0.43	-0.62	-0.53	-0.37	0.38	0.49	-0.60
**TRIP13**	TG	7.22	0.58	0.61	0.49	0.69	0.60	0.65	0.60	0.71	0.64	0.67	0.61	0.65	0.63	0.49	0.68	0.48	0.52	-0.58	-0.60	-0.64	-0.56	-0.46	-0.63	-0.52	-0.30	0.39	0.55	-0.62
**CKAP2L**	TG	4.05	0.58	0.59	0.50	0.70	0.62	0.64	0.63	0.71	0.68	0.69	0.62	0.65	0.59	0.51	0.64	0.48	0.50	-0.46	-0.49	-0.56	-0.48	-0.37	-0.55	-0.51	-0.34	0.40	0.53	-0.53
**KIF23**	TG	9.26	0.52	0.57	0.42	0.69	0.63	0.66	0.64	0.72	0.68	0.68	0.62	0.65	0.58	0.47	0.64	0.46	0.47	-0.49	-0.54	-0.59	-0.52	-0.46	-0.62	-0.46	-0.30	0.35	0.50	-0.59
**NDRG3**	TG	2.32	0.61	0.67	0.50	0.69	0.65	0.64	0.68	0.68	0.69	0.68	0.63	0.64	0.64	0.52	0.72	0.53	0.46	-0.43	-0.51	-0.51	-0.40	-0.29	-0.58	-0.51	-0.29	0.32	0.51	-0.54
**NUSAP1**	TG	4.96	0.49	0.55	0.38	0.67	0.61	0.62	0.62	0.70	0.65	0.66	0.57	0.64	0.55	0.41	0.62	0.39	0.43	-0.48	-0.55	-0.61	-0.56	-0.43	-0.62	-0.46	-0.38	0.33	0.42	-0.60
**ANKRD27**	TG	2.18	0.65	0.69	0.49	0.67	0.61	0.57	0.58	0.67	0.61	0.63	0.55	0.64	0.63	0.55	0.73	0.52	0.52	-0.44	-0.57	-0.51	-0.44	-0.33	-0.58	-0.46	-0.20	0.35	0.51	-0.62
**UHRF1**	TG	4.19	0.50	0.56	0.38	0.66	0.61	0.62	0.63	0.70	0.65	0.66	0.60	0.63	0.57	0.45	0.61	0.44	0.42	-0.46	-0.51	-0.54	-0.50	-0.46	-0.53	-0.40	-0.24	0.31	0.42	-0.56
**KIFC1**	TG	6.42	0.51	0.58	0.41	0.68	0.59	0.63	0.59	0.70	0.63	0.67	0.60	0.63	0.56	0.48	0.64	0.48	0.50	-0.53	-0.59	-0.65	-0.56	-0.45	-0.64	-0.48	-0.34	0.27	0.46	-0.61
**CENPH**	TG	2.82	0.60	0.64	0.48	0.68	0.63	0.62	0.63	0.70	0.65	0.66	0.54	0.62	0.64	0.53	0.64	0.48	0.48	-0.47	-0.54	-0.52	-0.46	-0.42	-0.52	-0.47	-0.21	0.37	0.53	-0.56
**FEN1**	TG	3.73	0.59	0.60	0.44	0.68	0.65	0.65	0.66	0.71	0.69	0.68	0.57	0.60	0.56	0.53	0.65	0.53	0.49	-0.39	-0.52	-0.49	-0.43	-0.38	-0.58	-0.45	-0.24	0.43	0.44	-0.55
**C11orf82**	TG	3.42	0.56	0.55	0.50	0.68	0.59	0.64	0.59	0.70	0.64	0.66	0.65	0.60	0.64	0.52	0.69	0.46	0.54	-0.47	-0.50	-0.56	-0.45	-0.36	-0.53	-0.51	-0.34	0.36	0.54	-0.50
**TPX2**	TG	3.89	0.47	0.51	0.35	0.68	0.63	0.66	0.64	0.71	0.69	0.68	0.58	0.60	0.46	0.39	0.60	0.39	0.38	-0.42	-0.48	-0.53	-0.47	-0.37	-0.54	-0.43	-0.37	0.33	0.40	-0.53
**TK1**	TG	4.01	0.57	0.55	0.49	0.68	0.62	0.66	0.60	0.69	0.65	0.68	0.62	0.59	0.54	0.53	0.71	0.50	0.55	-0.41	-0.46	-0.52	-0.44	-0.32	-0.57	-0.53	-0.38	0.38	0.46	-0.50
**COA6**	OS	2.26	0.59	0.62	0.47	0.65	0.58	0.55	0.54	0.62	0.56	0.63	0.49	0.71	0.48	0.53	0.65	0.51	0.53	-0.42	-0.53	-0.52	-0.45	-0.42	-0.64	-0.55	-0.33	0.35	0.36	-0.56
**CENPP**	DC	2.69	0.58	0.58	0.42	0.67	0.63	0.60	0.60	0.65	0.63	0.63	0.59	0.56	0.55	0.55	0.74	0.51	0.45	-0.41	-0.52	-0.54	-0.37	-0.29	-0.54	-0.46	-0.23	0.34	0.47	-0.51
**DCAF13**	DC	2.15	0.72	0.62	0.61	0.67	0.64	0.64	0.58	0.65	0.62	0.66	0.50	0.46	0.61	0.60	0.66	0.58	0.60	-0.36	-0.48	-0.34	-0.28	-0.32	-0.43	-0.44	-0.14	0.31	0.56	-0.51
**ANXA2**	TG	2.40	0.64	0.64	0.55	0.46	0.40	0.39	0.40	0.51	0.42	0.42	0.54	0.41	0.72	0.50	0.52	0.53	0.53	-0.58	-0.53	-0.45	-0.37	-0.54	-0.52	-0.38	0.16	0.26	0.65	-0.50
**FGFR2**	TC	-3.67	-0.44	-0.29	-0.38	-0.30	-0.22	-0.21	-0.14	-0.33	-0.19	-0.27	-0.36	-0.41	-0.26	-0.41	-0.36	-0.41	-0.48	0.16	0.20	0.13	0.03	0.04	0.26	0.85	0.28	-0.34	-0.14	0.15
**SFRP5**	TC	-150.76	-0.52	-0.40	-0.43	-0.34	-0.27	-0.27	-0.22	-0.36	-0.26	-0.30	-0.41	-0.46	-0.37	-0.54	-0.45	-0.54	-0.54	0.31	0.43	0.41	0.34	0.28	0.48	0.73	0.44	-0.42	-0.24	0.39
**MT1F**	TC	-17.25	-0.51	-0.50	-0.40	-0.56	-0.47	-0.50	-0.47	-0.57	-0.50	-0.54	-0.49	-0.57	-0.44	-0.48	-0.54	-0.47	-0.53	0.49	0.56	0.57	0.55	0.45	0.70	0.56	0.37	-0.34	-0.32	0.61
**MT1E**	TI	-8.22	-0.44	-0.50	-0.33	-0.57	-0.50	-0.52	-0.51	-0.55	-0.54	-0.55	-0.42	-0.60	-0.42	-0.39	-0.49	-0.36	-0.42	0.45	0.57	0.60	0.58	0.45	0.71	0.45	0.42	-0.34	-0.24	0.64
**MT1M**	TI	-5.78	-0.44	-0.51	-0.34	-0.56	-0.46	-0.50	-0.49	-0.56	-0.50	-0.53	-0.47	-0.61	-0.44	-0.39	-0.47	-0.36	-0.45	0.53	0.63	0.66	0.64	0.53	0.74	0.50	0.41	-0.34	-0.26	0.63

**MT4**	TI	-9.30	-0.46	-0.55	-0.38	-0.60	-0.51	-0.54	-0.52	-0.59	-0.55	-0.58	-0.50	-0.64	-0.47	-0.43	-0.54	-0.39	-0.48	0.51	0.62	0.66	0.63	0.51	0.71	0.48	0.42	-0.36	-0.30	0.63
**CXCL14**	TC	-114.01	-0.55	-0.46	-0.47	-0.57	-0.48	-0.51	-0.44	-0.59	-0.50	-0.55	-0.57	-0.65	-0.51	-0.50	-0.53	-0.49	-0.56	0.45	0.54	0.57	0.46	0.34	0.57	0.73	0.42	-0.43	-0.34	0.50
**FCN3**	TC	-20.42	-0.58	-0.53	-0.49	-0.64	-0.54	-0.57	-0.52	-0.66	-0.56	-0.62	-0.61	-0.68	-0.58	-0.51	-0.57	-0.49	-0.53	0.52	0.58	0.61	0.52	0.42	0.62	0.72	0.36	-0.46	-0.43	0.56
**SMYD3**		2.77	0.47	0.40	0.38	0.67	0.57	0.60	0.59	0.65	0.58	0.64	0.50	0.70	0.39	0.29	0.49	0.26	0.29	-0.39	-0.55	-0.53	-0.45	-0.35	-0.52	-0.45	-0.21	0.41	0.31	-0.51
**EHMT2**		2.13	0.57	0.56	0.54	0.67	0.56	0.61	0.55	0.71	0.59	0.65	0.64	0.69	0.56	0.48	0.61	0.42	0.52	-0.50	-0.66	-0.61	-0.56	-0.32	-0.59	-0.50	-0.36	0.37	0.42	-0.61
**HIST1H2BJ**		2.69	0.58	0.63	0.49	0.70	0.61	0.63	0.60	0.71	0.62	0.67	0.51	0.66	0.60	0.46	0.66	0.42	0.45	-0.58	-0.62	-0.66	-0.57	-0.48	-0.64	-0.45	-0.29	0.36	0.51	-0.65
**PEA15**		2.35	0.56	0.62	0.46	0.69	0.59	0.60	0.57	0.66	0.60	0.63	0.64	0.65	0.56	0.53	0.70	0.52	0.48	-0.47	-0.53	-0.61	-0.45	-0.41	-0.63	-0.46	-0.15	0.32	0.45	-0.57
**ALYREF**		2.72	0.61	0.62	0.54	0.68	0.60	0.63	0.61	0.72	0.63	0.65	0.67	0.65	0.58	0.51	0.66	0.48	0.52	-0.48	-0.59	-0.54	-0.45	-0.49	-0.60	-0.52	-0.16	0.38	0.50	-0.51
**MND1**		5.88	0.58	0.56	0.47	0.69	0.61	0.62	0.60	0.70	0.64	0.66	0.56	0.64	0.56	0.51	0.64	0.49	0.49	-0.47	-0.56	-0.56	-0.48	-0.43	-0.56	-0.50	-0.34	0.40	0.49	-0.61
**CNIH4**		2.13	0.58	0.59	0.46	0.72	0.66	0.66	0.64	0.68	0.65	0.70	0.54	0.64	0.59	0.45	0.64	0.45	0.46	-0.41	-0.51	-0.47	-0.39	-0.39	-0.54	-0.45	-0.23	0.35	0.45	-0.57
**SCNM1**		2.38	0.61	0.59	0.53	0.69	0.63	0.64	0.57	0.70	0.62	0.68	0.61	0.60	0.57	0.55	0.65	0.55	0.55	-0.46	-0.60	-0.56	-0.47	-0.39	-0.66	-0.56	-0.39	0.40	0.48	-0.59
**NME1**		2.19	0.63	0.63	0.52	0.61	0.55	0.58	0.52	0.64	0.55	0.60	0.50	0.56	0.55	0.59	0.71	0.58	0.56	-0.49	-0.55	-0.53	-0.47	-0.39	-0.62	-0.50	-0.18	0.35	0.52	-0.49
**PRKDC**		2.06	0.59	0.51	0.55	0.67	0.61	0.66	0.58	0.71	0.63	0.68	0.56	0.50	0.53	0.48	0.56	0.45	0.52	-0.36	-0.49	-0.41	-0.34	-0.30	-0.45	-0.51	-0.31	0.37	0.53	-0.48
**SQLE**		3.79	0.43	0.38	0.39	0.43	0.38	0.42	0.35	0.48	0.40	0.41	0.49	0.48	0.37	0.33	0.36	0.37	0.39	-0.29	-0.35	-0.39	-0.30	-0.28	-0.44	-0.53	-0.28	0.73	0.32	-0.24
**HHIPL2**		7.35	0.67	0.51	0.69	0.43	0.31	0.36	0.27	0.51	0.32	0.39	0.64	0.47	0.52	0.54	0.53	0.46	0.71	-0.44	-0.47	-0.44	-0.31	-0.37	-0.52	-0.55	-0.16	0.38	0.39	-0.51
**DUSP1**		-2.42	-0.44	-0.53	-0.39	-0.48	-0.41	-0.45	-0.38	-0.51	-0.40	-0.46	-0.41	-0.49	-0.39	-0.37	-0.46	-0.34	-0.41	0.41	0.44	0.44	0.45	0.71	0.50	0.40	0.24	-0.23	-0.30	0.38
**AK316321**		-2.67	-0.42	-0.52	-0.36	-0.46	-0.39	-0.43	-0.36	-0.50	-0.38	-0.44	-0.40	-0.49	-0.37	-0.35	-0.45	-0.33	-0.42	0.44	0.45	0.47	0.48	0.71	0.53	0.41	0.30	-0.24	-0.30	0.40
**SAMD11**		-4.75	-0.59	-0.43	-0.50	-0.42	-0.36	-0.39	-0.33	-0.50	-0.37	-0.41	-0.48	-0.50	-0.48	-0.47	-0.46	-0.47	-0.55	0.47	0.45	0.42	0.36	0.33	0.45	0.73	0.35	-0.42	-0.27	0.38
**ACADS**		-2.82	-0.56	-0.62	-0.51	-0.46	-0.35	-0.37	-0.31	-0.51	-0.33	-0.39	-0.40	-0.52	-0.64	-0.42	-0.48	-0.40	-0.50	0.72	0.59	0.62	0.56	0.61	0.57	0.46	0.06	-0.20	-0.53	0.58
**SLC7A2**		-4.30	-0.59	-0.54	-0.54	-0.59	-0.46	-0.54	-0.46	-0.63	-0.51	-0.54	-0.60	-0.53	-0.52	-0.53	-0.69	-0.46	-0.45	0.51	0.54	0.60	0.41	0.42	0.77	0.55	0.20	-0.35	-0.52	0.54
**ACAT1**		-2.28	-0.50	-0.58	-0.49	-0.46	-0.35	-0.38	-0.38	-0.50	-0.39	-0.41	-0.42	-0.53	-0.59	-0.38	-0.52	-0.35	-0.45	0.71	0.56	0.61	0.62	0.54	0.61	0.45	0.13	-0.23	-0.47	0.56
**TGM5**		-2.07	-0.39	-0.51	-0.38	-0.62	-0.50	-0.55	-0.55	-0.59	-0.56	-0.57	-0.46	-0.59	-0.57	-0.30	-0.51	-0.24	-0.36	0.60	0.57	0.70	0.58	0.52	0.56	0.32	0.16	-0.11	-0.52	0.58
**STAB2**		-27.82	-0.57	-0.50	-0.51	-0.57	-0.49	-0.51	-0.44	-0.58	-0.50	-0.54	-0.59	-0.63	-0.60	-0.52	-0.55	-0.52	-0.56	0.49	0.55	0.56	0.45	0.39	0.60	0.70	0.34	-0.44	-0.46	0.53
**TNFRSF13B**		-3.00	-0.46	-0.48	-0.38	-0.56	-0.47	-0.51	-0.49	-0.60	-0.51	-0.54	-0.47	-0.63	-0.48	-0.44	-0.51	-0.43	-0.44	0.56	0.63	0.62	0.59	0.48	0.75	0.61	0.38	-0.34	-0.32	0.60
**GLS2**		-3.48	-0.59	-0.62	-0.51	-0.63	-0.52	-0.56	-0.52	-0.66	-0.55	-0.59	-0.58	-0.63	-0.54	-0.48	-0.62	-0.46	-0.46	0.53	0.53	0.57	0.45	0.51	0.71	0.59	0.19	-0.37	-0.49	0.60
**TRIM56**		-2.58	-0.54	-0.50	-0.43	-0.58	-0.48	-0.53	-0.50	-0.63	-0.52	-0.54	-0.56	-0.64	-0.52	-0.46	-0.50	-0.45	-0.47	0.59	0.59	0.61	0.57	0.53	0.71	0.55	0.27	-0.45	-0.37	0.65
**BAI3**		-5.06	-0.62	-0.53	-0.56	-0.57	-0.49	-0.51	-0.47	-0.62	-0.52	-0.56	-0.53	-0.67	-0.53	-0.52	-0.57	-0.50	-0.61	0.51	0.50	0.55	0.48	0.41	0.57	0.71	0.42	-0.44	-0.44	0.53
**C1R**		-2.79	-0.49	-0.57	-0.40	-0.62	-0.56	-0.53	-0.59	-0.61	-0.60	-0.61	-0.42	-0.70	-0.55	-0.43	-0.54	-0.39	-0.41	0.59	0.56	0.54	0.72	0.44	0.53	0.41	0.26	-0.23	-0.41	0.62
**Total circRNA-mRNA pairs (r>0.7)**		**460**	**9**	**6**	**3**	**95**	**6**	**10**	**6**	**121**	**25**	**66**	**1**	**33**	**1**	**2**	**31**	**2**	**5**	**4**	**1**	**1**	**1**	**2**	**11**	**11**	**1**	**1**	**1**	**4**

FOOTNOTE: TS: Tumor size, TG: Tumor Grade, TC: Tumor Capsule, DC: Degree of encapsulation, VI: Vascular invasion, TI: Tumor Invasion, CS: Cancer stage, OS: Overall Survival. Red outline: Upregulate in worse clinical features. Green outline: Upregulate in better clinical features.

Table S4. Genes commonly deregulated by all 5 key/nodal circRNAs.

No.	GeneName	LO2- circW3 overexpression		LO2- circW7 overexpression		Huh7- circW8 knockdown		LO2- circRaly overexpression		LO2- circGPC3 overexpression	
		FDR	FC	FDR	FC	FDR	FC	FDR	FC	FDR	FC
1	PRKACA	1.01E-314	8800.50	0.00E+00	11357.26	4.36E-148	-5.14	5.14E-310	8765.98	8.57E-111	3210.65
2	RAB5B	1.33E-240	6666.27	3.36E-267	7449.11	1.06E-226	-6456.73	5.17E-73	1997.49	3.41E-48	1376.30
3	PARP6	7.01E-130	3549.24	1.19E-57	1555.90	1.69E-47	-1304.20	5.10E-30	804.29	3.29E-64	1843.34
4	TWNK	8.06E-108	2935.40	1.18E-174	4816.64	1.52E-15	-2.29	4.81E-171	4756.11	4.64E-197	5779.85
5	ADGRL1	2.12E-87	2369.28	3.94E-68	1844.89	1.14E-15	-2.11	3.82E-93	2558.67	6.08E-09	233.50
6	THUMPD2	1.60E-77	2096.95	6.60E-28	738.42	1.38E-20	-546.82	7.45E-32	855.02	1.16E-62	1798.41
7	CASP8AP2	7.89E-65	1747.17	1.15E-07	182.05	7.56E-13	-4.35	1.66E-06	151.89	3.77E-40	1142.03
8	GLYR1(NR_136697)	7.46E-60	1610.44	9.10E-31	816.81	3.36E-18	-479.76	9.66E-59	1600.92	6.88E-52	1484.28
9	AMPD2(NM_004037)	1.05E-58	1578.89	1.68E-54	1469.14	7.74E-54	-2.15	5.73E-88	2413.86	6.67E-118	3420.96
10	ZNF197	4.57E-52	1396.58	5.03E-15	384.83	1.55E-07	-2.52	1.81E-28	761.23	1.62E-15	425.44
11	MEF2A	3.71E-51	1371.69	5.25E-27	713.64	4.69E-41	-1122.66	2.05E-47	1286.19	4.58E-36	1023.18
12	TMEM135	3.43E-50	1345.17	1.04E-12	321.16	5.59E-06	-2.13	7.88E-12	300.37	1.53E-08	221.71
13	CNTROB	5.77E-50	1338.98	1.12E-86	2357.47	2.36E-19	-2.34	1.32E-59	1624.84	8.23E-56	1598.55
14	CNOT4	4.06E-47	1260.82	2.28E-78	2127.38	1.28E-11	-3.00	9.05E-27	714.24	9.77E-77	2210.26
15	SMARCA2	2.75E-45	1210.63	2.36E-26	695.81	1.60E-16	-432.57	8.66E-17	438.10	1.50E-18	513.87
16	ZFAND1	1.77E-42	1133.70	9.84E-62	1668.64	1.40E-14	-2.16	2.11E-47	1285.83	1.06E-19	547.40
17	PCGF5	1.00E-31	839.44	6.75E-20	518.56	1.69E-10	-263.13	1.11E-16	435.09	2.29E-22	624.73
18	CXorf38	1.70E-30	805.69	1.61E-22	590.61	7.44E-08	-3.67	5.39E-35	942.03	4.43E-24	674.63
19	RGS14	6.15E-29	763.11	4.63E-43	1154.54	4.04E-12	-308.65	4.16E-42	1139.04	1.12E-32	924.50
20	ZCCHC8	4.56E-28	739.32	5.18E-15	384.48	3.19E-17	-452.30	2.05E-14	372.25	1.51E-13	367.97
21	LPP	8.80E-27	704.12	2.36E-06	145.83	1.95E-34	-936.44	7.95E-19	494.56	1.76E-30	860.59
22	DCAF5	5.90E-25	654.14	9.19E-26	679.61	6.05E-13	-2.25	1.38E-27	736.90	1.47E-23	659.50
23	DERL1	2.27E-24	638.14	1.75E-08	204.76	1.63E-15	-9.70	4.90E-26	693.95	3.22E-19	533.38
24	VEZT	2.50E-22	582.16	5.52E-12	301.24	1.73E-29	-797.32	1.18E-07	183.96	5.81E-08	204.82
25	MFSD11(NM_001242533)	1.18E-19	509.02	4.82E-21	550.03	2.04E-10	-260.84	3.69E-17	448.30	2.19E-30	857.86
26	SLC25A45(NM_001352381)	1.91E-19	503.29	3.17E-16	417.88	4.74E-17	-447.44	1.06E-35	961.34	1.36E-15	427.69
27	MSTO1	2.36E-18	473.41	1.02E-06	155.91	3.63E-13	-4.70	7.47E-08	189.51	5.59E-08	205.34
28	PPP2R2B	3.07E-18	470.29	1.97E-25	670.47	2.44E-13	-342.99	4.36E-22	584.69	1.06E-07	197.08
29	ILRUN	1.28E-17	453.27	7.77E-17	434.64	3.38E-36	-17.19	1.74E-10	262.93	1.25E-16	457.92
30	C2orf42(NM_017880)	1.38E-16	425.01	3.76E-39	1047.01	6.72E-09	-3.92	7.09E-34	911.01	3.50E-31	881.05
31	DACT2(NR_104425)	2.27E-15	391.63	7.40E-15	380.20	9.70E-14	-2.51	6.64E-26	690.29	9.20E-14	374.28
32	PSME3	3.28E-15	387.22	7.17E-11	270.49	1.99E-04	-2.44	8.50E-13	327.39	2.51E-13	361.55
33	UPF3A	1.85E-14	366.72	7.31E-13	325.43	5.40E-10	-248.92	3.19E-13	339.13	3.32E-09	241.18
34	RALGDS	3.31E-14	359.75	3.16E-28	747.19	8.82E-17	-2.13	4.15E-40	1083.65	2.89E-37	1058.14
35	BRD8	2.32E-13	336.61	3.60E-13	333.85	8.31E-05	-2.35	1.70E-12	318.95	6.42E-10	262.10
36	ASCC2	5.97E-13	325.37	1.43E-28	756.49	6.41E-08	-5.42	7.40E-38	1021.28	1.85E-26	743.99
37	DBN1	1.08E-12	318.29	1.80E-41	1110.83	6.48E-18	-8.11	1.39E-46	1263.19	1.28E-19	545.01
38	PSPH	5.14E-12	299.79	2.11E-07	174.77	3.54E-13	-338.45	2.29E-08	203.82	2.31E-18	508.44
39	SPAG1	6.52E-11	269.47	2.01E-38	1027.02	1.35E-36	-997.23	2.34E-10	259.36	2.98E-09	242.52
40	STIMATE-MUSTN1	8.25E-11	266.63	5.54E-12	301.19	4.20E-09	-5.98	2.92E-17	451.13	2.40E-10	274.58
41	BIVM	8.95E-11	265.65	2.63E-07	172.19	2.25E-28	-766.01	8.91E-11	271.08	3.85E-13	356.19
42	STOM	9.07E-11	265.49	7.62E-20	517.12	5.41E-04	-4.51	7.08E-11	273.86	3.40E-07	182.04
43	GOSR2	3.29E-10	250.12	2.41E-11	283.52	3.07E-02	-2.34	1.80E-06	150.88	1.02E-08	226.87
44	AMZ2	4.51E-10	246.36	1.16E-20	539.57	5.92E-11	-275.94	2.94E-12	312.31	5.06E-10	265.10
45	ACBD4	1.01E-09	236.69	4.50E-25	660.65	5.56E-10	-3.28	3.56E-20	531.84	1.46E-18	514.24
46	DMTN(NM_001323384)	1.17E-09	234.91	6.73E-40	1067.57	5.57E-58	-1600.21	1.07E-20	546.23	1.11E-17	488.66
47	ABLIM2	3.80E-09	220.85	8.63E-20	515.63	9.82E-03	-2.44	6.46E-13	330.71	1.78E-07	190.33
48	GDPGP1	5.17E-09	217.16	1.01E-08	211.28	6.15E-11	-4.65	2.82E-06	145.41	2.11E-13	363.78
49	MAP3K13	1.52E-07	176.73	6.93E-14	353.50	6.22E-14	-3.55	1.83E-08	206.55	3.09E-13	359.00
50	ITGA7	2.10E-07	172.87	6.16E-11	272.30	6.96E-29	-780.32	2.92E-06	144.98	4.92E-09	236.20
51	QPCTL	5.30E-07	161.80	1.13E-13	347.70	1.19E-05	-2.39	1.06E-11	296.83	4.64E-19	528.76
52	SEC24B(NM_001318085)	7.16E-07	158.20	3.67E-21	553.30	6.99E-29	-780.25	4.10E-22	585.45	1.10E-20	575.95
53	TRIM16	2.49E-06	143.24	1.71E-06	149.71	4.21E-13	-336.33	4.71E-14	362.17	1.16E-15	429.69
54	INTS7	2.69E-06	142.34	9.00E-08	185.04	1.01E-10	-269.37	1.19E-08	211.74	1.54E-12	338.54
55	AP1G2	3.80E-06	138.21	2.75E-10	254.40	7.22E-15	-3.50	1.28E-13	350.19	1.00E-06	168.23
56	PLA2G15	1.29E-24	18.29	5.69E-04	4.41	3.43E-03	-2.56	1.22E-08	7.82	3.88E-04	4.46
57	C1orf43	4.33E-16	14.84	6.02E-10	10.01	2.46E-05	-2.07	9.24E-16	14.53	9.87E-08	7.94
58	SNX13	6.93E-37	14.66	3.51E-28	11.85	9.25E-03	-2.25	6.08E-11	5.93	2.89E-08	4.80
59	NUB1	6.52E-27	12.57	4.32E-27	12.64	3.58E-13	-2.49	8.19E-08	5.09	1.02E-14	7.68
60	TXNDC11	3.85E-15	9.97	4.66E-03	3.20	1.42E-08	-2.19	1.42E-18	11.63	4.92E-04	3.79

61	MLEC	3.89E-09	9.14	4.69E-07	7.47	3.32E-05	-3.29	2.67E-04	5.09	2.76E-04	5.00
62	MIOS	2.29E-11	8.80	1.10E-10	8.41	1.99E-12	-2.23	2.14E-20	13.91	4.00E-04	4.12
63	DCUN1D4	4.51E-12	8.32	5.43E-04	3.80	9.64E-08	-2.79	1.32E-03	3.54	1.53E-03	3.46
64	ADAR	7.58E-44	7.87	4.00E-35	6.77	2.33E-16	-428.04	7.90E-55	9.14	3.20E-22	4.90
65	SNX6	9.55E-17	6.71	5.73E-12	5.37	8.98E-05	-2.47	4.75E-06	3.51	8.40E-13	5.42
66	LDLR	7.07E-21	6.38	2.66E-15	5.23	4.79E-04	-2.05	3.18E-12	4.53	1.07E-02	2.03
67	NFIA	2.99E-11	5.81	4.44E-04	3.08	1.06E-14	-381.27	8.71E-18	7.95	2.22E-05	3.55
68	REC8	8.86E-25	5.53	1.34E-11	3.57	1.40E-08	-209.17	8.94E-08	2.90	1.63E-10	3.30
69	ITSN1	1.75E-21	5.13	1.20E-03	2.08	1.93E-09	-2.56	8.91E-04	2.10	1.08E-09	3.20
70	CNOT7	6.92E-09	5.09	2.73E-10	5.63	1.21E-22	-604.64	8.84E-05	3.44	4.29E-16	7.40
71	R3HDM1	3.58E-17	5.08	2.58E-34	7.99	3.54E-13	-2.11	8.81E-10	3.65	1.73E-20	5.47
72	ZSCAN32	1.29E-06	5.04	8.10E-13	8.13	1.21E-08	-5.50	1.14E-04	3.99	5.53E-05	4.10
73	FNDC3A	1.77E-44	4.82	2.19E-21	3.27	2.06E-04	-3.90	1.36E-06	2.00	2.16E-07	2.05
74	LRRFIP1	2.31E-19	4.65	1.32E-11	3.50	1.13E-13	-4.19	2.20E-14	3.91	8.61E-21	4.69
75	STAT6	3.51E-16	4.60	1.31E-48	9.47	6.10E-05	-2.51	3.86E-09	3.33	1.92E-09	3.31
76	ABTB1	1.02E-08	4.59	5.61E-03	2.46	1.97E-13	-345.61	2.13E-17	7.23	2.88E-20	7.80
77	TP53I13	5.92E-11	4.43	1.49E-09	4.09	1.25E-04	-4.47	5.39E-03	2.20	3.41E-03	2.25
78	PNISR	2.03E-50	4.30	3.06E-27	3.15	3.92E-32	-3.93	8.04E-36	3.57	2.38E-20	2.69
79	CXCL10	2.90E-39	4.29	3.39E-24	3.34	9.73E-04	-2.10	1.90E-27	3.54	5.14E-11	2.31
80	RSRP1	1.12E-06	4.26	1.98E-07	4.58	1.05E-10	-13.03	3.38E-02	2.20	4.61E-06	3.92
81	IKZF3	2.81E-04	4.10	1.46E-09	7.32	2.32E-14	-371.74	9.84E-05	4.40	8.46E-05	4.37
82	TLE4	2.01E-09	3.98	2.52E-36	9.63	6.07E-05	-3.54	1.46E-20	6.43	4.20E-60	13.53
83	SF1	1.57E-20	3.91	5.29E-08	2.48	7.68E-11	-2.35	1.27E-18	3.69	1.94E-34	5.06
84	SMAD7	5.41E-09	3.87	3.07E-10	4.19	7.56E-18	-5.53	4.30E-04	2.53	1.58E-09	3.90
85	STAT3	1.08E-28	3.83	1.90E-42	4.75	8.70E-66	-1820.81	8.54E-54	5.42	6.35E-66	5.96
86	SLC9A1	6.08E-36	3.78	3.83E-59	4.96	9.86E-15	-382.21	1.73E-40	4.00	1.26E-44	4.09
87	LYPLA1	6.73E-05	3.74	3.25E-14	7.71	2.06E-21	-3.77	3.76E-14	7.64	3.22E-24	11.04
88	DENND1A	2.41E-03	3.60	3.44E-02	2.70	9.28E-12	-298.53	6.20E-12	9.11	2.20E-11	8.53
89	UGT8	1.73E-06	3.37	5.14E-05	2.94	1.65E-02	-2.01	7.57E-07	3.47	1.36E-07	3.60
90	H2BC12	3.58E-06	3.09	1.47E-02	2.00	1.36E-07	-3.35	5.82E-03	2.14	9.49E-03	2.05
91	URGCP	2.50E-02	2.98	5.73E-07	6.74	1.93E-04	-2.19	1.85E-10	9.18	1.21E-05	5.61
92	RAF1	1.77E-03	2.93	9.40E-35	14.53	6.58E-10	-246.49	5.06E-51	19.76	2.96E-04	3.28
93	SIRT2	3.41E-03	2.91	2.60E-15	8.34	3.58E-25	-5.68	1.02E-10	6.42	3.71E-03	2.87
94	ZNF266(NM_006631)	1.17E-05	2.88	3.68E-22	6.44	2.77E-02	-2.27	1.48E-05	2.85	3.90E-03	2.14
95	ETNPPL	2.74E-06	2.86	2.52E-08	3.32	3.09E-18	-6.04	3.04E-08	3.28	1.79E-05	2.62
96	ARHGAP17	4.52E-11	2.68	3.43E-19	3.47	7.50E-07	-2.21	1.27E-05	2.03	5.38E-07	2.17
97	SECTM1	2.27E-74	2.56	9.47E-74	2.55	3.53E-09	-2.43	1.24E-96	2.83	2.76E-51	2.18
98	TMEM263	3.75E-02	2.19	6.27E-09	5.28	2.22E-04	-3.33	1.74E-06	4.26	1.57E-03	2.91
1	IARS1	1.62E-310	-8506.50	2.28E-97	-3.69	1.00E-16	409.66	4.07E-71	-2.82	8.55E-156	-5.60
2	AMPD2(NM_001257361)	1.51E-100	-2682.39	4.79E-101	-2694.38	1.31E-18	459.15	1.80E-103	-2741.88	6.62E-116	-3000.28
3	TSC2	6.10E-77	-2042.11	3.26E-32	-4.90	1.34E-87	2276.95	3.75E-79	-2087.39	1.45E-88	-2284.09
4	GAPVD1	1.36E-71	-1897.58	6.24E-72	-1906.05	1.63E-63	1640.40	1.25E-73	-1939.65	2.17E-82	-2122.43
5	ZC3H11A	3.89E-60	-1588.11	2.14E-60	-1595.21	1.88E-31	796.58	8.37E-62	-1623.32	3.84E-69	-1776.27
6	ZNF778	1.87E-59	-1569.76	1.02E-59	-1576.77	2.84E-52	1343.96	4.21E-61	-1604.57	2.32E-68	-1755.75
7	ZFYVE16	9.26E-59	-1551.08	5.13E-59	-1558.00	1.02E-71	1857.00	1.08E-12	-2.57	1.45E-67	-1734.85
8	TCF12	3.93E-54	-1426.39	2.26E-54	-1432.76	5.21E-24	601.00	1.28E-55	-1458.01	3.30E-62	-1595.38
9	ZNF131	4.28E-50	-1317.67	2.60E-50	-1323.55	9.17E-17	410.69	1.82E-51	-1346.88	1.55E-57	-1473.76
10	ITSN2	3.66E-49	-1292.48	3.16E-26	-7.07	8.04E-44	1121.76	1.66E-50	-1321.13	1.87E-56	-1445.59
11	ENTPD6	4.56E-47	-1236.11	2.94E-47	-1241.64	9.46E-158	4147.98	2.38E-48	-1263.52	4.87E-54	-1382.54
12	DMTN(NM_001323385)	6.96E-44	-1150.49	4.73E-24	-7.42	7.61E-150	3935.90	1.37E-17	-4.44	1.67E-20	-4.84
13	GLYR1(NR_136698)	4.11E-43	-1129.74	2.39E-13	-3.41	3.63E-42	1078.21	2.84E-44	-1154.78	1.85E-14	-3.37
14	MTMR1	5.70E-35	-911.09	4.13E-35	-915.16	2.42E-14	3.09	6.69E-36	-931.29	4.63E-40	-1018.99
15	NEDD1	3.20E-33	-864.20	3.37E-09	-3.02	2.32E-13	6.54	4.22E-34	-883.35	4.81E-38	-966.53
16	CSDE1	1.21E-32	-848.68	8.96E-33	-852.47	1.31E-35	2.29	1.67E-33	-867.48	2.25E-37	-949.18
17	FAM207A	1.12E-28	-742.07	8.79E-29	-745.38	7.66E-07	4.72	1.93E-09	-3.46	1.34E-09	-3.28
18	INO80	1.75E-27	-710.07	1.40E-27	-713.24	2.40E-08	4.76	3.45E-28	-725.80	4.75E-18	-8.43
19	CEP83	4.26E-26	-672.80	3.47E-26	-675.80	1.44E-10	2.57	9.24E-27	-687.70	8.26E-30	-752.44
20	MYBL1	1.70E-24	-629.78	1.84E-12	-6.20	1.97E-13	4.60	3.45E-18	-15.45	9.21E-18	-10.67
21	CASP10	8.86E-24	-610.46	7.52E-24	-613.19	2.20E-31	3.58	4.42E-08	-3.51	4.05E-27	-682.72
22	FTO	1.16E-22	-580.41	1.73E-05	-2.61	2.85E-05	2.07	3.26E-23	-593.27	9.45E-11	-4.77
23	LMBR1	4.08E-21	-538.89	3.59E-21	-541.29	3.37E-13	2.01	1.28E-21	-550.82	4.82E-24	-602.66
24	GART	2.23E-20	-519.10	1.97E-20	-521.42	3.59E-15	368.83	7.33E-21	-530.59	3.44E-23	-580.52
25	MACO1	7.13E-20	-505.55	2.13E-08	-4.55	2.62E-21	530.00	3.88E-05	-2.68	1.30E-22	-565.37
26	FBXO46	1.74E-19	-495.16	2.75E-09	-5.42	1.19E-28	723.03	6.08E-20	-506.12	3.65E-22	-553.75
27	ARHGAP29	2.63E-18	-463.47	2.64E-03	-2.06	1.03E-126	3316.94	1.00E-18	-473.73	8.50E-21	-518.29
28	CLIP2	5.40E-18	-455.09	6.75E-07	-3.95	1.55E-07	3.05	2.23E-08	-5.06	1.37E-04	-2.52
29	ZNF432	4.44E-17	-430.43	4.14E-17	-432.35	8.06E-32	806.25	1.84E-17	-439.96	2.25E-19	-481.34
30	SLC25A45(NM_001352382)	4.09E-16	-404.44	6.63E-07	-4.50	2.96E-02	2.24	8.50E-04	-2.42	3.88E-09	-6.11

31	PCM1	1.72E-15	-387.72	1.64E-15	-389.45	9.41E-25	620.53	7.95E-16	-396.30	1.54E-17	-433.57
32	TTC3	1.82E-15	-387.03	1.74E-15	-388.76	2.13E-29	742.64	8.46E-16	-395.59	2.92E-04	-2.62
33	GTF2I	3.88E-14	-351.29	3.75E-14	-352.86	1.36E-20	9.92	1.97E-14	-359.06	5.60E-16	-392.82
34	ATP5F1A	8.11E-14	-342.68	7.88E-14	-344.21	1.38E-03	2.66	4.19E-14	-350.26	6.83E-05	-3.23
35	MFSD11(NR_148242)	1.43E-13	-336.09	1.39E-13	-337.58	4.19E-03	2.49	4.67E-09	-10.24	2.51E-15	-375.81
36	SEC24B(NM_001300813)	2.27E-13	-330.67	2.22E-13	-332.14	9.98E-15	357.13	1.32E-02	-2.03	4.29E-15	-369.75
37	ZNF266(NM_001370384)	1.76E-12	-306.76	1.74E-12	-308.13	8.08E-07	3.52	9.93E-13	-313.54	4.55E-14	-343.01
38	RIPOR1	3.51E-12	-298.68	3.47E-12	-300.01	2.90E-29	2.59	2.02E-12	-305.28	3.44E-06	-4.94
39	NT5C2	6.70E-12	-291.21	6.59E-12	-292.51	4.65E-86	2236.20	3.88E-12	-297.65	2.11E-13	-325.62
40	MFSD8	1.48E-11	-281.87	1.44E-05	-5.10	1.82E-03	2.49	8.79E-12	-288.10	5.32E-13	-315.16
41	ZFP30	7.75E-11	-262.51	7.70E-11	-263.68	9.62E-14	8.01	4.82E-11	-268.31	2.80E-03	-2.68
42	CTH	1.00E-10	-259.49	9.98E-11	-260.65	4.42E-09	208.71	6.29E-11	-265.23	4.80E-12	-290.14
43	DOCK7	1.11E-10	-258.27	1.11E-10	-259.42	6.37E-04	2.04	7.00E-11	-263.98	1.35E-02	-2.23
44	HERC3	6.56E-10	-237.45	6.60E-10	-238.51	9.01E-11	253.16	4.34E-10	-242.70	1.45E-03	-3.12
45	ZNF875	7.80E-10	-235.40	7.86E-10	-236.45	3.40E-08	4.43	5.20E-10	-240.60	5.21E-11	-263.19
46	TWF1	8.43E-10	-234.47	8.51E-10	-235.51	2.42E-23	583.47	5.64E-10	-239.65	5.71E-11	-262.15
47	POMT1	9.75E-10	-232.76	9.85E-10	-233.80	5.29E-19	469.46	6.55E-10	-237.90	6.76E-11	-260.24
48	PPP1R9A	1.38E-09	-228.72	1.40E-09	-229.73	1.81E-10	245.21	9.34E-10	-233.77	1.00E-10	-255.71
49	PLEKHB2	1.83E-09	-225.36	1.86E-09	-226.36	1.21E-18	460.10	1.25E-09	-230.33	1.40E-10	-251.96
50	NCOA2	3.17E-09	-218.97	3.23E-09	-219.94	4.65E-114	2979.78	2.19E-09	-223.80	2.62E-10	-244.81
51	FZD3	4.32E-09	-215.32	4.43E-09	-216.27	7.60E-31	3.05	3.37E-03	-3.12	9.32E-05	-4.99
52	DACT2(NM_001286350)	5.69E-09	-212.06	5.87E-09	-213.00	2.66E-45	1160.59	4.02E-09	-216.74	5.18E-10	-237.08
53	ZFP37	1.71E-08	-199.15	1.79E-08	-200.04	5.33E-09	206.57	1.24E-08	-203.55	1.84E-09	-222.65
54	CTNND1	1.93E-08	-197.71	2.02E-08	-198.59	2.11E-52	1347.39	1.41E-08	-202.07	2.12E-09	-221.03
55	LOC102724788	2.39E-08	-195.22	1.68E-02	-2.59	7.59E-35	885.79	1.75E-08	-199.53	2.70E-09	-218.25
56	HFE	2.89E-08	-192.99	3.01E-08	-193.84	1.25E-10	249.41	2.13E-08	-197.24	3.38E-09	-215.75
57	NRG1	3.13E-08	-192.03	3.26E-08	-192.88	1.90E-12	12.27	2.31E-08	-196.27	3.71E-09	-214.68
58	DLG1	3.41E-08	-191.02	3.56E-08	-191.87	5.04E-20	496.29	2.52E-08	-195.24	2.76E-05	-7.29
59	ZNF28	3.60E-08	-190.37	3.76E-08	-191.22	1.30E-18	459.27	2.67E-08	-194.57	4.37E-09	-212.82
60	TMEM182	4.53E-08	-187.72	4.71E-08	-188.55	3.36E-02	2.02	3.37E-08	-191.86	9.62E-05	-6.01
61	RCC1	4.73E-08	-187.21	4.92E-08	-188.04	8.01E-13	307.06	3.52E-08	-191.34	5.96E-09	-209.28
62	DOCK1	1.25E-07	-175.80	1.31E-07	-176.58	2.23E-07	3.04	9.58E-08	-179.68	1.83E-08	-196.53
63	C19orf12	3.64E-07	-163.24	3.84E-07	-163.96	4.39E-03	3.24	2.87E-07	-166.84	6.31E-08	-182.47
64	PFKM	9.94E-07	-151.44	1.05E-06	-152.11	2.15E-08	190.66	8.07E-07	-154.78	1.98E-07	-169.27
65	OSGEPL1	2.01E-06	-143.16	2.14E-06	-143.80	2.52E-03	2.06	1.66E-06	-146.31	4.43E-07	-160.01
66	ACBD5	2.34E-06	-141.34	2.50E-06	-141.97	2.76E-29	739.72	1.95E-06	-144.45	5.28E-07	-157.98
67	RASA4	3.52E-06	-136.60	3.75E-06	-137.21	3.00E-13	318.26	2.94E-06	-139.61	8.41E-07	-152.68
68	PRPF4	2.26E-80	-9.24	4.52E-101	-18.46	8.87E-22	2.30	3.51E-139	-3707.39	2.75E-28	-2.47
69	YTHDF2	3.41E-09	-8.73	6.23E-15	-373.80	2.03E-15	6.64	3.51E-09	-8.41	7.21E-17	-416.14
70	ZCCHC7	8.32E-06	-5.65	2.95E-11	-274.91	1.36E-02	2.31	1.22E-05	-5.29	1.18E-12	-306.02
71	SCAF8	2.40E-27	-5.51	3.50E-60	-1589.47	9.65E-40	6.69	1.39E-61	-1617.49	6.76E-69	-1769.89
72	ZBTB8A	1.29E-06	-5.21	1.35E-06	-5.22	1.44E-10	247.77	5.12E-14	-347.93	1.64E-15	-380.64
73	ZDHHC20	4.20E-16	-5.15	2.23E-36	-949.21	8.97E-13	2.53	7.07E-11	-3.23	1.59E-41	-1056.91
74	CEP112	5.76E-04	-4.01	3.03E-04	-4.50	3.05E-11	265.55	1.92E-09	-225.35	3.15E-03	-2.97
75	RPL10	6.30E-08	-3.83	3.51E-05	-2.58	1.62E-02	2.11	6.10E-06	-2.85	6.86E-04	-2.05
76	PHACTR4	1.30E-27	-3.59	7.10E-88	-2336.66	1.55E-12	299.47	5.51E-90	-2377.85	1.02E-100	-2601.94
77	SUMF2	9.48E-08	-3.52	6.04E-09	-4.17	1.17E-04	4.07	1.77E-23	-600.28	4.02E-26	-656.78
78	ATP2B1	2.61E-07	-3.26	1.44E-10	-5.12	2.38E-08	4.16	7.04E-24	-610.98	1.43E-26	-668.49
79	TCF7L2	5.18E-04	-3.00	1.09E-12	-313.52	8.68E-05	2.49	6.23E-13	-319.03	7.47E-05	-3.46
80	RNF24	9.12E-06	-2.96	5.59E-20	-509.24	1.88E-04	2.97	7.32E-07	-3.49	1.13E-22	-566.97
81	WDR31	7.30E-07	-2.81	2.90E-26	-677.93	2.78E-14	13.39	9.46E-19	-13.66	6.70E-30	-754.82
82	SECISBP2	2.14E-16	-2.81	3.55E-69	-1831.76	7.27E-74	1913.77	8.34E-71	-1864.05	3.68E-14	-2.40
83	SIRT5	1.57E-02	-2.55	1.68E-08	-200.76	1.08E-08	198.51	1.17E-08	-204.29	1.72E-09	-223.45
84	EXOC7	1.67E-02	-2.46	7.12E-09	-210.73	2.75E-03	3.06	4.89E-09	-214.43	1.14E-02	-2.55
85	HARS1	1.42E-02	-2.43	5.56E-03	-2.83	8.72E-03	2.15	1.37E-09	-229.31	1.55E-10	-250.83
86	ERCC6L2	1.43E-07	-2.32	4.61E-24	-8.97	1.83E-08	192.52	8.41E-29	-14.08	5.19E-46	-1173.65
87	CCNE1	2.39E-03	-2.31	3.05E-03	-2.28	9.25E-05	2.27	3.16E-09	-8.80	3.72E-10	-9.14
88	C2orf42(NM_001348759)	1.02E-06	-2.26	8.64E-37	-960.26	2.01E-17	428.00	4.34E-10	-3.00	2.10E-14	-3.91
89	BBS9	1.67E-04	-2.25	8.20E-23	-585.34	8.24E-36	911.09	6.71E-05	-2.37	6.30E-26	-651.71
90	SENP5	4.83E-09	-2.22	1.07E-14	-3.11	3.38E-02	2.17	3.71E-15	-3.15	1.49E-14	-2.87
91	RNF38	1.08E-03	-2.22	2.20E-03	-2.11	4.23E-18	15.05	2.71E-05	-2.94	3.94E-06	-3.19
92	ARPC3	8.32E-06	-2.15	1.40E-06	-2.32	4.80E-05	2.14	5.08E-09	-2.89	2.88E-10	-3.03
93	FAM239A	2.74E-03	-2.12	6.95E-12	-12.97	4.77E-05	2.87	6.62E-06	-3.46	5.72E-08	-4.60
94	FKTN	5.60E-04	-2.08	2.58E-04	-2.20	2.34E-04	3.07	1.22E-12	-7.01	5.39E-26	-653.48
95	ALDH3A2	6.55E-03	-2.07	3.28E-03	-2.24	9.63E-14	331.21	2.16E-03	-2.32	1.06E-03	-2.40
96	MAPK9	1.32E-08	-2.06	1.69E-59	-1570.90	6.08E-26	2.52	7.04E-61	-1598.59	4.10E-68	-1749.20

Table S5. Highly confident (significant for all 4 statistical tests) circRNA-miRNA-mRNA triplets.

No.	circRNA ID	circRNA FC	circRNA Clinical association	Common miRNA ID	miRNA FC	miRNA Clinical association	Competing mRNA ID	mRNA FC	mRNA Clinical association	ceRNA (statistics) P-Value (Hypergeometric Test)	Pearson Correlation Coefficient (PC)	P-value (PC)	P-value (PPC)	P-value (CMI)	Sensitivity correlation, S (SPPC)
1	circR404935	-1.99	TG OS	miR-106b-3p	2.12	TG OS	SELP	-2.73	TC	9.41E-06	0.32	1.11E-03	4.57E-13	1.24E-02	0.33
2	circR404935	-1.99	TG OS	miR-877-3p	1.58	TG	ABCG1	-2.09	TG	4.23E-04	0.49	3.05E-07	4.57E-13	2.19E-02	0.34
3	circR404935	-1.99	TG OS	miR-1301-3p	2.42	TG	ABCG1	-2.09	TG	4.23E-04	0.49	3.05E-07	4.57E-13	2.19E-02	0.34
4	circR404935	-1.99	TG OS	miR-18a-3p	1.70	TG	ABCG1	-2.09	TG	4.23E-04	0.49	3.05E-07	4.57E-13	2.19E-02	0.34
5	circR404935	-1.99	TG OS	miR-185-3p	1.51	TG	ABCG1	-2.09	TG	4.23E-04	0.49	3.05E-07	4.57E-13	2.19E-02	0.34
6	circR404935	-1.99	TG OS	miR-877-5p	2.41	TG	RNF125	-3.16	TG	2.43E-02	0.56	2.17E-09	4.57E-13	9.85E-03	0.36
7	circR404935	-1.99	TG OS	miR-7974	1.61	TG	RNF125	-3.16	TG	2.43E-02	0.56	2.17E-09	4.57E-13	9.85E-03	0.36
8	circR404935	-1.99	TG OS	miR-330-5p	1.58	TC	RNF125	-3.16	TG	2.43E-02	0.56	2.17E-09	4.57E-13	9.85E-03	0.36
9	circR404935	-1.99	TG OS	miR-760	1.74	TG	RNF125	-3.16	TG	2.43E-02	0.56	2.17E-09	4.57E-13	9.85E-03	0.36
10	circR404935	-1.99	TG OS	miR-185-3p	1.51	TG	RNF125	-3.16	TG	2.43E-02	0.56	2.17E-09	4.57E-13	9.85E-03	0.36
11	circR404935	-1.99	TG OS	miR-484	1.84	OS	RNF125	-3.16	TG	2.43E-02	0.56	2.17E-09	4.57E-13	9.85E-03	0.36
12	circR404935	-1.99	TG OS	miR-7974	1.61	TG	SELP	-2.73	TC	9.41E-06	0.32	1.11E-03	4.57E-13	1.24E-02	0.33
13	circR404935	-1.99	TG OS	miR-877-3p	1.58	TG	SELP	-2.73	TC	9.41E-06	0.32	1.11E-03	4.57E-13	1.24E-02	0.33
14	circR404935	-1.99	TG OS	miR-185-3p	1.51	TG	SELP	-2.73	TC	9.41E-06	0.32	1.11E-03	4.57E-13	1.24E-02	0.33
15	circR404935	-1.99	TG OS	miR-1301-3p	2.42	TG	SELP	-2.73	TC	9.41E-06	0.32	1.11E-03	4.57E-13	1.24E-02	0.33
16	circR404935	-1.99	TG OS	miR-7974	1.61	TG	ABCG1	-2.09	TG	4.23E-04	0.49	3.05E-07	4.57E-13	2.19E-02	0.34
17	circR404935	-1.99	TG OS	miR-760	1.74	TG	ABCG1	-2.09	TG	4.23E-04	0.49	3.05E-07	4.57E-13	2.19E-02	0.34
18	circR405853	-1.78	TG	miR-93-5p	2.57	TG OS	RNF180	-3.04	TI	1.59E-07	0.39	8.42E-05	3.88E-33	1.69E-02	0.37
19	circR006909	-1.63		miR-425-5p	2.44	TG TI	DMD	-2.04	VI CS	1.21E-05	0.45	2.78E-06	3.23E-42	3.95E-03	0.31
20	circR006909	-1.63		miR-155-5p	2.55	TG TC	DMD	-2.04	VI CS	1.21E-05	0.45	2.78E-06	3.23E-42	3.95E-03	0.31
21	circR404935	-1.99	TG OS	miR-671-5p	1.67		SELP	-2.73	TC	9.41E-06	0.32	1.11E-03	4.57E-13	1.24E-02	0.33
22	circR404935	-1.99	TG OS	miR-1301-3p	2.42	TG	AC006156.1	-2.05		1.28E-03	0.47	1.02E-06	2.24E-09	2.18E-02	0.33
23	circR404935	-1.99	TG OS	miR-3909	1.83	TG	AC006156.1	-2.05		1.28E-03	0.47	1.02E-06	2.24E-09	2.18E-02	0.33
24	circR404935	-1.99	TG OS	miR-7974	1.61	TG	AC006156.1	-2.05		1.28E-03	0.47	1.02E-06	2.24E-09	2.18E-02	0.33
25	circR404935	-1.99	TG OS	miR-877-3p	1.58	TG	AC006156.1	-2.05		1.28E-03	0.47	1.02E-06	2.24E-09	2.18E-02	0.33
26	circR404935	-1.99	TG OS	miR-361-3p	1.53	TG	GRIA3	-5.44		7.81E-03	0.31	1.80E-03	4.57E-13	3.03E-02	0.40
27	circR404935	-1.99	TG OS	miR-760	1.74	TG	GRIA3	-5.44		7.81E-03	0.31	1.80E-03	4.57E-13	3.03E-02	0.40
28	circR404935	-1.99	TG OS	miR-7974	1.61	TG	GRIA3	-5.44		7.81E-03	0.31	1.80E-03	4.57E-13	3.03E-02	0.40
29	circR404935	-1.99	TG OS	miR-877-3p	1.58	TG	GRIA3	-5.44		7.81E-03	0.31	1.80E-03	4.57E-13	3.03E-02	0.40
30	circR404935	-1.99	TG OS	miR-484	1.84	OS	GRIA3	-5.44		7.81E-03	0.31	1.80E-03	4.57E-13	3.03E-02	0.40
31	circR404935	-1.99	TG OS	miR-877-3p	1.58	TG	ECM2	-2.02		4.85E-02	0.38	1.02E-04	1.62E-07	4.90E-03	0.33
32	circR404935	-1.99	TG OS	miR-1301-3p	2.42	TG	ECM2	-2.02		4.85E-02	0.38	1.02E-04	1.62E-07	4.90E-03	0.33
33	circR404935	-1.99	TG OS	miR-7974	1.61	TG	ECM2	-2.02		4.85E-02	0.38	1.02E-04	1.62E-07	4.90E-03	0.33
34	circR404935	-1.99	TG OS	miR-1301-3p	2.42	TG	SPTBN2	-2.02		4.80E-07	0.35	4.46E-04	4.86E-24	3.36E-03	0.33
35	circR404935	-1.99	TG OS	miR-4326	2.35	TG	SPTBN2	-2.02		4.80E-07	0.35	4.46E-04	4.86E-24	3.36E-03	0.33
36	circR404935	-1.99	TG OS	miR-185-3p	1.51	TG	SPTBN2	-2.02		4.80E-07	0.35	4.46E-04	4.86E-24	3.36E-03	0.33
37	circR404935	-1.99	TG OS	miR-877-5p	2.41	TG	SPTBN2	-2.02		4.80E-07	0.35	4.46E-04	4.86E-24	3.36E-03	0.33
38	circR404935	-1.99	TG OS	miR-484	1.84	OS	SPTBN2	-2.02		4.80E-07	0.35	4.46E-04	4.86E-24	3.36E-03	0.33
39	circR404935	-1.99	TG OS	miR-3909	1.83	TG	SPTBN2	-2.02		4.80E-07	0.35	4.46E-04	4.86E-24	3.36E-03	0.33
40	circR404935	-1.99	TG OS	miR-7974	1.61	TG	SPTBN2	-2.02		4.80E-07	0.35	4.46E-04	4.86E-24	3.36E-03	0.33
41	circR404935	-1.99	TG OS	miR-760	1.74	TG	SPTBN2	-2.02		4.80E-07	0.35	4.46E-04	4.86E-24	3.36E-03	0.33
42	circR404935	-1.99	TG OS	miR-877-3p	1.58	TG	SPTBN2	-2.02		4.80E-07	0.35	4.46E-04	4.86E-24	3.36E-03	0.33
43	circR404935	-1.99	TG OS	miR-330-5p	1.58	TC	SPTBN2	-2.02		4.80E-07	0.35	4.46E-04	4.86E-24	3.36E-03	0.33
44	circR404935	-1.99	TG OS	miR-361-3p	1.53	TG	SPTBN2	-2.02		4.80E-07	0.35	4.46E-04	4.86E-24	3.36E-03	0.33
45	circR404935	-1.99	TG OS	miR-877-5p	2.41	TG	MRO	-3.36		3.17E-04	0.38	1.02E-04	9.80E-17	2.29E-02	0.34
46	circR404935	-1.99	TG OS	miR-4326	2.35	TG	MRO	-3.36		3.17E-04	0.38	1.02E-04	9.80E-17	2.29E-02	0.34
47	circR404935	-1.99	TG OS	miR-7974	1.61	TG	MRO	-3.36		3.17E-04	0.38	1.02E-04	9.80E-17	2.29E-02	0.34
48	circR404935	-1.99	TG OS	miR-330-5p	1.58	TC	MRO	-3.36		3.17E-04	0.38	1.02E-04	9.80E-17	2.29E-02	0.34
49	circR404935	-1.99	TG OS	miR-185-3p	1.51	TG	MRO	-3.36		3.17E-04	0.38	1.02E-04	9.80E-17	2.29E-02	0.34
50	circR404935	-1.99	TG OS	miR-1301-3p	2.42	TG	MRO	-3.36		3.17E-04	0.38	1.02E-04	9.80E-17	2.29E-02	0.34
51	circR404935	-1.99	TG OS	miR-760	1.74	TG	MRO	-3.36		3.17E-04	0.38	1.02E-04	9.80E-17	2.29E-02	0.34
52	circR402748	1.62	OS	miR-214-3p	-2.56	TC	PLCB1	3.83	TG	0.00E+00	0.35	3.58E-04	1.23E-05	3.35E-03	0.35
53	circR405853	-1.78	TG	miR-760	1.74	TG	RNF180	-3.04	TI	1.59E-07	0.39	8.42E-05	3.88E-33	1.69E-02	0.37
54	circR405853	-1.78	TG	miR-500a-3p	2.26	TG	RNF180	-3.04	TI	1.59E-07	0.39	8.42E-05	3.88E-33	1.69E-02	0.37
55	circR405853	-1.78	TG	miR-7974	1.61	TG	RNF180	-3.04	TI	1.59E-07	0.39	8.42E-05	3.88E-33	1.69E-02	0.37
56	circR405853	-1.78	TG	miR-222-3p	3.37	TG	RNF180	-3.04	TI	1.59E-07	0.39	8.42E-05	3.88E-33	1.69E-02	0.37
57	circR405853	-1.78	TG	miR-185-5p	1.79	TG	RNF180	-3.04	TI	1.59E-07	0.39	8.42E-05	3.88E-33	1.69E-02	0.37
58	circR405853	-1.78	TG	miR-339-5p	2.08	TG	RNF180	-3.04	TI	1.59E-07	0.39	8.42E-05	3.88E-33	1.69E-02	0.37
59	circR405853	-1.78	TG	miR-1180-3p	3.09	TG	RNF180	-3.04	TI	1.59E-07	0.39	8.42E-05	3.88E-33	1.69E-02	0.37
60	circR405853	-1.78	TG	miR-452-5p	6.44	TS	RNF180	-3.04	TI	1.59E-07	0.39	8.42E-05	3.88E-33	1.69E-02	0.37
61	circR405853	-1.78	TG	miR-574-5p	1.77	TG	RNF180	-3.04	TI	1.59E-07	0.39	8.42E-05	3.88E-33	1.69E-02	0.37
62	circR405853	-1.78	TG	miR-501-5p	2.38	TG	RNF180	-3.04	TI	1.59E-07	0.39	8.42E-05	3.88E-33	1.69E-02	0.37
63	circR405853	-1.78	TG	miR-1301-3p	2.42	TG	RNF180	-3.04	TI	1.59E-07	0.39	8.42E-05	3.88E-33	1.69E-02	0.37
64	circR405853	-1.78	TG	miR-25-3p	2.58	TG	FBLN5	-3.52	TC	3.75E-02	0.29	3.52E-03	2.24E-09	2.70E-03	0.36
65	circR405853	-1.78	TG	miR-942-5p	1.58	TG	FBLN5	-3.52	TC	3.75E-02	0.29	3.52E-03	2.24E-09	2.70E-03	0.36
66	circR405853	-1.78	TG	miR-151a-3p	2.32	TG	FBLN5	-3.52	TC	3.75E-02	0.29	3.52E-03	2.24E-09	2.70E-03	0.36
67	circR405853	-1.78	TG	miR-25-5p	1.86	TC	FBLN5	-3.52	TC	3.75E-02	0.29	3.52E-03	2.24E-09	2.70E-03	0.36
68	circR400620	-2.04	OS	miR-760	1.74	TG	SEPT4	-2.13	TI	9.49E-05	0.29	3.54E-03	1.62E-07	4.31E-02	0.30
69	circR400620	-2.04	OS	miR-501-5p	2.38	TG	SEPT4	-2.13	TI	9.49E-05	0.29	3.54E-03	1.62E-07	4.31E-02	0.30

70	circR005131	1.82			miR-378a-3p	-1.72			ENOX2	2.24	TS	TG	OS	7.28E-08	0.29	3.44E-03	2.24E-09	5.33E-03	0.38
71	circR005131	1.82			miR-378i	-1.61			ENOX2	2.24	TS	TG	OS	7.28E-08	0.29	3.44E-03	2.24E-09	5.33E-03	0.38
72	circR005131	1.82			miR-4686	-8.16			ENOX2	2.24	TS	TG	OS	7.28E-08	0.29	3.44E-03	2.24E-09	5.33E-03	0.38
73	circR005131	1.82			miR-378c	-1.57			ENOX2	2.24	TS	TG	OS	7.28E-08	0.29	3.44E-03	2.24E-09	5.33E-03	0.38
74	circR005131	1.82			miR-378d	-1.64	TI		BCL9	2.14	TI			0.00E+00	0.34	6.81E-04	3.17E-11	4.64E-02	0.32
75	circR006909	-1.63			miR-877-5p	2.41	TG		DMD	-2.04	VI	CS		1.21E-05	0.45	2.78E-06	3.23E-42	3.95E-03	0.31
76	circR006909	-1.63			miR-1303	2.25	TG		DMD	-2.04	VI	CS		1.21E-05	0.45	2.78E-06	3.23E-42	3.95E-03	0.31
77	circR006909	-1.63			miR-98-5p	1.87	TI		DMD	-2.04	VI	CS		1.21E-05	0.45	2.78E-06	3.23E-42	3.95E-03	0.31
78	circR006909	-1.63			miR-942-5p	1.58	TG		DMD	-2.04	VI	CS		1.21E-05	0.45	2.78E-06	3.23E-42	3.95E-03	0.31
79	circR006909	-1.63			miR-3909	1.83	TG		DMD	-2.04	VI	CS		1.21E-05	0.45	2.78E-06	3.23E-42	3.95E-03	0.31
80	circR006909	-1.63			miR-3591-5p	3.26	TG		DMD	-2.04	VI	CS		1.21E-05	0.45	2.78E-06	3.23E-42	3.95E-03	0.31
81	circR006909	-1.63			miR-760	1.74	TG		DMD	-2.04	VI	CS		1.21E-05	0.45	2.78E-06	3.23E-42	3.95E-03	0.31
82	circR006909	-1.63			miR-1301-3p	2.42	TG		DMD	-2.04	VI	CS		1.21E-05	0.45	2.78E-06	3.23E-42	3.95E-03	0.31
83	circR006909	-1.63			miR-185-5p	1.79	TG		DMD	-2.04	VI	CS		1.21E-05	0.45	2.78E-06	3.23E-42	3.95E-03	0.31
84	circR006909	-1.63			miR-24-2-5p	1.58	TC		DMD	-2.04	VI	CS		1.21E-05	0.45	2.78E-06	3.23E-42	3.95E-03	0.31
85	circR006909	-1.63			miR-25-3p	2.58	TG		DMD	-2.04	VI	CS		1.21E-05	0.45	2.78E-06	3.23E-42	3.95E-03	0.31
86	circR006909	-1.63			miR-330-5p	1.58	TC		DMD	-2.04	VI	CS		1.21E-05	0.45	2.78E-06	3.23E-42	3.95E-03	0.31
87	circR006909	-1.63			miR-452-5p	6.44	TS		DMD	-2.04	VI	CS		1.21E-05	0.45	2.78E-06	3.23E-42	3.95E-03	0.31
88	circR006909	-1.63			miR-574-5p	1.77	TG		DMD	-2.04	VI	CS		1.21E-05	0.45	2.78E-06	3.23E-42	3.95E-03	0.31
89	circR006909	-1.63			miR-500a-5p	1.71	TG		DMD	-2.04	VI	CS		1.21E-05	0.45	2.78E-06	3.23E-42	3.95E-03	0.31
90	circR006909	-1.63			miR-7974	1.61	TG		DMD	-2.04	VI	CS		1.21E-05	0.45	2.78E-06	3.23E-42	3.95E-03	0.31
91	circR006909	-1.63			miR-1306-5p	1.54	TG		DMD	-2.04	VI	CS		1.21E-05	0.45	2.78E-06	3.23E-42	3.95E-03	0.31
92	circR004087	-1.91			miR-339-5p	2.08	TG		TDRD6	-3.31	TG	DC		0.00E+00	0.25	1.39E-02	2.24E-09	1.32E-04	0.34
93	circR004087	-1.91			miR-4326	2.35	TG		TDRD6	-3.31	TG	DC		0.00E+00	0.25	1.39E-02	2.24E-09	1.32E-04	0.34
94	circR004087	-1.91			miR-532-3p	2.17	TG		TDRD6	-3.31	TG	DC		0.00E+00	0.25	1.39E-02	2.24E-09	1.32E-04	0.34
95	circR004087	-1.91			miR-1180-3p	3.09	TG		TDRD6	-3.31	TG	DC		0.00E+00	0.25	1.39E-02	2.24E-09	1.32E-04	0.34
96	circR002149	-1.84			miR-93-5p	2.57	TG	OS	ADAMTSL3	-2.92	TG			9.56E-05	0.44	7.24E-06	2.76E-51	3.44E-02	0.35
97	circR002149	-1.84			miR-140-3p	1.77	TG	DC	ADAMTSL3	-2.92	TG			9.56E-05	0.44	7.24E-06	2.76E-51	3.44E-02	0.35
98	circR002149	-1.84			miR-532-5p	3.05	TG	DC	ADAMTSL3	-2.92	TG			9.56E-05	0.44	7.24E-06	2.76E-51	3.44E-02	0.35
99	circR002149	-1.84			miR-362-5p	2.94	TG	DC	ADAMTSL3	-2.92	TG			9.56E-05	0.44	7.24E-06	2.76E-51	3.44E-02	0.35
100	circR404935	-1.99	TG	OS	miR-671-5p	1.67			GRIA3	-5.44				7.81E-03	0.31	1.80E-03	4.57E-13	3.03E-02	0.40
101	circR404935	-1.99	TG	OS	miR-671-5p	1.67			SPTBN2	-2.02				4.80E-07	0.35	4.46E-04	4.86E-24	3.36E-03	0.33
102	circR404935	-1.99	TG	OS	miR-671-5p	1.67			MRO	-3.36				3.17E-04	0.38	1.02E-04	9.80E-17	2.29E-02	0.34
103	circR074712	1.83	DC		miR-378a-3p	-1.72			ACLY	2.35	TG			9.03E-04	0.31	1.63E-03	1.62E-07	9.19E-03	0.36
104	circR074712	1.83	DC		miR-4686	-8.16			ACLY	2.35	TG			9.03E-04	0.31	1.63E-03	1.62E-07	9.19E-03	0.36
105	circR074712	1.83	DC		miR-378a-5p	-2.14			ACLY	2.35	TG			9.03E-04	0.31	1.63E-03	1.62E-07	9.19E-03	0.36
106	circR402748	1.62	OS		miR-4686	-8.16			PLCB1	3.83	TG			0.00E+00	0.35	3.58E-04	1.23E-05	3.35E-03	0.35
107	circR405853	-1.78	TG		miR-671-5p	1.67			RNF180	-3.04	TI			1.59E-07	0.39	8.42E-05	3.88E-33	1.69E-02	0.37
108	circR405853	-1.78	TG		miR-34a-5p	1.97			RNF180	-3.04	TI			1.59E-07	0.39	8.42E-05	3.88E-33	1.69E-02	0.37
109	circR405853	-1.78	TG		miR-106b-5p	2.59			RNF180	-3.04	TI			1.59E-07	0.39	8.42E-05	3.88E-33	1.69E-02	0.37
110	circR405853	-1.78	TG		miR-17-5p	1.95			RNF180	-3.04	TI			1.59E-07	0.39	8.42E-05	3.88E-33	1.69E-02	0.37
111	circR405853	-1.78	TG		miR-93-3p	1.68			RNF180	-3.04	TI			1.59E-07	0.39	8.42E-05	3.88E-33	1.69E-02	0.37
112	circR400620	-2.04	OS		miR-93-3p	1.68			SEPT4	-2.13	TI			9.49E-05	0.29	3.54E-03	1.62E-07	4.31E-02	0.30
113	circR008561	1.60	OS		miR-378d	-1.64	TI		STIL	2.25				3.73E-11	0.21	3.51E-02	1.38E-14	7.13E-03	0.40
114	circR062722	-1.55	OS		miR-760	1.74	TG		MFAP3L	-2.91				5.38E-03	0.23	2.12E-02	2.24E-09	3.00E-02	0.32
115	circR062722	-1.55	OS		miR-877-3p	1.58	TG		MFAP3L	-2.91				5.38E-03	0.23	2.12E-02	2.24E-09	3.00E-02	0.32
116	circR062722	-1.55	OS		miR-421	2.98	TG		MFAP3L	-2.91				5.38E-03	0.23	2.12E-02	2.24E-09	3.00E-02	0.32
117	circR062722	-1.55	OS		miR-1269a	5.48	TC		MFAP3L	-2.91				5.38E-03	0.23	2.12E-02	2.24E-09	3.00E-02	0.32
118	circR405853	-1.78	TG		miR-501-5p	2.38	TG		ORM1	-2.21				0.00E+00	0.36	2.33E-04	7.70E-11	3.21E-02	0.30
119	circR405853	-1.78	TG		miR-3909	1.83	TG		ORM1	-2.21				0.00E+00	0.36	2.33E-04	7.70E-11	3.21E-02	0.30
120	circR405853	-1.78	TG		miR-7974	1.61	TG		ORM1	-2.21				0.00E+00	0.36	2.33E-04	7.70E-11	3.21E-02	0.30
121	circR005131	1.82			miR-4686	-8.16			BCL9	2.14	TI	OS		0.00E+00	0.34	6.81E-04	3.17E-11	4.64E-02	0.32
122	circR005131	1.82			miR-378a-3p	-1.72			BCL9	2.14	TI	OS		0.00E+00	0.34	6.81E-04	3.17E-11	4.64E-02	0.32
123	circR005131	1.82			miR-378i	-1.61			BCL9	2.14	TI	OS		0.00E+00	0.34	6.81E-04	3.17E-11	4.64E-02	0.32
124	circR005131	1.82			miR-378c	-1.57			BCL9	2.14	TI	OS		0.00E+00	0.34	6.81E-04	3.17E-11	4.64E-02	0.32
125	circR006909	-1.63			miR-34a-5p	1.97			DMD	-2.04	VI	CS		1.21E-05	0.45	2.78E-06	3.23E-42	3.95E-03	0.31
126	circR006909	-1.63			miR-10b-3p	2.24			DMD	-2.04	VI	CS		1.21E-05	0.45	2.78E-06	3.23E-42	3.95E-03	0.31
127	circR006909	-1.63			miR-766-3p	1.56			DMD	-2.04	VI	CS		1.21E-05	0.45	2.78E-06	3.23E-42	3.95E-03	0.31
128	circR071935	1.57			miR-1247-5p	-2.06	TC		PLCE1	3.19	TC			4.54E-03	0.32	1.46E-03	1.23E-05	1.56E-02	0.36
129	circR071935	1.57			miR-1247-5p	-2.06	TC		MCM10	3.89	TG			1.02E-03	0.46	2.30E-06	1.23E-05	9.25E-03	0.37
130	circR089972	-1.57			miR-7-5p	2.96	DC		TDRD15	-3.29	DC			0.00E+00	0.34	5.55E-04	3.17E-11	2.37E-02	0.30
131	circR089972	-1.57			miR-760	1.74	TG		TDRD15	-3.29	DC			0.00E+00	0.34	5.55E-04	3.17E-11	2.37E-02	0.30
132	circR089972	-1.57			miR-1269a	5.48	TC		TDRD15	-3.29	DC			0.00E+00	0.34	5.55E-04	3.17E-11	2.37E-02	0.30
133	circR089972	-1.57			miR-1269b	36.43	OS		TDRD15	-3.29	DC			0.00E+00	0.34	5.55E-04	3.17E-11	2.37E-02	0.30
134	circR089972	-1.57			miR-1301-3p	2.42	TG		TDRD15	-3.29	DC			0.00E+00	0.34	5.55E-04	3.17E-11	2.37E-02	0.30
135	circR006909	-1.63			miR-185-3p	1.51	TG		AHSG	-2.70	DC			1.56E-02	0.24	1.68E-02	1.70E-16	3.79E-02	0.31
136	circR006909	-1.63			miR-7-5p	2.96	DC		AHSG	-2.70	DC			1.56E-02	0.24	1.68E-02	1.70E-16	3.79E-02	0.31
137	circR006909	-1.63			miR-339-3p	1.93	TG		AHSG	-2.70	DC			1.56E-02	0.24	1.68E-02	1.70E-16	3.79E-02	0.31
138	circR006909	-1.63			miR-760	1.74	TG		AHSG	-2.70	DC			1.56E-02	0.24	1.68E-02	1.70E-16	3.79E-02	0.31
139	circR006909	-1.63			miR-7974	1.61	TG		AHSG	-2.70	DC			1.56E-02	0.24	1.68E-02	1.70E-16	3.79E-02	0.31
140	circR006909	-1.63			miR-222-3p	3.37	TG		AHSG	-2.70	DC			1.56E-02	0.24	1.68E-02	1.70E-16	3.79E-02	0.31
141	circR006909	-1.63			miR-339-3p	1.93	TG		APCDD1L	-2.18	TG			3.90E-02	0.32	1.30E-03	1.68E-29	1.43E-02	0.40
142	circR006909	-1.63			miR-423-3p	1.53	TG		APCDD1L	-2.18	TG			3.90E-02	0.32	1.30E-03	1.68E-29	1.43E-02	0.40
143	circR006909	-1.63			miR-185-3p	1.51	TG		APCDD1L	-2.18	TG			3.90E-02	0.32	1.30E-03	1.68E-29	1.43E-02	0.40
144	circR006909	-1.63			miR-941	2.03	TG		APCDD1L	-2.18	TG			3.90E-02	0.32	1.30E-03	1.68E-29	1.43E-02	0.40

145	circR006909	-1.63		miR-877-3p	1.58	TG		APCDD1L	-2.18	TG	3.90E-02	0.32	1.30E-03	1.68E-29	1.43E-02	0.40
146	circR006909	-1.63		miR-7974	1.61	TG		APCDD1L	-2.18	TG	3.90E-02	0.32	1.30E-03	1.68E-29	1.43E-02	0.40
147	circR006909	-1.63		miR-760	1.74	TG		APCDD1L	-2.18	TG	3.90E-02	0.32	1.30E-03	1.68E-29	1.43E-02	0.40
148	circR006909	-1.63		miR-1301-3p	2.42	TG		APCDD1L	-2.18	TG	3.90E-02	0.32	1.30E-03	1.68E-29	1.43E-02	0.40
149	circR006909	-1.63		miR-1180-3p	3.09	TG		APCDD1L	-2.18	TG	3.90E-02	0.32	1.30E-03	1.68E-29	1.43E-02	0.40
150	circR006909	-1.63		miR-330-5p	1.58	TC		APCDD1L	-2.18	TG	3.90E-02	0.32	1.30E-03	1.68E-29	1.43E-02	0.40
151	circR006909	-1.63		miR-25-5p	1.86	TC		APCDD1L	-2.18	TG	3.90E-02	0.32	1.30E-03	1.68E-29	1.43E-02	0.40
152	circR006909	-1.63		miR-877-5p	2.41	TG		APCDD1L	-2.18	TG	3.90E-02	0.32	1.30E-03	1.68E-29	1.43E-02	0.40
153	circR002149	-1.84		miR-361-5p	1.60	TG		ADAMTSL3	-2.92	TG	9.56E-05	0.44	7.24E-06	2.76E-51	3.44E-02	0.35
154	circR002149	-1.84		miR-18a-3p	1.70	TG		ADAMTSL3	-2.92	TG	9.56E-05	0.44	7.24E-06	2.76E-51	3.44E-02	0.35
155	circR002149	-1.84		miR-877-3p	1.58	TG		ADAMTSL3	-2.92	TG	9.56E-05	0.44	7.24E-06	2.76E-51	3.44E-02	0.35
156	circR002149	-1.84		miR-454-3p	2.34	TG		ADAMTSL3	-2.92	TG	9.56E-05	0.44	7.24E-06	2.76E-51	3.44E-02	0.35
157	circR002149	-1.84		miR-1303	2.25	TG		ADAMTSL3	-2.92	TG	9.56E-05	0.44	7.24E-06	2.76E-51	3.44E-02	0.35
158	circR002149	-1.84		miR-185-5p	1.79	TG		ADAMTSL3	-2.92	TG	9.56E-05	0.44	7.24E-06	2.76E-51	3.44E-02	0.35
159	circR002149	-1.84		miR-7974	1.61	TG		ADAMTSL3	-2.92	TG	9.56E-05	0.44	7.24E-06	2.76E-51	3.44E-02	0.35
160	circR002149	-1.84		miR-501-5p	2.38	TG		ADAMTSL3	-2.92	TG	9.56E-05	0.44	7.24E-06	2.76E-51	3.44E-02	0.35
161	circR002149	-1.84		miR-532-3p	2.17	TG		ADAMTSL3	-2.92	TG	9.56E-05	0.44	7.24E-06	2.76E-51	3.44E-02	0.35
162	circR002149	-1.84		miR-942-5p	1.58	TG		ADAMTSL3	-2.92	TG	9.56E-05	0.44	7.24E-06	2.76E-51	3.44E-02	0.35
163	circR002149	-1.84		miR-6500-3p	1.57	TS		ADAMTSL3	-2.92	TG	9.56E-05	0.44	7.24E-06	2.76E-51	3.44E-02	0.35
164	circR002149	-1.84		miR-15b-5p	1.84	TG		ADAMTSL3	-2.92	TG	9.56E-05	0.44	7.24E-06	2.76E-51	3.44E-02	0.35
165	circR002149	-1.84		miR-330-5p	1.58	TC		ADAMTSL3	-2.92	TG	9.56E-05	0.44	7.24E-06	2.76E-51	3.44E-02	0.35
166	circR002149	-1.84		miR-500a-3p	2.26	TG		ADAMTSL3	-2.92	TG	9.56E-05	0.44	7.24E-06	2.76E-51	3.44E-02	0.35
167	circR002149	-1.84		miR-3591-5p	3.26	TG		ADAMTSL3	-2.92	TG	9.56E-05	0.44	7.24E-06	2.76E-51	3.44E-02	0.35
168	circR002149	-1.84		miR-941	2.03	TG		ADAMTSL3	-2.92	TG	9.56E-05	0.44	7.24E-06	2.76E-51	3.44E-02	0.35
169	circR002149	-1.84		miR-1269b	36.43	OS		ADAMTSL3	-2.92	TG	9.56E-05	0.44	7.24E-06	2.76E-51	3.44E-02	0.35
170	circR002149	-1.84		miR-148b-5p	1.61	TG		ADAMTSL3	-2.92	TG	9.56E-05	0.44	7.24E-06	2.76E-51	3.44E-02	0.35
171	circR002149	-1.84		miR-130b-3p	2.51	TG		ADAMTSL3	-2.92	TG	9.56E-05	0.44	7.24E-06	2.76E-51	3.44E-02	0.35
172	circR006909	-1.63		miR-1226-3p	2.05	TG	OS	MROH2A	-3.48		2.84E-04	0.37	1.53E-04	7.61E-46	2.69E-02	0.42
173	circR006909	-1.63		miR-106b-3p	2.12	TG	OS	MROH2A	-3.48		2.84E-04	0.37	1.53E-04	7.61E-46	2.69E-02	0.42
174	circR006909	-1.63		miR-4664-3p	1.75	TG	DC	MROH2A	-3.48		2.84E-04	0.37	1.53E-04	7.61E-46	2.69E-02	0.42
175	circR006909	-1.63		miR-362-5p	2.94	TG	DC	CHRD	-2.22		1.77E-04	0.25	1.13E-02	2.02E-47	1.68E-03	0.34
176	circR006909	-1.63		miR-1226-3p	2.05	TG	OS	CHRD	-2.22		1.77E-04	0.25	1.13E-02	2.02E-47	1.68E-03	0.34
177	circR006909	-1.63		miR-106b-3p	2.12	TG	OS	FNDC4	-2.42		5.65E-03	0.42	2.12E-05	1.11E-27	3.20E-04	0.31
178	circR006909	-1.63		miR-629-5p	1.99	TG	TC	FNDC4	-2.42		5.65E-03	0.42	2.12E-05	1.11E-27	3.20E-04	0.31
179	circR006909	-1.63		miR-532-5p	3.05	TG	DC	ANKRD24	-2.05		3.49E-07	0.28	6.05E-03	3.71E-62	2.29E-02	0.49
180	circR006909	-1.63		miR-4664-3p	1.75	TG	DC	ANKRD24	-2.05		3.49E-07	0.28	6.05E-03	3.71E-62	2.29E-02	0.49
181	circR006909	-1.63		miR-106b-3p	2.12	TG	OS	ANKRD24	-2.05		3.49E-07	0.28	6.05E-03	3.71E-62	2.29E-02	0.49
182	circR006909	-1.63		miR-1226-3p	2.05	TG	OS	ANKRD24	-2.05		3.49E-07	0.28	6.05E-03	3.71E-62	2.29E-02	0.49
183	circR006909	-1.63		miR-140-3p	1.77	TG	DC	AK126380	-3.02		3.31E-06	0.31	1.73E-03	6.55E-55	5.14E-12	0.32
184	circR006909	-1.63		miR-362-5p	2.94	TG	DC	AK126380	-3.02		3.31E-06	0.31	1.73E-03	6.55E-55	5.14E-12	0.32
185	circR006909	-1.63		miR-1226-3p	2.05	TG	OS	AK126380	-3.02		3.31E-06	0.31	1.73E-03	6.55E-55	5.14E-12	0.32
186	circR002149	-1.84		miR-1226-3p	2.05	TG	OS	ANKRD24	-2.05		9.08E-03	0.25	1.28E-02	1.38E-38	2.04E-02	0.43
187	circR002149	-1.84		miR-532-5p	3.05	TG	DC	ANKRD24	-2.05		9.08E-03	0.25	1.28E-02	1.38E-38	2.04E-02	0.43
188	circR002149	-1.84		miR-532-5p	3.05	TG	DC	IYD	-2.47		3.21E-06	0.21	3.50E-02	7.36E-49	1.65E-03	0.35
189	circR002149	-1.84		miR-1226-3p	2.05	TG	OS	IYD	-2.47		3.21E-06	0.21	3.50E-02	7.36E-49	1.65E-03	0.35
190	circR002149	-1.84		miR-93-5p	2.57	TG	OS	IYD	-2.47		3.21E-06	0.21	3.50E-02	7.36E-49	1.65E-03	0.35
191	circR000943	-2.32		miR-532-5p	3.05	TG	DC	IYD	-2.47		4.56E-04	0.45	3.41E-06	1.38E-38	1.80E-02	0.36
192	circR000943	-2.32		miR-1226-3p	2.05	TG	OS	IYD	-2.47		4.56E-04	0.45	3.41E-06	1.38E-38	1.80E-02	0.36
193	circR000943	-2.32		miR-93-5p	2.57	TG	OS	IYD	-2.47		4.56E-04	0.45	3.41E-06	1.38E-38	1.80E-02	0.36
194	circR008561	1.60	OS	miR-490-3p	-1.68			STIL	2.25		3.73E-11	0.21	3.51E-02	1.38E-14	7.13E-03	0.40
195	circR008561	1.60	OS	miR-378i	-1.61			STIL	2.25		3.73E-11	0.21	3.51E-02	1.38E-14	7.13E-03	0.40
196	circR008561	1.60	OS	miR-30c-2-3p	-1.73			STIL	2.25		3.73E-11	0.21	3.51E-02	1.38E-14	7.13E-03	0.40
197	circR008561	1.60	OS	let-7c-5p	-1.79			STIL	2.25		3.73E-11	0.21	3.51E-02	1.38E-14	7.13E-03	0.40
198	circR008561	1.60	OS	miR-378a-3p	-1.72			STIL	2.25		3.73E-11	0.21	3.51E-02	1.38E-14	7.13E-03	0.40
199	circR008561	1.60	OS	miR-378c	-1.57			STIL	2.25		3.73E-11	0.21	3.51E-02	1.38E-14	7.13E-03	0.40
200	circR008561	1.60	OS	miR-4686	-8.16			STIL	2.25		3.73E-11	0.21	3.51E-02	1.38E-14	7.13E-03	0.40
201	circR008561	1.60	OS	miR-4686	-8.16			NCAPD2	2.03		9.79E-09	0.27	6.78E-03	7.97E-13	2.12E-02	0.35
202	circR008561	1.60	OS	miR-378a-3p	-1.72			NCAPD2	2.03		9.79E-09	0.27	6.78E-03	7.97E-13	2.12E-02	0.35
203	circR008561	1.60	OS	miR-378c	-1.57			NCAPD2	2.03		9.79E-09	0.27	6.78E-03	7.97E-13	2.12E-02	0.35
204	circR008561	1.60	OS	let-7c-5p	-1.79			NCAPD2	2.03		9.79E-09	0.27	6.78E-03	7.97E-13	2.12E-02	0.35
205	circR008561	1.60	OS	miR-30c-2-3p	-1.73			NCAPD2	2.03		9.79E-09	0.27	6.78E-03	7.97E-13	2.12E-02	0.35
206	circR008561	1.60	OS	miR-378a-5p	-2.14			NCAPD2	2.03		9.79E-09	0.27	6.78E-03	7.97E-13	2.12E-02	0.35
207	circR405853	-1.78	TG	miR-130b-5p	2.73			ORM1	-2.21		0.00E+00	0.36	2.33E-04	7.70E-11	3.21E-02	0.30
208	circR405853	-1.78	TG	miR-34a-5p	1.97			ORM1	-2.21		0.00E+00	0.36	2.33E-04	7.70E-11	3.21E-02	0.30
209	circR005131	1.82		miR-378c	-1.57			PLCB1	3.83	TG	4.02E-03	0.44	5.54E-06	1.23E-05	2.79E-03	0.47
210	circR005131	1.82		miR-4686	-8.16			PLCB1	3.83	TG	4.02E-03	0.44	5.54E-06	1.23E-05	2.79E-03	0.47
211	circR092505	1.67		miR-4686	-8.16			ERCC6L	14.52	TG	1.30E-04	0.35	4.34E-04	1.23E-05	2.49E-02	0.31
212	circR092505	1.67		miR-378a-3p	-1.72			ERCC6L	14.52	TG	1.30E-04	0.35	4.34E-04	1.23E-05	2.49E-02	0.31
213	circR071935	1.57		miR-4686	-8.16			PLCE1	3.19	TC	4.54E-03	0.32	1.46E-03	1.23E-05	1.56E-02	0.36
214	circR071935	1.57		miR-4686	-8.16			MCM10	3.89	TG	1.02E-03	0.46	2.30E-06	1.23E-05	9.25E-03	0.37
215	circR006909	-1.63		miR-224-5p	6.89			AHSG	-2.70	DC	1.56E-02	0.24	1.68E-02	1.70E-16	3.79E-02	0.31
216	circR006909	-1.63		miR-96-5p	4.60			AHSG	-2.70	DC	1.56E-02	0.24	1.68E-02	1.70E-16	3.79E-02	0.31
217	circR006909	-1.63		miR-3615	1.70			APCDD1L	-2.18	TG	3.90E-02	0.32	1.30E-03	1.68E-29	1.43E-02	0.40
218	circR006909	-1.63		miR-1295a	2.26			APCDD1L	-2.18	TG	3.90E-02	0.32	1.30E-03	1.68E-29	1.43E-02	0.40
219	circR006909	-1.63		miR-652-3p	1.90			APCDD1L	-2.18	TG	3.90E-02	0.32	1.30E-03	1.68E-29	1.43E-02	0.40

220	circR002149	-1.84	miR-224-5p	6.89		ADAMTSL3	-2.92	TG	9.56E-05	0.44	7.24E-06	2.76E-51	3.44E-02	0.35
221	circR002149	-1.84	miR-34a-5p	1.97		ADAMTSL3	-2.92	TG	9.56E-05	0.44	7.24E-06	2.76E-51	3.44E-02	0.35
222	circR002149	-1.84	miR-516b-5p	9.60		ADAMTSL3	-2.92	TG	9.56E-05	0.44	7.24E-06	2.76E-51	3.44E-02	0.35
223	circR002149	-1.84	miR-671-5p	1.67		ADAMTSL3	-2.92	TG	9.56E-05	0.44	7.24E-06	2.76E-51	3.44E-02	0.35
224	circR004796	1.68	miR-214-3p	-2.56	TC	PLOD3	2.07		0.00E+00	0.37	1.93E-04	1.23E-05	4.50E-02	0.35
225	circR006909	-1.63	miR-1180-3p	3.09	TG	MROH2A	-3.48		2.84E-04	0.37	1.53E-04	7.61E-46	2.69E-02	0.42
226	circR006909	-1.63	miR-361-3p	1.53	TG	MROH2A	-3.48		2.84E-04	0.37	1.53E-04	7.61E-46	2.69E-02	0.42
227	circR006909	-1.63	miR-1306-5p	1.54	TG	MROH2A	-3.48		2.84E-04	0.37	1.53E-04	7.61E-46	2.69E-02	0.42
228	circR006909	-1.63	miR-484	1.84	OS	MROH2A	-3.48		2.84E-04	0.37	1.53E-04	7.61E-46	2.69E-02	0.42
229	circR006909	-1.63	miR-1301-3p	2.42	TG	MROH2A	-3.48		2.84E-04	0.37	1.53E-04	7.61E-46	2.69E-02	0.42
230	circR006909	-1.63	miR-532-3p	2.17	TG	MROH2A	-3.48		2.84E-04	0.37	1.53E-04	7.61E-46	2.69E-02	0.42
231	circR006909	-1.63	miR-877-3p	1.58	TG	MROH2A	-3.48		2.84E-04	0.37	1.53E-04	7.61E-46	2.69E-02	0.42
232	circR006909	-1.63	miR-4326	2.35	DC	MROH2A	-3.48		2.84E-04	0.37	1.53E-04	7.61E-46	2.69E-02	0.42
233	circR006909	-1.63	miR-7-5p	2.96	TG	MROH2A	-3.48		2.84E-04	0.37	1.53E-04	7.61E-46	2.69E-02	0.42
234	circR006909	-1.63	miR-7974	1.61	TG	MROH2A	-3.48		2.84E-04	0.37	1.53E-04	7.61E-46	2.69E-02	0.42
235	circR006909	-1.63	miR-423-3p	1.53	TG	MROH2A	-3.48		2.84E-04	0.37	1.53E-04	7.61E-46	2.69E-02	0.42
236	circR006909	-1.63	miR-25-3p	2.58	TG	MROH2A	-3.48		2.84E-04	0.37	1.53E-04	7.61E-46	2.69E-02	0.42
237	circR006909	-1.63	miR-18a-3p	1.70	TG	MROH2A	-3.48		2.84E-04	0.37	1.53E-04	7.61E-46	2.69E-02	0.42
238	circR006909	-1.63	miR-3200-3p	2.01	TG	MROH2A	-3.48		2.84E-04	0.37	1.53E-04	7.61E-46	2.69E-02	0.42
239	circR006909	-1.63	miR-500a-3p	2.26	TG	MROH2A	-3.48		2.84E-04	0.37	1.53E-04	7.61E-46	2.69E-02	0.42
240	circR006909	-1.63	miR-330-5p	1.58	TC	MROH2A	-3.48		2.84E-04	0.37	1.53E-04	7.61E-46	2.69E-02	0.42
241	circR006909	-1.63	miR-182-5p	9.05	OS	MROH2A	-3.48		2.84E-04	0.37	1.53E-04	7.61E-46	2.69E-02	0.42
242	circR006909	-1.63	miR-3909	1.83	TG	CHRD	-2.22		1.77E-04	0.25	1.13E-02	2.02E-47	1.68E-03	0.34
243	circR006909	-1.63	miR-423-3p	1.53	TG	CHRD	-2.22		1.77E-04	0.25	1.13E-02	2.02E-47	1.68E-03	0.34
244	circR006909	-1.63	miR-216a-3p	5.20	TI	CHRD	-2.22		1.77E-04	0.25	1.13E-02	2.02E-47	1.68E-03	0.34
245	circR006909	-1.63	miR-339-3p	1.93	TG	CHRD	-2.22		1.77E-04	0.25	1.13E-02	2.02E-47	1.68E-03	0.34
246	circR006909	-1.63	miR-18a-3p	1.70	TG	CHRD	-2.22		1.77E-04	0.25	1.13E-02	2.02E-47	1.68E-03	0.34
247	circR006909	-1.63	miR-15b-5p	1.84	TG	CHRD	-2.22		1.77E-04	0.25	1.13E-02	2.02E-47	1.68E-03	0.34
248	circR006909	-1.63	miR-877-5p	2.41	TG	CHRD	-2.22		1.77E-04	0.25	1.13E-02	2.02E-47	1.68E-03	0.34
249	circR006909	-1.63	miR-339-5p	2.08	TG	CHRD	-2.22		1.77E-04	0.25	1.13E-02	2.02E-47	1.68E-03	0.34
250	circR006909	-1.63	miR-185-3p	1.51	TG	CHRD	-2.22		1.77E-04	0.25	1.13E-02	2.02E-47	1.68E-03	0.34
251	circR006909	-1.63	miR-1180-3p	3.09	TG	CHRD	-2.22		1.77E-04	0.25	1.13E-02	2.02E-47	1.68E-03	0.34
252	circR006909	-1.63	miR-501-5p	2.38	TG	CHRD	-2.22		1.77E-04	0.25	1.13E-02	2.02E-47	1.68E-03	0.34
253	circR006909	-1.63	miR-1269b	36.43	OS	CHRD	-2.22		1.77E-04	0.25	1.13E-02	2.02E-47	1.68E-03	0.34
254	circR006909	-1.63	miR-760	1.74	TG	CHRD	-2.22		1.77E-04	0.25	1.13E-02	2.02E-47	1.68E-03	0.34
255	circR006909	-1.63	miR-1301-3p	2.42	TG	CHRD	-2.22		1.77E-04	0.25	1.13E-02	2.02E-47	1.68E-03	0.34
256	circR006909	-1.63	miR-330-5p	1.58	TC	CHRD	-2.22		1.77E-04	0.25	1.13E-02	2.02E-47	1.68E-03	0.34
257	circR006909	-1.63	miR-7974	1.61	TG	CHRD	-2.22		1.77E-04	0.25	1.13E-02	2.02E-47	1.68E-03	0.34
258	circR006909	-1.63	miR-484	1.84	OS	CHRD	-2.22		1.77E-04	0.25	1.13E-02	2.02E-47	1.68E-03	0.34
259	circR006909	-1.63	miR-25-5p	1.86	TC	CHRD	-2.22		1.77E-04	0.25	1.13E-02	2.02E-47	1.68E-03	0.34
260	circR006909	-1.63	miR-1269b	36.43	OS	HPD	-5.86		1.56E-02	0.41	3.02E-05	9.80E-17	1.57E-02	0.32
261	circR006909	-1.63	miR-7974	1.61	TG	HPD	-5.86		1.56E-02	0.41	3.02E-05	9.80E-17	1.57E-02	0.32
262	circR006909	-1.63	miR-25-5p	1.86	TC	HPD	-5.86		1.56E-02	0.41	3.02E-05	9.80E-17	1.57E-02	0.32
263	circR006909	-1.63	miR-942-5p	1.58	TG	HPD	-5.86		1.56E-02	0.41	3.02E-05	9.80E-17	1.57E-02	0.32
264	circR006909	-1.63	miR-185-3p	1.51	TG	HPD	-5.86		1.56E-02	0.41	3.02E-05	9.80E-17	1.57E-02	0.32
265	circR006909	-1.63	miR-1269a	5.48	TC	HPD	-5.86		1.56E-02	0.41	3.02E-05	9.80E-17	1.57E-02	0.32
266	circR006909	-1.63	miR-454-3p	2.34	TG	FNDC4	-2.42		5.65E-03	0.42	2.12E-05	1.11E-27	3.20E-04	0.31
267	circR006909	-1.63	miR-1180-3p	3.09	TG	FNDC4	-2.42		5.65E-03	0.42	2.12E-05	1.11E-27	3.20E-04	0.31
268	circR006909	-1.63	miR-216a-3p	5.20	TI	FNDC4	-2.42		5.65E-03	0.42	2.12E-05	1.11E-27	3.20E-04	0.31
269	circR006909	-1.63	miR-423-3p	1.53	TG	FNDC4	-2.42		5.65E-03	0.42	2.12E-05	1.11E-27	3.20E-04	0.31
270	circR006909	-1.63	miR-331-5p	1.62	TG	FNDC4	-2.42		5.65E-03	0.42	2.12E-05	1.11E-27	3.20E-04	0.31
271	circR006909	-1.63	miR-339-5p	2.08	TG	FNDC4	-2.42		5.65E-03	0.42	2.12E-05	1.11E-27	3.20E-04	0.31
272	circR006909	-1.63	miR-185-3p	1.51	TG	FNDC4	-2.42		5.65E-03	0.42	2.12E-05	1.11E-27	3.20E-04	0.31
273	circR006909	-1.63	miR-760	1.74	TG	FNDC4	-2.42		5.65E-03	0.42	2.12E-05	1.11E-27	3.20E-04	0.31
274	circR006909	-1.63	miR-7974	1.61	TG	FNDC4	-2.42		5.65E-03	0.42	2.12E-05	1.11E-27	3.20E-04	0.31
275	circR006909	-1.63	miR-330-5p	1.58	TC	FNDC4	-2.42		5.65E-03	0.42	2.12E-05	1.11E-27	3.20E-04	0.31
276	circR006909	-1.63	miR-501-3p	2.49	TG	FNDC4	-2.42		5.65E-03	0.42	2.12E-05	1.11E-27	3.20E-04	0.31
277	circR006909	-1.63	miR-330-5p	1.58	TC	ANKRD24	-2.05		3.49E-07	0.28	6.05E-03	3.71E-62	2.29E-02	0.49
278	circR006909	-1.63	miR-185-5p	1.79	TG	ANKRD24	-2.05		3.49E-07	0.28	6.05E-03	3.71E-62	2.29E-02	0.49
279	circR006909	-1.63	miR-484	1.84	OS	ANKRD24	-2.05		3.49E-07	0.28	6.05E-03	3.71E-62	2.29E-02	0.49
280	circR006909	-1.63	miR-1301-3p	2.42	TG	ANKRD24	-2.05		3.49E-07	0.28	6.05E-03	3.71E-62	2.29E-02	0.49
281	circR006909	-1.63	miR-3909	1.83	TG	ANKRD24	-2.05		3.49E-07	0.28	6.05E-03	3.71E-62	2.29E-02	0.49
282	circR006909	-1.63	miR-4326	2.35	TG	ANKRD24	-2.05		3.49E-07	0.28	6.05E-03	3.71E-62	2.29E-02	0.49
283	circR006909	-1.63	miR-18a-3p	1.70	TG	ANKRD24	-2.05		3.49E-07	0.28	6.05E-03	3.71E-62	2.29E-02	0.49
284	circR006909	-1.63	miR-185-3p	1.51	TG	ANKRD24	-2.05		3.49E-07	0.28	6.05E-03	3.71E-62	2.29E-02	0.49
285	circR006909	-1.63	miR-942-5p	1.58	TG	ANKRD24	-2.05		3.49E-07	0.28	6.05E-03	3.71E-62	2.29E-02	0.49
286	circR006909	-1.63	miR-339-3p	1.93	TG	ANKRD24	-2.05		3.49E-07	0.28	6.05E-03	3.71E-62	2.29E-02	0.49
287	circR006909	-1.63	miR-1180-3p	3.09	TG	ANKRD24	-2.05		3.49E-07	0.28	6.05E-03	3.71E-62	2.29E-02	0.49
288	circR006909	-1.63	miR-877-3p	1.58	TG	ANKRD24	-2.05		3.49E-07	0.28	6.05E-03	3.71E-62	2.29E-02	0.49
289	circR006909	-1.63	miR-7974	1.61	TG	ANKRD24	-2.05		3.49E-07	0.28	6.05E-03	3.71E-62	2.29E-02	0.49
290	circR006909	-1.63	miR-760	1.74	TG	ANKRD24	-2.05		3.49E-07	0.28	6.05E-03	3.71E-62	2.29E-02	0.49
291	circR006909	-1.63	miR-7-5p	2.96	DC	ANKRD24	-2.05		3.49E-07	0.28	6.05E-03	3.71E-62	2.29E-02	0.49
292	circR006909	-1.63	miR-500a-3p	2.26	TG	ANKRD24	-2.05		3.49E-07	0.28	6.05E-03	3.71E-62	2.29E-02	0.49
293	circR006909	-1.63	miR-423-3p	1.53	TG	ANKRD24	-2.05		3.49E-07	0.28	6.05E-03	3.71E-62	2.29E-02	0.49
294	circR006909	-1.63	miR-574-5p	1.77	TG	ANKRD24	-2.05		3.49E-07	0.28	6.05E-03	3.71E-62	2.29E-02	0.49

295	circR006909	-1.63	miR-25-5p	1.86	TC	ANKRD24	-2.05	3.49E-07	0.28	6.05E-03	3.71E-62	2.29E-02	0.49
296	circR006909	-1.63	miR-1306-5p	1.54	TG	ANKRD24	-2.05	3.49E-07	0.28	6.05E-03	3.71E-62	2.29E-02	0.49
297	circR006909	-1.63	miR-501-5p	2.38	TG	ANKRD24	-2.05	3.49E-07	0.28	6.05E-03	3.71E-62	2.29E-02	0.49
298	circR006909	-1.63	miR-532-3p	2.17	TG	ANKRD24	-2.05	3.49E-07	0.28	6.05E-03	3.71E-62	2.29E-02	0.49
299	circR006909	-1.63	miR-877-3p	1.58	TG	AK126380	-3.02	3.31E-06	0.31	1.73E-03	6.55E-55	5.14E-12	0.32
300	circR006909	-1.63	miR-185-5p	1.79	TG	AK126380	-3.02	3.31E-06	0.31	1.73E-03	6.55E-55	5.14E-12	0.32
301	circR006909	-1.63	miR-1180-3p	3.09	TG	AK126380	-3.02	3.31E-06	0.31	1.73E-03	6.55E-55	5.14E-12	0.32
302	circR006909	-1.63	miR-760	1.74	TG	AK126380	-3.02	3.31E-06	0.31	1.73E-03	6.55E-55	5.14E-12	0.32
303	circR006909	-1.63	miR-942-5p	1.58	TG	AK126380	-3.02	3.31E-06	0.31	1.73E-03	6.55E-55	5.14E-12	0.32
304	circR006909	-1.63	miR-185-3p	1.51	TG	AK126380	-3.02	3.31E-06	0.31	1.73E-03	6.55E-55	5.14E-12	0.32
305	circR006909	-1.63	miR-484	1.84	OS	AK126380	-3.02	3.31E-06	0.31	1.73E-03	6.55E-55	5.14E-12	0.32
306	circR006909	-1.63	miR-4326	2.35	TG	AK126380	-3.02	3.31E-06	0.31	1.73E-03	6.55E-55	5.14E-12	0.32
307	circR006909	-1.63	miR-421	2.98	TG	AK126380	-3.02	3.31E-06	0.31	1.73E-03	6.55E-55	5.14E-12	0.32
308	circR006909	-1.63	miR-7974	1.61	TG	AK126380	-3.02	3.31E-06	0.31	1.73E-03	6.55E-55	5.14E-12	0.32
309	circR006909	-1.63	miR-339-5p	2.08	TG	AK126380	-3.02	3.31E-06	0.31	1.73E-03	6.55E-55	5.14E-12	0.32
310	circR006909	-1.63	miR-18a-3p	1.70	TG	AK126380	-3.02	3.31E-06	0.31	1.73E-03	6.55E-55	5.14E-12	0.32
311	circR006909	-1.63	miR-500a-3p	2.26	TG	AK126380	-3.02	3.31E-06	0.31	1.73E-03	6.55E-55	5.14E-12	0.32
312	circR006909	-1.63	miR-330-5p	1.58	TC	AK126380	-3.02	3.31E-06	0.31	1.73E-03	6.55E-55	5.14E-12	0.32
313	circR006909	-1.63	miR-3909	1.83	TG	AK126380	-3.02	3.31E-06	0.31	1.73E-03	6.55E-55	5.14E-12	0.32
314	circR006909	-1.63	miR-501-5p	2.38	TG	AK126380	-3.02	3.31E-06	0.31	1.73E-03	6.55E-55	5.14E-12	0.32
315	circR006909	-1.63	miR-423-3p	1.53	TG	AK126380	-3.02	3.31E-06	0.31	1.73E-03	6.55E-55	5.14E-12	0.32
316	circR006909	-1.63	miR-877-5p	2.41	TG	AK126380	-3.02	3.31E-06	0.31	1.73E-03	6.55E-55	5.14E-12	0.32
317	circR006909	-1.63	miR-532-3p	2.17	TG	AK126380	-3.02	3.31E-06	0.31	1.73E-03	6.55E-55	5.14E-12	0.32
318	circR002149	-1.84	miR-501-5p	2.38	TG	ANKRD24	-2.05	9.08E-03	0.25	1.28E-02	1.38E-38	2.04E-02	0.43
319	circR002149	-1.84	miR-4326	2.35	TG	ANKRD24	-2.05	9.08E-03	0.25	1.28E-02	1.38E-38	2.04E-02	0.43
320	circR002149	-1.84	miR-3909	1.83	TG	ANKRD24	-2.05	9.08E-03	0.25	1.28E-02	1.38E-38	2.04E-02	0.43
321	circR002149	-1.84	miR-25-5p	1.86	TC	ANKRD24	-2.05	9.08E-03	0.25	1.28E-02	1.38E-38	2.04E-02	0.43
322	circR002149	-1.84	miR-330-5p	1.58	TC	ANKRD24	-2.05	9.08E-03	0.25	1.28E-02	1.38E-38	2.04E-02	0.43
323	circR002149	-1.84	miR-500a-3p	2.26	TG	ANKRD24	-2.05	9.08E-03	0.25	1.28E-02	1.38E-38	2.04E-02	0.43
324	circR002149	-1.84	miR-877-3p	1.58	TG	ANKRD24	-2.05	9.08E-03	0.25	1.28E-02	1.38E-38	2.04E-02	0.43
325	circR002149	-1.84	miR-942-5p	1.58	TG	ANKRD24	-2.05	9.08E-03	0.25	1.28E-02	1.38E-38	2.04E-02	0.43
326	circR002149	-1.84	miR-532-3p	2.17	TG	ANKRD24	-2.05	9.08E-03	0.25	1.28E-02	1.38E-38	2.04E-02	0.43
327	circR002149	-1.84	miR-7-5p	2.96	DC	ANKRD24	-2.05	9.08E-03	0.25	1.28E-02	1.38E-38	2.04E-02	0.43
328	circR002149	-1.84	miR-574-5p	1.77	TG	ANKRD24	-2.05	9.08E-03	0.25	1.28E-02	1.38E-38	2.04E-02	0.43
329	circR002149	-1.84	miR-185-5p	1.79	TG	ANKRD24	-2.05	9.08E-03	0.25	1.28E-02	1.38E-38	2.04E-02	0.43
330	circR002149	-1.84	miR-484	1.84	OS	ANKRD24	-2.05	9.08E-03	0.25	1.28E-02	1.38E-38	2.04E-02	0.43
331	circR002149	-1.84	miR-7974	1.61	TG	ANKRD24	-2.05	9.08E-03	0.25	1.28E-02	1.38E-38	2.04E-02	0.43
332	circR002149	-1.84	miR-18a-3p	1.70	TG	ANKRD24	-2.05	9.08E-03	0.25	1.28E-02	1.38E-38	2.04E-02	0.43
333	circR002149	-1.84	miR-942-5p	1.58	TG	IYD	-2.47	3.21E-06	0.21	3.50E-02	7.36E-49	1.65E-03	0.35
334	circR002149	-1.84	miR-484	1.84	OS	IYD	-2.47	3.21E-06	0.21	3.50E-02	7.36E-49	1.65E-03	0.35
335	circR002149	-1.84	miR-4326	2.35	TG	IYD	-2.47	3.21E-06	0.21	3.50E-02	7.36E-49	1.65E-03	0.35
336	circR002149	-1.84	miR-330-5p	1.58	TC	IYD	-2.47	3.21E-06	0.21	3.50E-02	7.36E-49	1.65E-03	0.35
337	circR002149	-1.84	miR-877-5p	2.41	TG	IYD	-2.47	3.21E-06	0.21	3.50E-02	7.36E-49	1.65E-03	0.35
338	circR002149	-1.84	miR-500a-3p	2.26	TG	IYD	-2.47	3.21E-06	0.21	3.50E-02	7.36E-49	1.65E-03	0.35
339	circR002149	-1.84	miR-130b-3p	2.51	TG	IYD	-2.47	3.21E-06	0.21	3.50E-02	7.36E-49	1.65E-03	0.35
340	circR002149	-1.84	miR-339-5p	2.08	TG	IYD	-2.47	3.21E-06	0.21	3.50E-02	7.36E-49	1.65E-03	0.35
341	circR002149	-1.84	miR-15b-5p	1.84	TG	IYD	-2.47	3.21E-06	0.21	3.50E-02	7.36E-49	1.65E-03	0.35
342	circR002149	-1.84	miR-18a-3p	1.70	TG	IYD	-2.47	3.21E-06	0.21	3.50E-02	7.36E-49	1.65E-03	0.35
343	circR002149	-1.84	miR-941	2.03	TG	IYD	-2.47	3.21E-06	0.21	3.50E-02	7.36E-49	1.65E-03	0.35
344	circR002149	-1.84	miR-7974	1.61	TG	IYD	-2.47	3.21E-06	0.21	3.50E-02	7.36E-49	1.65E-03	0.35
345	circR002149	-1.84	miR-877-3p	1.58	TG	IYD	-2.47	3.21E-06	0.21	3.50E-02	7.36E-49	1.65E-03	0.35
346	circR002149	-1.84	miR-574-5p	1.77	TG	IYD	-2.47	3.21E-06	0.21	3.50E-02	7.36E-49	1.65E-03	0.35
347	circR002149	-1.84	miR-185-5p	1.79	TG	IYD	-2.47	3.21E-06	0.21	3.50E-02	7.36E-49	1.65E-03	0.35
348	circR002149	-1.84	miR-501-5p	2.38	TG	IYD	-2.47	3.21E-06	0.21	3.50E-02	7.36E-49	1.65E-03	0.35
349	circR000943	-2.32	miR-941	2.03	TG	IYD	-2.47	4.56E-04	0.45	3.41E-06	1.38E-38	1.80E-02	0.36
350	circR000943	-2.32	miR-339-5p	2.08	TG	IYD	-2.47	4.56E-04	0.45	3.41E-06	1.38E-38	1.80E-02	0.36
351	circR000943	-2.32	miR-1306-5p	1.54	TG	IYD	-2.47	4.56E-04	0.45	3.41E-06	1.38E-38	1.80E-02	0.36
352	circR000943	-2.32	miR-1301-3p	2.42	TG	IYD	-2.47	4.56E-04	0.45	3.41E-06	1.38E-38	1.80E-02	0.36
353	circR000943	-2.32	miR-500a-3p	2.26	TG	IYD	-2.47	4.56E-04	0.45	3.41E-06	1.38E-38	1.80E-02	0.36
354	circR000943	-2.32	miR-760	1.74	TG	IYD	-2.47	4.56E-04	0.45	3.41E-06	1.38E-38	1.80E-02	0.36
355	circR000943	-2.32	miR-574-5p	1.77	TG	IYD	-2.47	4.56E-04	0.45	3.41E-06	1.38E-38	1.80E-02	0.36
356	circR000943	-2.32	miR-221-3p	3.85	TG	IYD	-2.47	4.56E-04	0.45	3.41E-06	1.38E-38	1.80E-02	0.36
357	circR000943	-2.32	miR-501-5p	2.38	TG	IYD	-2.47	4.56E-04	0.45	3.41E-06	1.38E-38	1.80E-02	0.36
358	circR000943	-2.32	miR-185-5p	1.79	TG	IYD	-2.47	4.56E-04	0.45	3.41E-06	1.38E-38	1.80E-02	0.36
359	circR000943	-2.32	miR-330-5p	1.58	TC	IYD	-2.47	4.56E-04	0.45	3.41E-06	1.38E-38	1.80E-02	0.36
360	circR000943	-2.32	miR-7974	1.61	TG	IYD	-2.47	4.56E-04	0.45	3.41E-06	1.38E-38	1.80E-02	0.36
361	circR005131	1.82	miR-378c	-1.57		NPC1	2.10	3.01E-04	0.41	3.40E-05	1.23E-05	1.44E-02	0.45
362	circR005131	1.82	miR-4686	-8.16		NPC1	2.10	3.01E-04	0.41	3.40E-05	1.23E-05	1.44E-02	0.45
363	circR004796	1.68	miR-4686	-8.16		PLOD3	2.07	0.00E+00	0.37	1.93E-04	1.23E-05	4.50E-02	0.35
364	circR071935	1.57	miR-7704	-2.06		RIBC2	4.58	1.22E-04	0.23	2.26E-02	2.21E-05	1.38E-03	0.35
365	circR071935	1.57	miR-4686	-8.16		RIBC2	4.58	1.22E-04	0.23	2.26E-02	2.21E-05	1.38E-03	0.35

366	circR006909	-1.63	miR-7706	2.27	AK126380	-3.02	3.31E-06	0.31	1.73E-03	6.55E-55	5.14E-12	0.32
367	circR006909	-1.63	miR-224-5p	6.89	AK126380	-3.02	3.31E-06	0.31	1.73E-03	6.55E-55	5.14E-12	0.32
368	circR006909	-1.63	miR-671-5p	1.67	AK126380	-3.02	3.31E-06	0.31	1.73E-03	6.55E-55	5.14E-12	0.32
369	circR006909	-1.63	miR-130b-5p	2.73	AK126380	-3.02	3.31E-06	0.31	1.73E-03	6.55E-55	5.14E-12	0.32
370	circR006909	-1.63	miR-96-5p	4.60	MROH2A	-3.48	2.84E-04	0.37	1.53E-04	7.61E-46	2.69E-02	0.42
371	circR006909	-1.63	miR-181b-5p	1.55	MROH2A	-3.48	2.84E-04	0.37	1.53E-04	7.61E-46	2.69E-02	0.42
372	circR006909	-1.63	miR-769-5p	1.80	MROH2A	-3.48	2.84E-04	0.37	1.53E-04	7.61E-46	2.69E-02	0.42
373	circR006909	-1.63	miR-34a-5p	1.97	MROH2A	-3.48	2.84E-04	0.37	1.53E-04	7.61E-46	2.69E-02	0.42
374	circR006909	-1.63	miR-3615	1.70	CHRD	-2.22	1.77E-04	0.25	1.13E-02	2.02E-47	1.68E-03	0.34
375	circR006909	-1.63	miR-769-5p	1.80	CHRD	-2.22	1.77E-04	0.25	1.13E-02	2.02E-47	1.68E-03	0.34
376	circR006909	-1.63	miR-34a-5p	1.97	CHRD	-2.22	1.77E-04	0.25	1.13E-02	2.02E-47	1.68E-03	0.34
377	circR006909	-1.63	miR-93-3p	1.68	CHRD	-2.22	1.77E-04	0.25	1.13E-02	2.02E-47	1.68E-03	0.34
378	circR006909	-1.63	miR-221-5p	2.70	CHRD	-2.22	1.77E-04	0.25	1.13E-02	2.02E-47	1.68E-03	0.34
379	circR006909	-1.63	miR-196b-5p	7.07	HPD	-5.86	1.56E-02	0.41	3.02E-05	9.80E-17	1.57E-02	0.32
380	circR006909	-1.63	miR-671-5p	1.67	HPD	-5.86	1.56E-02	0.41	3.02E-05	9.80E-17	1.57E-02	0.32
381	circR006909	-1.63	miR-671-5p	1.67	FNDC4	-2.42	5.65E-03	0.42	2.12E-05	1.11E-27	3.20E-04	0.31
382	circR006909	-1.63	miR-1295a	2.26	ANKRD24	-2.05	3.49E-07	0.28	6.05E-03	3.71E-62	2.29E-02	0.49
383	circR006909	-1.63	miR-21-3p	2.18	ANKRD24	-2.05	3.49E-07	0.28	6.05E-03	3.71E-62	2.29E-02	0.49
384	circR006909	-1.63	miR-671-5p	1.67	ANKRD24	-2.05	3.49E-07	0.28	6.05E-03	3.71E-62	2.29E-02	0.49
385	circR006909	-1.63	miR-93-3p	1.68	ANKRD24	-2.05	3.49E-07	0.28	6.05E-03	3.71E-62	2.29E-02	0.49
386	circR006909	-1.63	miR-210-3p	1.90	ANKRD24	-2.05	3.49E-07	0.28	6.05E-03	3.71E-62	2.29E-02	0.49
387	circR006909	-1.63	miR-3615	1.70	ANKRD24	-2.05	3.49E-07	0.28	6.05E-03	3.71E-62	2.29E-02	0.49
388	circR006909	-1.63	miR-769-5p	1.80	ANKRD24	-2.05	3.49E-07	0.28	6.05E-03	3.71E-62	2.29E-02	0.49
389	circR006909	-1.63	miR-93-3p	1.68	AK126380	-3.02	3.31E-06	0.31	1.73E-03	6.55E-55	5.14E-12	0.32
390	circR006909	-1.63	miR-589-5p	2.77	AK126380	-3.02	3.31E-06	0.31	1.73E-03	6.55E-55	5.14E-12	0.32
391	circR006909	-1.63	miR-96-5p	4.60	AK126380	-3.02	3.31E-06	0.31	1.73E-03	6.55E-55	5.14E-12	0.32
392	circR002149	-1.84	miR-93-3p	1.68	ANKRD24	-2.05	9.08E-03	0.25	1.28E-02	1.38E-38	2.04E-02	0.43
393	circR002149	-1.84	miR-671-5p	1.67	ANKRD24	-2.05	9.08E-03	0.25	1.28E-02	1.38E-38	2.04E-02	0.43
394	circR002149	-1.84	miR-769-5p	1.80	ANKRD24	-2.05	9.08E-03	0.25	1.28E-02	1.38E-38	2.04E-02	0.43
395	circR002149	-1.84	miR-34a-5p	1.97	IYD	-2.47	3.21E-06	0.21	3.50E-02	7.36E-49	1.65E-03	0.35
396	circR002149	-1.84	miR-93-3p	1.68	IYD	-2.47	3.21E-06	0.21	3.50E-02	7.36E-49	1.65E-03	0.35
397	circR002149	-1.84	miR-96-5p	4.60	IYD	-2.47	3.21E-06	0.21	3.50E-02	7.36E-49	1.65E-03	0.35
398	circR002149	-1.84	miR-769-5p	1.80	IYD	-2.47	3.21E-06	0.21	3.50E-02	7.36E-49	1.65E-03	0.35
399	circR002149	-1.84	miR-221-5p	2.70	IYD	-2.47	3.21E-06	0.21	3.50E-02	7.36E-49	1.65E-03	0.35
400	circR002149	-1.84	miR-17-5p	1.95	IYD	-2.47	3.21E-06	0.21	3.50E-02	7.36E-49	1.65E-03	0.35
401	circR002149	-1.84	miR-589-5p	2.77	IYD	-2.47	3.21E-06	0.21	3.50E-02	7.36E-49	1.65E-03	0.35
402	circR000943	-2.32	miR-766-3p	1.56	IYD	-2.47	4.56E-04	0.45	3.41E-06	1.38E-38	1.80E-02	0.36
403	circR000943	-2.32	miR-17-5p	1.95	IYD	-2.47	4.56E-04	0.45	3.41E-06	1.38E-38	1.80E-02	0.36
404	circR000943	-2.32	miR-34a-5p	1.97	IYD	-2.47	4.56E-04	0.45	3.41E-06	1.38E-38	1.80E-02	0.36
405	circR000943	-2.32	miR-93-3p	1.68	IYD	-2.47	4.56E-04	0.45	3.41E-06	1.38E-38	1.80E-02	0.36
406	circR000943	-2.32	miR-769-5p	1.80	IYD	-2.47	4.56E-04	0.45	3.41E-06	1.38E-38	1.80E-02	0.36

FOOTNOTE: TS: Tumor size, TG: Tumor Grade, TC: Tumor Capsule, DC: Degree of encapsulation, VI: Vascular invasion, TI: Tumor Invasion, CS: Cancer stage, OS: Overall Survival. Red outline: Upregulate in worse clinical features. Green outline: Upregulate in better clinical features.

Table S6. Moderately confident (significant for 2 (PC and PPC) of 4 statistical tests) circRNA-miRNA-mRNA triplets.

Table S6. Moderately confident (significant for 2 (PC and PPC) of 4 statistical tests) circRNA-miRNA-mRNA triplets.

No.	circRNA			Common miRNA			Competing mRNA			ceRNA (statistics)			
	ID	FC	Clinical association	ID	FC	Clinical association	ID	FC	Clinical association	P-Value (Hypergeometric Test)	Pearson Correlation Coefficient (PC)	P-value (PC)	P-value (PPC)
1	circR091581(circGPC3)	5.01	TG	miR-378a-3p	-1.72	TG DC	TOP2A	20.25		0.00E+00	0.72	1.06E-16	1.23E-05
2	circR091581(circGPC3)	5.01	TG	miR-378a-3p	-1.72		NEK2	14.44	TG DC	0.00E+00	0.72	3.18E-17	1.23E-05
3	circR091581(circGPC3)	5.01	TG	miR-378a-3p	-1.72		ASPM	11.68	TG	0.00E+00	0.73	2.56E-17	1.23E-05
4	circR091581(circGPC3)	5.01	TG	miR-378a-3p	-1.72		CENPW	4.66	TG	0.00E+00	0.72	1.15E-16	1.23E-05
5	circR091581(circGPC3)	5.01	TG	miR-378a-3p	-1.72		GPC3	27.21	TG	0.00E+00	0.88	6.85E-33	1.23E-05
6	circR091581(circGPC3)	5.01	TG	miR-378a-3p	-1.72		DBF4	2.05	TG	0.00E+00	0.70	5.49E-16	1.23E-05
7	circR091581(circGPC3)	5.01	TG	miR-378a-3p	-1.72		KIF14	6.87	TG	0.00E+00	0.72	5.45E-17	1.23E-05
8	circR091581(circGPC3)	5.01	TG	miR-378a-3p	-1.72		ERCC6L	14.52	TG	0.00E+00	0.70	8.23E-16	1.23E-05
9	circR091581(circGPC3)	5.01	TG	miR-378a-3p	-1.72		MMS22L	3.15	TG	0.00E+00	0.72	1.06E-16	1.23E-05
10	circR091581(circGPC3)	5.01	TG	miR-378a-3p	-1.72		POLQ	9.67	TG	0.00E+00	0.70	5.95E-16	1.23E-05
11	circR091581(circGPC3)	5.01	TG	miR-378a-3p	-1.72		E2F7	14.41	TG	0.00E+00	0.71	3.08E-16	1.23E-05
12	circR091581(circGPC3)	5.01	TG	miR-378c	-1.57	TG DC	TOP2A	20.25	TG	0.00E+00	0.72	1.06E-16	1.23E-05
13	circR091581(circGPC3)	5.01	TG	miR-378c	-1.57	TG DC	NEK2	14.44	TG	0.00E+00	0.72	3.18E-17	1.23E-05
14	circR091581(circGPC3)	5.01	TG	miR-378c	-1.57		CENPW	4.66	TG	0.00E+00	0.72	1.15E-16	1.23E-05
15	circR091581(circGPC3)	5.01	TG	miR-378c	-1.57		GPC3	27.21	TG	0.00E+00	0.88	6.85E-33	1.23E-05
16	circR091581(circGPC3)	5.01	TG	miR-378c	-1.57		DBF4	2.05	TG	0.00E+00	0.70	5.49E-16	1.23E-05
17	circR091581(circGPC3)	5.01	TG	miR-378c	-1.57		KIF14	6.87	TG	0.00E+00	0.72	5.45E-17	1.23E-05
18	circR091581(circGPC3)	5.01	TG	miR-378c	-1.57		ERCC6L	14.52	TG	0.00E+00	0.70	8.23E-16	1.23E-05
19	circR091581(circGPC3)	5.01	TG	miR-378c	-1.57		ASPM	11.68	TG	0.00E+00	0.73	2.56E-17	1.23E-05
20	circR091581(circGPC3)	5.01	TG	miR-378c	-1.57		MMS22L	3.15	TG	0.00E+00	0.72	1.06E-16	1.23E-05
21	circR091581(circGPC3)	5.01	TG	miR-378c	-1.57		POLQ	9.67	TG	0.00E+00	0.70	5.95E-16	1.23E-05
22	circR091581(circGPC3)	5.01	TG	miR-378c	-1.57		E2F7	14.41	TG	0.00E+00	0.71	3.08E-16	1.23E-05
23	circR034537	-1.73	TS	miR-4326	2.35	TS TG VI	THBS1	-2.17		0.00E+00	0.77	4.52E-20	3.17E-11
24	circR034537	-1.73	TS	miR-500a-3p	2.26	TS TG VI	THBS1	-2.17		0.00E+00	0.77	4.52E-20	3.17E-11
25	circR034537	-1.73	TS	miR-769-5p	1.80	TS TG VI	THBS1	-2.17		0.00E+00	0.77	4.52E-20	3.17E-11
26	circR034537	-1.73	TS	miR-130b-5p	2.73	TS TG VI	THBS1	-2.17		0.00E+00	0.77	4.52E-20	3.17E-11
27	circR034537	-1.73	TS	miR-186-5p	1.57	TS TG VI	THBS1	-2.17		0.00E+00	0.77	4.52E-20	3.17E-11
28	circR025332	-2.69	TC	miR-361-5p	1.60	TG	C1R	-2.79		0.00E+00	0.72	5.68E-17	1.62E-07
29	circR025332	-2.69	TC	miR-216a-3p	5.20	TI	C1R	-2.79		0.00E+00	0.72	5.68E-17	1.62E-07
30	circR025332	-2.69	TC	miR-671-5p	1.67		C1R	-2.79		0.00E+00	0.72	5.68E-17	1.62E-07
31	circR075320	2.34	TG OS	miR-7704	-2.06		SQSTM1	2.00	TG OS	0.00E+00	0.80	2.81E-23	1.23E-05
32	circR075320	2.34	TG OS	miR-139-3p	-2.18		SQSTM1	2.00	TG OS	0.00E+00	0.80	2.81E-23	1.23E-05
33	circR035435	-1.82	TS	miR-339-5p	2.08	TG	ACADS	-2.82		3.93E-03	0.72	6.99E-17	1.23E-05
34	circR035435	-1.82	TS	miR-330-5p	1.58	TC	ACADS	-2.82		3.93E-03	0.72	6.99E-17	1.23E-05

FOOTNOTE: TS: Tumor size, TG: Tumor Grade, TC: Tumor Capsule, DC: Degree of encapsulation, VI: Vascular invasion, TI: Tumor Invasion, CS: Cancer stage, OS: Overall Survival. Red outline: Upregulate in worse clinical features. Green outline: Upregulate in better clinical features.

Table S7. Genes deregulated by circGPC3.

No.	GeneName	LO2- circGPC3 overexpression		Huh7- circGPC3 knockdown	
		FDR	FC	FDR	FC
1	MICU1(NM_001363513)	7.31E-167	4875.83	6.24E-43	-1161.17
2	GFM1(NM_001374361)	1.83E-151	4416.43	2.22E-61	-1673.03
3	ANKFY1(NR_047571)	2.26E-121	3524.05	3.85E-59	-1610.98
4	AMMECR1L	1.52E-110	3203.25	3.38E-90	-2476.91
5	MAPK9(NM_001364607)	3.51E-98	2840.05	4.34E-103	-2839.26
6	VMP1	2.13E-97	2816.98	1.36E-14	-376.11
7	RALGAPB	9.05E-87	2504.61	1.03E-68	-1876.99
8	KCTD2	9.03E-82	2357.81	4.21E-47	-1276.96
9	CLASP2	7.98E-79	2271.50	1.02E-24	-2.79
10	RMND5B	2.03E-77	2230.26	1.25E-24	-2.53
11	RAI14	3.51E-72	2076.86	3.96E-10	-3.00
12	HERC4	7.61E-72	2066.93	2.09E-18	-481.92
13	MTHFD1L	3.74E-71	2046.57	5.47E-60	-1634.49
14	NUP205	1.02E-67	1945.94	6.00E-26	-690.76
15	EZH1(NM_001321082)	1.75E-57	1647.26	2.06E-31	-842.05
16	CDC42BPA(NM_001366010)	6.70E-57	1630.17	1.87E-91	-2512.45
17	PEG10	4.61E-55	1576.40	1.31E-14	-2.78
18	CNOT4(NM_001190850)	5.14E-51	1458.88	2.40E-23	-618.69
19	CACNB3	4.97E-48	1371.53	1.17E-20	-544.41
20	ZDBF2	7.03E-46	1308.77	3.83E-59	-1611.07
21	PPIE(NR_036544)	6.21E-45	1281.09	2.15E-21	-564.70
22	NUDT22(NM_001128613)	6.97E-45	1279.62	1.35E-35	-957.87
23	XRCC3	2.35E-44	1264.17	4.11E-35	-3.32
24	KANSL3	1.34E-43	1242.15	7.82E-15	-4.98
25	SLMAP	1.11E-40	1157.47	1.76E-36	-982.36
26	CASP8AP2	3.77E-40	1142.03	9.74E-14	-4.59
27	TCOF1	4.23E-39	1111.56	9.91E-112	-3081.53
28	PAM(NM_001364585)	1.52E-38	1095.40	2.42E-54	-1477.87
29	CKAP2L	1.28E-37	1068.47	6.15E-71	-1939.05
30	CEP78	2.26E-35	1002.99	4.93E-44	-1191.93
31	FZD6	2.21E-34	974.09	1.23E-12	-2.20
32	CCDC120(NM_001163323)	2.43E-33	943.70	1.71E-15	-401.16
33	RGS14	1.12E-32	924.50	1.65E-12	-318.27
34	KIAA0895L(NM_001369682)	2.46E-32	914.56	1.83E-19	-511.26
35	ARHGAP12	1.09E-31	895.74	5.20E-04	-2.43
36	PPIP5K2	2.34E-31	886.10	1.57E-15	-3.28
37	PTPA	5.11E-31	876.22	2.12E-18	-481.71
38	MFSD11	2.19E-30	857.86	9.84E-11	-268.96
39	LARP4	7.27E-30	842.75	3.57E-15	-392.28
40	DMTN(NM_001323384)	9.97E-30	838.76	4.38E-79	-2165.88
41	C21orf62(NM_001162496)	1.10E-29	837.47	1.53E-04	-2.12
42	FGFR1(NM_015850)	4.85E-29	818.85	8.23E-65	-1768.43
43	CPEB2(NM_001177384)	7.72E-29	812.99	3.35E-28	-753.08
44	CTNND1	1.24E-28	807.02	3.53E-09	-225.64
45	MBNL3	1.41E-28	805.40	3.46E-12	-2.16
46	PWWP2A	2.47E-28	798.30	1.54E-30	-817.84
47	EFR3A	3.55E-28	793.73	1.27E-99	-2741.75
48	PRDM10(NM_001367896)	2.22E-27	770.68	1.80E-42	-1148.43
49	KAT5(NM_006388)	2.94E-27	767.16	2.50E-23	-5.90
50	ZNF41(NM_001324141)	3.71E-27	764.23	7.35E-43	-1159.20
51	PUM2	1.46E-26	747.01	4.32E-10	-251.04
52	CASP2	1.99E-26	743.03	8.63E-41	-1101.88
53	ZNF761(NM_001289952)	3.21E-26	736.97	2.20E-47	-1284.71
54	FHDC1(NM_001371116)	7.28E-26	726.55	8.25E-16	-5.93
55	ZNF37A	1.80E-25	715.12	6.60E-87	-2384.39
56	BRD2	2.53E-25	710.80	3.80E-36	-973.13
57	USP53	5.68E-25	700.59	2.29E-88	-2425.53
58	KIAA1191	7.18E-25	697.66	1.06E-13	-351.44
59	ODF2	7.61E-25	696.91	1.11E-25	-683.32
60	CITED2	8.32E-25	695.76	6.76E-28	-744.66

61	GSTCD	2.72E-24	680.79	3.65E-64	-1750.46
62	PTP4A2	3.92E-24	676.20	1.97E-24	-6.01
63	TMCC1(NM_001017395)	4.03E-24	675.83	1.21E-09	-238.60
64	CXorf38	4.43E-24	674.63	1.53E-22	-596.47
65	DNPEP	7.20E-24	668.53	4.04E-05	-2.25
66	DCAF5	1.47E-23	659.50	1.33E-55	-14.70
67	SYBU	5.92E-23	641.90	7.95E-19	-493.57
68	MECOM	6.94E-23	639.86	5.58E-09	-220.12
69	ZSCAN12(NR_160527)	9.87E-23	635.36	2.03E-18	-482.24
70	ZFAND5	1.41E-22	630.87	3.60E-13	-336.65
71	TTC23	1.54E-22	629.78	6.92E-07	-2.18
72	PCGF5	2.29E-22	624.73	8.11E-11	-271.33
73	CPEB3(NM_014912)	4.53E-22	616.18	2.27E-09	-231.01
74	NME1-NME2(NM_001018136)	9.75E-22	606.59	1.66E-59	-8.55
75	LMLN(NM_001136049)	1.77E-21	599.07	1.89E-22	-593.88
76	NRM	1.93E-21	597.98	2.52E-20	-535.12
77	METTL8(NM_001321155)	6.36E-21	582.84	1.99E-26	-704.07
78	HLCS	1.07E-20	576.31	1.12E-51	-1403.97
79	ZNF148	4.94E-20	557.01	1.39E-105	-2909.07
80	CASP3	5.69E-20	555.23	8.02E-28	-742.58
81	KIAA0753	9.12E-20	549.32	1.72E-16	-428.84
82	EMSY(NM_001300944)	1.24E-19	545.42	2.42E-09	-230.24
83	MEF2A	1.27E-19	545.09	1.69E-11	-290.25
84	DBN1	1.28E-19	545.01	8.01E-32	-853.37
85	MALT1	1.44E-19	543.48	7.71E-24	-632.36
86	CYBC1(NR_036518)	1.52E-19	542.77	5.76E-14	-358.79
87	SMG5	2.29E-19	537.62	1.64E-12	-2.74
88	ZNF432	2.91E-19	534.67	6.75E-25	-661.64
89	DERL1	3.22E-19	533.38	6.15E-06	-2.56
90	ZFAT	5.15E-19	527.42	1.45E-19	-2.81
91	PEX5	6.24E-19	524.99	3.39E-06	-3.56
92	TVP23C-CDRT4	7.90E-19	521.98	3.12E-32	-864.70
93	SMARCA2(NM_139045)	1.50E-18	513.87	1.21E-06	-3.95
94	PRELID3A(NM_001142406)	2.96E-18	505.31	2.79E-12	-3.18
95	IL32	4.69E-18	499.53	1.36E-08	-3.60
96	CAMK2G(NR_160047)	7.22E-18	494.07	2.18E-10	-259.35
97	CDC42SE2	9.34E-18	490.80	1.44E-09	-2.72
98	ZNF155(NM_003445)	1.63E-17	483.74	4.36E-02	-2.17
99	TIMM23B-AGAP6	2.96E-17	476.29	2.25E-15	-397.85
100	FRS2	3.41E-17	474.50	2.47E-17	-452.19
101	CEP83	1.19E-16	458.55	2.26E-14	-369.99
102	PHACTR4	1.27E-16	457.68	1.18E-05	-2.10
103	TNS1	1.57E-16	454.97	2.26E-29	-785.60
104	SETDB2(NM_001160308)	1.75E-16	453.60	1.20E-05	-2.33
105	ZNF391(NM_001322293)	3.01E-16	446.76	4.10E-22	-584.58
106	HBS1L	3.37E-16	445.36	5.89E-10	-247.29
107	PCM1(NM_001352649)	3.37E-16	445.35	5.81E-118	-3256.22
108	ADD1	3.73E-16	444.06	2.53E-88	-2424.28
109	MTUS1	7.47E-16	435.24	1.66E-13	-4.91
110	CYP20A1	1.15E-15	429.83	1.23E-04	-3.74
111	R3HCC1L	1.26E-15	428.65	1.82E-04	-3.14
112	SET	1.31E-15	428.13	1.36E-02	-2.07
113	ENTPD6(NM_001322388)	1.57E-15	425.80	3.05E-17	-449.63
114	ZNF197	1.62E-15	425.44	2.95E-11	-3.47
115	POT1	1.95E-15	423.09	2.35E-05	-4.50
116	LAMA3(NM_001127717)	2.91E-15	418.00	2.37E-11	-286.21
117	MYD88	3.52E-15	415.62	1.00E-07	-3.33
118	SLC35E3	4.65E-15	412.08	4.26E-35	-944.03
119	FBXL5	4.77E-15	411.76	1.74E-10	-262.08
120	ZNF30	6.06E-15	408.76	5.53E-04	-3.72

121	USP19(NM_001351105)	1.00E-14	402.31	1.71E-22	-595.06
122	ZNF124	1.04E-14	401.84	1.19E-11	-294.59
123	SWT1(NM_001105518)	1.14E-14	400.59	9.35E-37	-990.05
124	ALG1	1.66E-14	395.90	1.78E-03	-3.75
125	A2M	2.27E-14	391.99	3.26E-21	-2.31
126	MEIS2(NM_172316)	2.61E-14	390.21	3.11E-13	-3.99
127	KDM5C	3.00E-14	388.49	1.11E-08	-211.86
128	TMUB2(NM_001353176)	5.72E-14	380.33	4.66E-10	-250.12
129	FOXN2(NM_002158)	6.94E-14	377.89	8.24E-12	-298.97
130	USP3	8.32E-14	375.58	8.64E-09	-6.32
131	MTHFSD	8.88E-14	374.74	3.78E-14	-2.56
132	GDPGP1(NM_001013657)	2.11E-13	363.78	1.05E-04	-2.14
133	HCN3	2.26E-13	362.91	5.71E-10	-2.75
134	FANCD2	2.57E-13	361.25	8.21E-26	-687.01
135	BIVM	3.85E-13	356.19	1.58E-29	-789.94
136	DYSF(NM_001130986)	3.97E-13	355.80	1.37E-09	-2.06
137	PBXIP1	4.70E-13	353.66	1.11E-28	-766.33
138	MAGT1	6.75E-13	349.05	2.48E-32	-20.05
139	TCF7L2(NM_001198531)	7.96E-13	346.95	6.65E-27	-717.22
140	TANC1	8.93E-13	345.45	1.89E-16	-427.66
141	LRRC20	9.80E-13	344.28	9.24E-61	-1655.93
142	KDM4C	1.31E-12	340.64	4.58E-15	-389.26
143	INTS7	1.54E-12	338.54	4.77E-11	-277.76
144	GSN	1.74E-12	337.00	1.73E-10	-3.58
145	ZNF33A	1.95E-12	335.54	3.26E-28	-753.43
146	SYNJ1	2.58E-12	331.99	8.55E-10	-242.78
147	ATRX	4.04E-12	326.32	1.25E-70	-9.12
148	ZNF75A	4.14E-12	326.00	1.85E-20	-538.84
149	FECH	5.14E-12	323.24	3.52E-05	-5.76
150	BBS9(NM_001033604)	1.47E-11	309.96	2.82E-08	-200.53
151	RASA4B	1.52E-11	309.54	1.65E-07	-3.38
152	ZNF227(NM_001289166)	1.84E-11	307.10	8.29E-07	-2.14
153	RCBTB2	2.54E-11	303.06	3.41E-07	-4.02
154	TLK2	4.59E-11	295.60	4.03E-09	-224.06
155	CHEK1	4.71E-11	295.27	2.54E-04	-4.59
156	ZGRF1	5.90E-11	292.42	1.97E-12	-316.18
157	DTNB(NM_001256304)	6.56E-11	291.06	7.69E-31	-826.19
158	MAPK7	8.38E-11	287.93	3.54E-15	-392.36
159	ARIH2	1.05E-10	285.05	8.92E-78	-2129.39
160	AASDH(NM_181806)	1.08E-10	284.67	3.65E-26	-696.74
161	KLHL24(NM_001349425)	1.47E-10	280.82	6.33E-24	-634.74
162	C2orf27A	1.51E-10	280.50	2.38E-12	-313.91
163	BAIAP3	1.67E-10	279.18	3.94E-09	-224.31
164	TBC1D5	1.88E-10	277.64	2.83E-87	-2394.73
165	ZNF518A	2.06E-10	276.55	1.39E-17	-459.12
166	SETD5	2.12E-10	276.15	1.48E-59	-1622.56
167	STAG2	2.38E-10	274.69	3.84E-12	-308.17
168	NUMB	2.38E-10	274.65	4.09E-28	-750.67
169	ZNF429(NM_001346916)	3.58E-10	269.53	1.43E-45	-1234.67
170	CRAT	3.67E-10	269.18	3.08E-08	-8.24
171	NUDCD2	3.90E-10	268.39	1.06E-04	-3.12
172	ALDH6A1(NM_001278593)	4.17E-10	267.56	2.45E-09	-230.08
173	MAP3K3	4.58E-10	266.35	5.43E-11	-5.15
174	RPUSD1(NM_001324410)	7.24E-10	260.59	1.02E-44	-1210.99
175	ZBED3	8.05E-10	259.23	1.96E-03	-2.35
176	LYPLA1(NM_001279359)	8.55E-10	258.47	3.32E-17	-3.16
177	CLGN	8.56E-10	258.45	1.60E-03	-3.26
178	ZDHHC23	8.70E-10	258.25	9.48E-14	-2.26
179	RAB11FIP2	9.28E-10	257.43	4.51E-15	-389.44
180	KIF23	9.37E-10	257.32	1.04E-08	-7.32

181	TIA1	1.01E-09	256.31	5.37E-13	-331.82
182	MINK1	1.42E-09	251.95	4.40E-04	-4.09
183	ERC1	1.63E-09	250.21	3.05E-44	-1197.68
184	ASCC2(NM_001369933)	1.67E-09	249.89	8.20E-04	-2.15
185	SLC30A6	2.76E-09	243.48	8.12E-06	-4.18
186	UPF3A	3.32E-09	241.18	2.72E-10	-256.67
187	NRCAM	4.32E-09	237.83	2.86E-25	-3.67
188	RTN3	4.66E-09	236.89	1.48E-12	-319.61
189	ITGA7(NM_001144997)	4.92E-09	236.20	9.24E-07	-2.52
190	NUBPL	5.23E-09	235.40	1.75E-05	-2.10
191	ZNF254	1.07E-08	226.35	1.60E-09	-235.20
192	API5	1.13E-08	225.62	3.35E-17	-448.49
193	CEBPA	1.16E-08	225.25	1.11E-49	-8.36
194	TMEM135	1.53E-08	221.71	2.23E-08	-2.61
195	FGD3	1.56E-08	221.51	9.52E-11	-269.38
196	CFAP97D1	1.63E-08	220.93	4.10E-02	-2.10
197	SLC25A45(NM_182556)	3.06E-08	213.00	8.23E-04	-4.07
198	SLC7A2	3.27E-08	212.19	2.13E-307	-8705.33
199	PLPP1(NM_176895)	3.96E-08	209.71	4.93E-03	-2.51
200	DCAF1	4.22E-08	208.91	2.67E-13	-340.25
201	SCMH1	5.23E-08	206.21	4.65E-12	-305.89
202	MSTO1(NM_001350784)	5.59E-08	205.34	5.71E-32	-857.41
203	VEZT	5.81E-08	204.82	1.07E-30	-822.22
204	CORO7	6.62E-08	203.17	4.85E-14	-360.86
205	LDAH(NM_001282723)	7.05E-08	202.36	1.25E-25	-681.88
206	CYB5RL	7.30E-08	201.91	1.39E-18	-486.82
207	GEMIN5	8.62E-08	199.79	9.19E-15	-3.53
208	AKNA(NM_030767)	9.76E-08	198.18	2.59E-07	-2.67
209	FBXL20	1.34E-07	194.05	1.21E-10	-266.47
210	ABI2(NR_164708)	1.35E-07	194.02	7.91E-18	-465.95
211	ADPRH	1.37E-07	193.83	6.51E-23	-606.69
212	DTNA	1.42E-07	193.34	1.97E-62	-1702.22
213	UEVLD	1.42E-07	193.31	1.26E-23	-626.46
214	PLCD1	1.45E-07	193.06	1.13E-06	-3.24
215	SCARB1	2.28E-07	187.22	3.55E-03	-2.43
216	KANK1	2.35E-07	186.83	5.13E-30	-803.41
217	THRB	2.80E-07	184.55	3.24E-17	-448.92
218	ZBTB17(NM_001287604)	3.02E-07	183.56	1.13E-11	-295.12
219	ZC3H11A(NM_001376341)	4.14E-07	179.57	1.52E-08	-208.02
220	GMPR2(NM_001351024)	4.58E-07	178.23	7.53E-13	-327.75
221	PWWP3A(NM_001369796)	6.42E-07	173.88	2.55E-17	-451.76
222	TGOLN2	8.36E-07	170.54	1.87E-04	-2.56
223	THRA	8.97E-07	169.66	8.12E-03	-2.63
224	ARNT(NM_001350224)	1.14E-06	166.61	1.27E-10	-265.92
225	ABCB8(NM_001282292)	1.30E-06	164.93	1.04E-16	-10.30
226	FAM122B	1.54E-06	162.82	1.17E-09	-238.97
227	CBWD5	1.58E-06	162.53	5.19E-05	-3.16
228	PPP1R3B	1.84E-06	160.59	2.13E-35	-952.34
229	PHTF1	1.98E-06	159.68	8.79E-11	-8.14
230	ARMC10	2.17E-06	158.52	1.06E-15	-2.09
231	SCFD1	2.18E-06	158.44	5.19E-20	-5.63
232	DNAJC10	2.43E-06	157.08	3.89E-04	-4.38
233	RARA	2.65E-06	155.97	8.45E-06	-4.27
234	FAM219B	2.69E-06	155.81	2.36E-06	-5.49
235	FBXL22	2.76E-06	155.49	1.71E-09	-234.42
236	HSF4	2.93E-06	154.71	8.50E-13	-326.29
237	ASB8	3.03E-06	154.32	6.78E-06	-6.32
238	ZNF789	3.63E-06	152.03	3.10E-10	-3.21
239	GULP1	3.67E-06	151.88	5.59E-11	-2.48
240	CTNNA1	4.30E-17	14.09	3.70E-11	-280.79

241	TLE4(NM_001351563)	4.20E-60	13.53	9.60E-14	-352.65
242	RWDD2A	1.14E-07	11.98	1.92E-02	-2.45
243	SLC41A2	2.99E-44	11.76	4.15E-22	-584.41
244	NRBF2	1.12E-13	11.40	2.26E-04	-2.10
245	CUL4A	1.61E-23	11.21	1.09E-02	-2.30
246	ARMCX5	3.39E-13	9.96	2.48E-02	-2.07
247	LRCH3	2.18E-21	9.79	5.42E-18	-470.49
248	TMEM128	2.61E-04	9.14	1.01E-05	-8.39
249	NFXL1	8.14E-15	9.11	1.09E-02	-2.67
250	DTX2	1.44E-10	9.07	2.41E-03	-3.31
251	FAM49B	1.55E-07	9.03	2.84E-06	-2.13
252	RC3H2	7.52E-54	9.00	3.12E-43	-1169.59
253	ZNF260	4.51E-28	8.27	3.57E-09	-4.75
254	LINC00673	9.77E-06	8.21	2.68E-05	-2.25
255	TIGD6	9.27E-07	8.18	4.02E-08	-3.61
256	C1orf43	9.87E-08	7.94	1.08E-12	-4.08
257	ABTB1	2.88E-20	7.80	7.03E-14	-356.39
258	CNOT7	4.29E-16	7.40	1.11E-04	-2.27
259	SUPT20H	7.45E-11	7.30	2.26E-04	-3.32
260	WDR20(NM_001330228)	6.68E-09	7.25	2.97E-12	-311.25
261	NT5C2(NM_001351184)	5.52E-07	7.10	3.16E-10	-254.85
262	RBBP4	4.46E-12	6.83	7.96E-04	-3.01
263	AKAP10	8.14E-09	6.75	1.72E-05	-4.60
264	TNFAIP3	1.75E-27	6.72	3.60E-08	-2.25
265	RPS6KB1	7.02E-14	6.67	7.48E-09	-4.19
266	DNMT3B	9.64E-08	6.64	1.39E-22	-597.58
267	TSHZ2(NM_173485)	2.72E-35	6.42	1.12E-12	-323.01
268	MADD(NR_164838)	1.38E-09	6.25	1.16E-06	-2.78
269	DENND1A(NM_001352966)	8.15E-11	6.19	5.88E-06	-3.63
270	DDX19A(NM_001320527)	2.04E-04	6.12	1.39E-05	-4.15
271	DDX47	9.53E-07	6.02	3.34E-15	-393.08
272	LONP2	9.61E-33	6.00	5.96E-12	-302.89
273	POLR2B	2.72E-08	5.97	4.51E-08	-3.72
274	STAT3	6.35E-66	5.96	8.62E-11	-2.15
275	ZNF704(NM_001367783)	1.24E-06	5.84	2.21E-26	-19.94
276	UNKL	2.08E-05	5.78	4.17E-10	-4.64
277	LZIC	6.60E-05	5.69	9.11E-09	-9.49
278	MRPS31P5	1.16E-04	5.66	4.41E-03	-2.33
279	DMTF1	3.08E-16	5.57	2.08E-08	-204.21
280	CCDC77	1.10E-12	5.55	3.21E-02	-2.29
281	TATDN3	2.74E-09	5.47	1.93E-03	-2.92
282	RAD51	1.16E-03	5.46	4.25E-03	-3.12
283	SNHG3	7.95E-04	5.45	1.89E-12	-7.11
284	UCK1	3.77E-09	5.45	1.53E-04	-3.11
285	BCL2L1	9.51E-06	5.44	2.69E-03	-2.15
286	SNX6	8.40E-13	5.42	8.61E-14	-10.80
287	SMPD1(NM_001365135)	6.99E-06	5.42	1.07E-05	-2.57
288	REEP1	4.88E-08	5.40	3.54E-13	-336.85
289	MIGA1	2.11E-12	5.32	1.12E-14	-4.34
290	DAG1(NM_001177644)	3.54E-07	5.19	2.51E-29	-784.35
291	SHPRH	1.66E-08	5.03	2.26E-16	-425.51
292	CAP1	5.00E-08	5.02	1.51E-04	-2.48
293	ZNF619	1.81E-05	5.02	3.04E-13	-338.68
294	MLEC	2.76E-04	5.00	8.90E-16	-408.98
295	PKNOX1	1.31E-05	4.90	2.05E-26	-703.71
296	ADAR	3.20E-22	4.90	3.08E-06	-3.70
297	MARCHF6	1.22E-08	4.84	1.06E-18	-490.10
298	P2RY11	2.47E-03	4.82	6.17E-34	-911.83
299	KCTD18(NM_152387)	1.33E-06	4.80	1.63E-05	-2.30
300	CNOT2	2.71E-16	4.80	2.14E-07	-2.77

301	HMGCS1(NM_001324219)	4.38E-24	4.70	1.03E-03	-2.08
302	SEPTIN2	2.12E-04	4.68	5.65E-09	-219.97
303	TRIB3	1.42E-87	4.65	7.47E-85	-2327.07
304	MKLN1	3.85E-07	4.47	2.61E-08	-4.21
305	AGAP3	4.56E-07	4.43	6.61E-11	-5.90
306	SLC35F5	5.19E-12	4.23	7.36E-09	-2.19
307	TAB2	9.11E-32	4.21	5.46E-82	-2247.02
308	CELF1	1.02E-05	4.17	1.44E-04	-2.17
309	TRABD(NM_001320488)	2.40E-10	4.16	5.98E-13	-18.12
310	USP15	1.64E-08	4.15	1.06E-09	-240.22
311	PEX2	8.40E-13	4.14	1.88E-10	-2.45
312	MIOS	4.00E-04	4.12	1.77E-28	-4.20
313	KLHL28	4.33E-04	4.08	1.58E-09	-235.40
314	PSME3	6.81E-04	4.04	3.01E-03	-3.52
315	SRSF11	1.35E-34	4.04	9.77E-30	-2.81
316	SOCS2(NM_001270468)	4.49E-07	4.01	1.68E-04	-4.92
317	GAPDH	6.94E-03	4.01	2.64E-03	-2.40
318	WDR6	3.10E-42	3.98	9.01E-77	-2101.49
319	FBLIM1	5.77E-04	3.95	2.29E-14	-11.50
320	RSRP1(NR_135790)	4.61E-06	3.92	1.61E-15	-401.84
321	NAT9	6.58E-03	3.91	5.07E-04	-4.87
322	TOR1AIP2(NM_145034)	3.77E-39	3.90	1.89E-23	-2.17
323	HDAC11	4.95E-03	3.90	5.21E-11	-2.03
324	ATP6V1H	1.36E-08	3.88	1.81E-06	-2.14
325	KLHL8	2.56E-12	3.85	3.30E-10	-2.16
326	LSM12	7.08E-12	3.81	1.40E-03	-2.27
327	LPGAT1	3.64E-23	3.80	3.20E-11	-4.49
328	TXNDC11	4.92E-04	3.79	5.56E-09	-2.23
329	LNPK	4.56E-08	3.74	1.75E-19	-511.80
330	ZNF559	1.61E-06	3.73	2.99E-04	-2.71
331	PPP1R9A(NM_001166161)	1.05E-03	3.69	8.84E-09	-214.56
332	NFIA	2.22E-05	3.55	3.31E-15	-393.16
333	MTMR2	1.54E-05	3.54	1.02E-14	-6.68
334	CLOCK	7.05E-12	3.50	7.77E-10	-243.94
335	DMPK(NM_001288765)	3.31E-12	3.47	2.54E-05	-2.68
336	ATL2	5.15E-03	3.40	3.99E-09	-224.16
337	IST1	3.44E-32	3.39	6.87E-174	-9.98
338	CHRAC1	1.02E-05	3.39	6.80E-04	-2.03
339	PCMTD2	4.77E-11	3.39	1.53E-04	-2.10
340	FBXO28	4.30E-08	3.35	1.36E-08	-209.33
341	KIAA1958	1.27E-06	3.33	1.48E-23	-624.49
342	PPP2R5E	8.16E-10	3.31	8.67E-32	-4.28
343	REC8	1.63E-10	3.30	8.06E-09	-215.68
344	RBM41(NM_001171080)	1.05E-10	3.28	7.49E-29	-771.17
345	BTRC	3.21E-04	3.26	1.72E-02	-2.44
346	STK40	2.84E-04	3.21	1.69E-18	-484.49
347	MCFD2	1.90E-03	3.21	1.22E-16	-7.38
348	ITSN1	1.08E-09	3.20	9.55E-44	-1183.98
349	ERLIN2	1.57E-02	3.19	5.93E-08	-3.40
350	STK36(NM_015690)	1.48E-10	3.18	8.97E-82	-2240.97
351	OSBPL3(NM_145321)	3.04E-09	3.17	3.59E-39	-1056.91
352	GAR1	7.91E-39	3.16	7.31E-21	-2.72
353	CSNK1G3	3.31E-05	3.15	2.11E-16	-426.30
354	TACC1(NM_001352803)	1.48E-11	3.07	1.83E-133	-3692.45
355	ZUP1	1.17E-04	3.06	3.15E-13	-338.26
356	ZNF841(NM_001369828)	9.05E-03	3.05	3.52E-12	-309.22
357	PTPDC1	1.85E-05	3.04	3.77E-04	-2.54
358	CD46(NM_172358)	6.45E-03	3.04	4.75E-10	-2.19
359	DHRS4-AS1	6.86E-04	3.02	8.50E-05	-2.14
360	ZEB1	4.62E-04	3.01	8.17E-09	-215.52

361	C18orf54	4.65E-04	2.99	2.04E-24	-648.26
362	EDRF1	4.64E-03	2.98	1.55E-11	-7.20
363	AFTPH	1.68E-06	2.98	1.09E-15	-406.60
364	SLC35A3	1.77E-05	2.96	2.98E-11	-3.04
365	MANEAL	6.09E-05	2.91	6.23E-09	-218.79
366	TMEM263	1.57E-03	2.91	1.82E-02	-2.01
367	IVD	5.93E-03	2.88	8.41E-10	-3.61
368	SIRT2	3.71E-03	2.87	1.14E-20	-4.17
369	SMUG1	5.96E-04	2.84	6.64E-09	-218.00
370	SASH1	2.54E-05	2.79	4.34E-07	-2.11
371	NCAPG	3.32E-03	2.79	1.28E-06	-3.15
372	PPARA	6.87E-03	2.77	4.41E-10	-2.56
373	TSC2	2.11E-26	2.76	1.32E-53	-1457.39
374	PAPLN(NR_158678)	3.76E-02	2.75	1.18E-19	-516.53
375	PLEC	8.76E-65	2.74	1.04E-26	-4.34
376	OGDHL	1.27E-08	2.74	1.46E-17	-458.52
377	ZNF114	8.40E-13	2.73	3.57E-06	-3.55
378	ZFYVE27	2.08E-06	2.70	1.03E-16	-3.72
379	PNISR	2.38E-20	2.69	2.77E-16	-2.28
380	SPATA33	2.58E-02	2.66	1.66E-05	-2.71
381	ISG20L2	5.99E-08	2.66	1.15E-06	-2.51
382	PALM	9.01E-06	2.64	2.77E-32	-2.69
383	PGAP2	3.04E-02	2.63	2.75E-10	-15.28
384	HNRNPA1L2	1.14E-06	2.63	1.76E-06	-2.15
385	RHOBTB1	5.58E-03	2.63	2.09E-13	-9.52
386	GTF2H3	1.72E-10	2.63	2.83E-11	-284.04
387	MAP2K6	4.48E-03	2.61	1.35E-04	-2.51
388	PAQR4	1.61E-10	2.55	2.79E-06	-7.31
389	JMJD1C	7.54E-14	2.53	2.39E-14	-369.32
390	ZSCAN32	1.13E-02	2.53	1.09E-08	-212.07
391	SDAD1	1.05E-11	2.51	3.10E-08	-2.35
392	GRB10(NM_001350814)	9.38E-03	2.50	6.58E-06	-2.69
393	ASPM	2.38E-02	2.49	1.39E-17	-2.23
394	KIF20B	8.13E-05	2.47	2.68E-10	-4.19
395	ZDHHC13	2.05E-05	2.47	2.49E-12	-313.36
396	ANO9	1.38E-02	2.45	1.20E-15	-10.72
397	ANKRD17	1.15E-02	2.45	5.69E-06	-4.47
398	NDUFA10	2.43E-06	2.44	1.55E-04	-3.60
399	INMT(NM_006774)	2.93E-03	2.42	2.91E-11	-283.71
400	VPS52	1.66E-04	2.42	1.24E-04	-2.54
401	AMIGO2	1.11E-16	2.41	1.56E-14	-8.08
402	ABLIM1	3.74E-05	2.40	6.74E-17	-2.42
403	NPL(NM_030769)	1.48E-06	2.40	2.12E-09	-231.85
404	PPP6R2	2.51E-02	2.38	1.57E-03	-2.08
405	SRP54	2.54E-06	2.36	2.26E-13	-5.79
406	RNF145	5.59E-10	2.35	1.63E-10	-262.87
407	CEP97	2.74E-11	2.34	6.12E-21	-552.14
408	SLIT2(NM_004787)	2.28E-06	2.34	2.21E-18	-5.88
409	RABL2B	5.40E-03	2.31	5.64E-04	-2.17
410	KDM1A	1.05E-04	2.31	1.01E-39	-18.37
411	KIAA0040	8.33E-11	2.31	3.26E-03	-2.17
412	PLAGL1(NM_001080956)	5.73E-03	2.30	1.35E-06	-4.89
413	HP1BP3	4.17E-02	2.29	1.72E-04	-2.82
414	GORASP1	6.53E-06	2.28	2.30E-07	-2.24
415	NDOR1	8.05E-09	2.27	7.60E-07	-2.17
416	SZT2	4.52E-06	2.27	3.16E-49	-3.85
417	CARD8	3.83E-03	2.26	9.87E-03	-2.30
418	UCHL5	1.41E-05	2.26	2.33E-09	-230.69
419	RIF1	1.35E-04	2.26	5.56E-11	-6.09
420	ERF	3.37E-04	2.25	7.25E-48	-6.98

421	PUS7L(NM_031292)	1.29E-16	2.25	1.47E-18	-4.75
422	KLRD1	4.72E-02	2.24	1.05E-08	-212.43
423	UBXN2B	1.27E-03	2.24	1.70E-06	-3.81
424	RNF138	4.87E-05	2.23	1.35E-10	-3.45
425	FAM185A(NM_001145268)	1.82E-02	2.22	2.81E-11	-5.83
426	GPBP1	2.42E-02	2.22	4.02E-07	-3.81
427	ARHGAP17	5.38E-07	2.17	6.39E-07	-2.20
428	DCTN1	2.48E-17	2.16	4.22E-26	-2.00
429	ELP5	7.86E-17	2.16	9.03E-48	-1295.47
430	RHOT2(NR_147954)	9.44E-06	2.15	3.92E-11	-280.10
431	RNF146	2.21E-02	2.15	6.58E-17	-440.40
432	PPP1R13L	2.90E-08	2.15	3.61E-08	-3.56
433	HNRNPA3	7.56E-17	2.14	4.19E-99	-4.29
434	RWDD4	1.95E-03	2.14	6.58E-17	-11.89
435	ZNF266(NM_006631)	3.90E-03	2.14	3.62E-02	-2.18
436	WDR13	1.23E-05	2.14	3.13E-15	-3.23
437	UFM1	4.23E-02	2.13	7.56E-09	-12.57
438	ST3GAL3	3.11E-02	2.13	5.82E-09	-219.62
439	CAPZB	1.58E-05	2.13	6.03E-03	-2.05
440	VARS2	3.52E-12	2.12	8.53E-14	-2.15
441	BUD13	2.29E-03	2.12	2.48E-04	-2.52
442	MCM3	1.17E-08	2.11	1.56E-08	-3.16
443	TMEM259	5.91E-07	2.10	2.32E-13	-11.88
444	MECP2	1.60E-02	2.10	3.54E-12	-309.16
445	ZFP90	4.00E-04	2.08	3.55E-16	-420.05
446	ATG16L1	5.45E-05	2.07	5.36E-10	-2.22
447	NDUFAF7(NM_001350027)	2.58E-02	2.07	7.06E-06	-4.01
448	AK3	2.35E-06	2.07	1.39E-13	-2.77
449	TTC17	1.89E-07	2.07	1.65E-85	-2345.34
450	UBE2L3	1.71E-02	2.06	6.14E-04	-2.02
451	TMEM51	1.18E-05	2.06	3.64E-12	-2.48
452	MKS1	2.25E-03	2.05	2.07E-11	-287.85
453	PPIL3	4.79E-02	2.05	2.00E-08	-2.84
454	CDK5RAP2	2.98E-22	2.05	3.50E-73	-2001.52
455	RRN3	1.31E-20	2.05	3.47E-16	-2.05
456	DPH5	1.11E-02	2.05	2.72E-05	-2.98
457	EVI5(NM_001308248)	1.89E-07	2.05	2.39E-19	-2.64
458	HNRNPH3	2.03E-16	2.04	1.41E-36	-985.08
459	LINC01089	2.50E-02	2.04	2.17E-05	-3.86
460	HPS5	5.87E-12	2.03	7.71E-16	-2.64
461	MAPK3	2.94E-02	2.03	2.22E-08	-2.63
462	TOP3B(NM_001349848)	2.19E-03	2.02	8.02E-08	-2.47
463	GATA2	8.11E-11	2.02	2.77E-18	-3.57
464	HDLBP	7.86E-06	2.02	8.38E-03	-2.13
465	VDAC2	3.12E-06	2.02	3.54E-10	-253.48
466	TPMT	3.79E-03	2.01	1.67E-06	-8.92
467	CFLAR	4.97E-02	2.00	4.90E-19	-499.39
1	MICU1(NM_006077)	5.03E-216	-5653.64	3.67E-60	1576.62
2	JAK1	1.88E-168	-4388.20	4.05E-49	3.24
3	GOLGB1	1.64E-139	-3621.03	2.86E-27	699.78
4	ITPRID2	8.60E-120	-3102.76	2.98E-29	752.59
5	TJP2	3.35E-109	-2823.56	2.51E-66	1741.08
6	TLE4(NM_001351546)	7.42E-95	-2448.39	6.75E-31	796.35
7	ALDH18A1	3.01E-92	-2380.48	6.94E-23	582.97
8	CDC42BPA(NM_003607)	7.85E-88	-2264.85	3.39E-56	1470.97
9	FGFR1(NM_023105)	3.85E-84	-2167.93	4.52E-74	1948.58
10	GAPVD1	2.17E-82	-2122.43	1.64E-44	1159.31
11	GFM1(NM_001374358)	3.39E-82	-2117.31	1.07E-101	2691.87
12	MID1IP1	1.30E-64	-1657.94	3.86E-156	4163.70
13	TCF12	3.30E-62	-1595.38	1.23E-37	976.00
14	MEGF8	3.96E-58	-1489.25	1.35E-66	4.42
15	NUDT22(NM_001271831)	5.18E-57	-1460.05	3.30E-26	671.50

16	CNOT4(NM_001190847)	7.50E-57	-1455.84	1.83E-43	1131.23
17	FOXN2(NM_001375444)	1.68E-55	-1420.73	2.19E-47	1235.80
18	KNOP1	4.45E-53	-1357.68	4.27E-14	8.35
19	ERCC6L2	5.19E-46	-1173.65	6.41E-11	263.83
20	BEND3	1.49E-45	-1161.69	5.48E-41	1065.21
21	ANKFY1(NM_016376)	2.48E-44	-1129.72	4.26E-149	3972.33
22	DMPK(NM_001081562)	3.97E-44	-1124.43	6.85E-12	289.86
23	ZNF431	7.79E-44	-1116.82	2.22E-35	915.64
24	DYSF(NM_001130983)	2.82E-40	-1024.60	3.88E-25	642.95
25	TSHZ2(NM_001193421)	1.16E-39	-1008.61	5.60E-13	318.80
26	APC	2.44E-38	-974.30	1.21E-13	336.67
27	DPP8	4.14E-38	-968.22	1.06E-18	3.62
28	CHAMP1	1.07E-35	-905.47	1.97E-12	2.84
29	ZNF33B	9.97E-33	-828.22	1.38E-08	201.27
30	DPYSL5	1.12E-32	-826.93	6.23E-100	2644.34
31	KIAA0895L(NM_001369685)	1.28E-32	-825.41	3.52E-11	270.80
32	ENTPD6(NM_001322396)	3.94E-31	-786.74	1.87E-119	3170.71
33	EVI5(NM_005665)	1.96E-30	-768.63	1.08E-41	1084.06
34	ZNF655	3.17E-30	-763.22	4.58E-16	401.24
35	ZNF668	4.98E-30	-758.16	1.02E-29	765.06
36	PLAGL1(NM_006718)	7.13E-30	-754.12	2.59E-10	2.52
37	C21orf62(NM_019596)	1.03E-29	-749.96	2.64E-15	7.38
38	GTF2IP4	1.18E-29	-748.39	2.05E-69	1823.70
39	C17orf80	2.44E-29	-740.21	2.85E-10	246.42
40	PDE4A	4.73E-29	-732.80	3.23E-07	2.97
41	ZNF155(NM_198089)	8.78E-29	-725.79	1.12E-14	4.42
42	KLHL24(NM_017644)	3.42E-28	-710.51	3.59E-34	883.45
43	ZNF41(NM_001324139)	6.75E-28	-702.84	8.23E-44	1140.63
44	CASP10	4.05E-27	-682.72	2.95E-22	3.01
45	DAG1(NM_001177638)	7.35E-27	-676.06	4.12E-14	349.09
46	LMLN(NR_026786)	1.19E-26	-670.63	2.00E-20	517.54
47	SUMF2	4.02E-26	-656.78	4.12E-20	11.60
48	BBS9(NM_198428)	6.30E-26	-651.71	1.12E-20	524.29
49	PUS7L(NM_001271826)	6.72E-26	-650.96	6.19E-24	2.28
50	RBM41(NM_001324244)	3.76E-25	-631.46	1.13E-37	976.98
51	PPIE(NM_001319293)	6.39E-25	-625.49	5.65E-18	452.20
52	PRDM10(NM_001367895)	8.33E-25	-622.47	1.09E-41	7.64
53	ZNF689	1.23E-24	-618.08	1.40E-24	9.46
54	LMBR1	4.82E-24	-602.66	1.31E-21	2.38
55	ANKLE1	1.69E-23	-588.55	3.67E-08	5.85
56	EMSY(NM_001300942)	1.91E-23	-587.12	2.59E-26	674.31
57	MAPK9(NM_001364608)	2.37E-23	-584.68	6.12E-79	2078.77
58	SLC25A39	4.16E-23	-578.33	4.51E-31	4.31
59	LDAH(NM_001282722)	5.29E-23	-575.61	1.60E-40	1052.74
60	USP7	7.58E-23	-571.50	0.00E+00	9540.78
61	SRPK2	7.64E-23	-571.40	4.90E-11	266.99
62	RNF24	1.13E-22	-566.97	6.66E-08	4.12
63	ERBB2	1.08E-21	-541.57	1.18E-08	2.00
64	JUP	2.61E-21	-531.63	1.26E-02	2.41
65	CBLB	2.81E-21	-530.80	1.14E-33	870.02
66	ARHGAP29	8.50E-21	-518.29	8.75E-67	1753.44
67	TRPM7	2.29E-20	-507.11	3.80E-89	2353.26
68	ZNF841(NM_001369830)	2.83E-20	-504.73	5.64E-18	452.22
69	AASDH(NM_001286670)	3.69E-20	-501.72	2.62E-06	3.53
70	PRXL2B	5.50E-20	-497.24	1.08E-10	257.78
71	PAPLN(NM_001365906)	8.15E-20	-492.83	2.60E-53	1393.89
72	NME1-NME2(NR_037149)	8.88E-20	-491.87	2.69E-83	2195.58
73	TACC1(NM_001352804)	9.80E-20	-490.75	1.98E-16	410.95
74	SLIT2(NM_001289136)	1.01E-19	-490.39	1.10E-91	2421.91
75	FAM126B	1.06E-19	-489.84	2.55E-27	701.10

76	LAMA3(NM_198129)	1.15E-19	-488.89	3.55E-16	404.18
77	DOP1B	1.38E-19	-486.85	3.95E-32	829.13
78	FAM156A	1.39E-19	-486.75	1.96E-43	1130.46
79	TMUB2(NR_148390)	1.47E-19	-486.09	1.29E-26	682.38
80	BARD1	1.91E-19	-483.19	1.68E-69	1826.08
81	PTOV1	2.03E-19	-482.50	6.34E-162	4320.80
82	ZNF227(NM_001289169)	3.81E-19	-475.41	2.77E-06	3.50
83	NPL(NM_001200052)	1.19E-18	-462.54	2.22E-03	2.95
84	ZNF286A	1.46E-18	-460.19	7.26E-61	1595.37
85	PRAG1	3.80E-18	-449.43	9.03E-19	4.02
86	OCRL	3.96E-18	-448.99	1.10E-79	2098.93
87	MADD(NM_001376654)	4.26E-18	-448.14	6.40E-13	5.19
88	USP20	5.70E-18	-444.82	1.48E-31	813.91
89	WDR48	6.20E-18	-443.88	8.66E-14	340.55
90	MPDZ	6.21E-18	-443.85	7.83E-40	1034.37
91	OSBPL3(NM_145320)	6.49E-18	-443.36	5.45E-60	1572.05
92	ZNF429(NM_001001415)	7.41E-18	-441.86	7.76E-26	3.62
93	FHDC1(NM_033393)	8.98E-18	-439.69	1.75E-16	3.14
94	NCOA1	1.23E-17	-436.13	1.44E-03	2.10
95	B9D1	1.52E-17	-433.76	3.26E-11	271.70
96	PCM1(NM_001352655)	1.54E-17	-433.57	3.28E-20	511.78
97	SOCS2(NM_001270471)	2.53E-17	-427.98	4.27E-09	214.94
98	PANK1	5.41E-17	-419.43	5.72E-11	8.15
99	MYADM	6.38E-17	-417.52	3.30E-20	511.70
100	TMCC1(NM_001349264)	3.99E-16	-396.69	6.45E-38	983.42
101	ABCD4	4.91E-16	-394.30	1.02E-49	1298.24
102	GTF2I	5.60E-16	-392.82	2.73E-08	5.16
103	DTNB(NM_001256308)	6.98E-16	-390.33	1.07E-19	498.19
104	ZBTB8A	1.64E-15	-380.64	8.13E-12	287.86
105	ZNF567	2.30E-15	-376.77	1.21E-08	202.87
106	NIF3L1	4.54E-15	-369.12	1.85E-09	224.65
107	CREB1	8.48E-15	-362.01	1.59E-49	1293.01
108	MPHOSPH9	8.85E-15	-361.54	3.98E-22	562.78
109	ALDH6A1(NM_001278594)	9.36E-15	-360.89	3.07E-03	2.39
110	HELLS	1.07E-14	-359.31	8.87E-05	2.35
111	CD99L2	1.34E-14	-356.74	8.44E-05	3.82
112	DENND1A(NM_001352967)	1.94E-14	-352.59	1.57E-10	6.07
113	ZNF266(NM_001370384)	4.55E-14	-343.01	2.43E-04	2.75
114	GDPGP1(NM_001322811)	7.20E-14	-337.82	3.79E-14	12.11
115	ZNF704(NM_001033723)	8.18E-14	-336.36	3.12E-22	2.38
116	GMPR2(NM_001283022)	8.37E-14	-336.10	5.19E-05	2.10
117	ZSCAN12(NR_133668)	1.09E-13	-333.10	9.63E-09	4.38
118	UBASH3B	1.22E-13	-331.85	1.46E-16	414.54
119	RASSF5	1.23E-13	-331.72	4.06E-14	2.57
120	PRKAG2	2.47E-13	-323.86	2.04E-25	650.38
121	LYRM1	3.36E-13	-320.37	3.12E-08	7.89
122	ABI2(NR_164705)	4.88E-13	-316.14	2.12E-09	223.13
123	VOPP1	9.59E-13	-308.43	1.30E-08	201.96
124	ZCCHC7	1.18E-12	-306.02	4.02E-06	3.79
125	GRB10(NM_001001549)	1.57E-12	-302.85	2.23E-58	1529.12
126	AUH	1.71E-12	-301.88	1.85E-12	305.01
127	CCDC138	2.08E-12	-299.63	1.20E-03	3.25
128	MEN1	2.75E-12	-296.48	3.83E-15	376.62
129	ATG2A	6.56E-12	-286.60	1.71E-08	198.78
130	PLPP1(NR_103485)	6.81E-12	-286.17	2.38E-09	4.89
131	SLC25A16	9.09E-12	-282.89	6.09E-20	504.65
132	PRELID3A(NM_001142405)	1.38E-11	-278.16	1.34E-32	841.63
133	CIITA	1.39E-11	-278.07	2.81E-03	2.41
134	YY1AP1	2.18E-11	-273.03	4.77E-10	240.42
135	GRK6	3.13E-11	-268.94	7.66E-11	261.78

136	AP1G2	4.84E-11	-264.02	6.12E-19	477.95
137	TWF1	5.71E-11	-262.15	7.87E-15	368.27
138	ZNF343	5.91E-11	-261.74	4.13E-21	535.75
139	MTRR	5.94E-11	-261.70	2.75E-11	273.69
140	GPI	7.92E-11	-258.44	2.83E-16	406.79
141	FIP1L1	8.41E-11	-257.73	4.56E-09	214.17
142	PPP1R9A(NM_017650)	1.00E-10	-255.71	3.11E-21	539.04
143	TUG1	1.62E-10	-250.28	1.25E-10	6.75
144	METTL8(NM_001321160)	1.65E-10	-250.06	7.65E-30	768.32
145	ASCC2(NM_001369922)	1.74E-10	-249.43	2.10E-04	2.37
146	TAF1C	2.25E-10	-246.56	4.35E-06	2.20
147	ZNF565	3.59E-10	-241.26	7.55E-09	208.32
148	SEMA4F	3.99E-10	-240.04	2.41E-43	1128.10
149	DACT2	5.18E-10	-237.08	1.39E-73	1935.47
150	ADGRG1	5.99E-10	-235.43	3.66E-10	243.53
151	LIG1	7.18E-10	-233.40	4.20E-41	1068.29
152	EFCAB11	8.80E-10	-231.08	1.49E-05	4.14
153	ZNF613	9.48E-10	-230.23	1.14E-03	3.28
154	MEF2C	1.05E-09	-229.02	7.64E-09	208.17
155	NDUFS1	1.30E-09	-226.67	1.22E-05	2.64
156	DPF1	1.81E-09	-222.85	4.10E-11	269.02
157	REV1	2.12E-09	-221.07	8.53E-21	527.40
158	KIAA0895	2.27E-09	-220.24	1.99E-03	2.08
159	LOC102724788	2.70E-09	-218.25	3.45E-24	617.65
160	COPS3	3.42E-09	-215.61	1.10E-06	8.12
161	ASTE1	4.78E-09	-211.80	1.36E-05	5.10
162	LYPLA1(NM_001279356)	6.45E-09	-208.41	3.28E-03	2.29
163	C19orf54	8.43E-09	-205.35	1.15E-12	310.43
164	PWWP3A(NM_001369797)	9.96E-09	-203.44	7.77E-64	1674.74
165	TBC1D2B	1.05E-08	-202.83	2.19E-15	383.10
166	CACNA1G	1.12E-08	-202.06	4.91E-12	293.71
167	DOCK1	1.83E-08	-196.53	1.80E-03	2.13
168	SMARCA2(NM_001289397)	1.91E-08	-196.04	3.65E-13	323.76
169	INMT(NM_001199219)	1.91E-08	-196.01	1.71E-13	332.64
170	CTNS	2.18E-08	-194.53	3.44E-11	271.07
171	TRMU	2.31E-08	-193.87	1.21E-16	416.74
172	SNX13	2.45E-08	-193.23	1.82E-11	278.47
173	YBEY	5.17E-08	-184.78	7.12E-05	2.73
174	MYB	5.62E-08	-183.79	1.14E-16	417.40
175	WIPI1	5.63E-08	-183.77	4.59E-08	8.49
176	CNOT1	6.18E-08	-182.72	2.11E-05	3.19
177	HPS4	6.92E-08	-181.42	4.99E-18	453.65
178	QRICH1	8.44E-08	-179.15	1.24E-08	202.58
179	SPATS2L	8.59E-08	-178.95	2.22E-20	516.31
180	STK36(NM_001243313)	1.02E-07	-177.00	1.54E-21	6.03
181	SUCO	1.04E-07	-176.69	2.06E-91	2414.45
182	OARD1	1.12E-07	-175.93	2.27E-09	222.28
183	KIAA0586	1.14E-07	-175.63	1.31E-15	389.03
184	SLC44A3	2.10E-07	-168.57	3.04E-18	459.35
185	TOGARAM2	2.39E-07	-167.12	1.82E-10	251.67
186	FGD4	2.74E-07	-165.51	1.75E-06	2.16
187	TRIM6	3.24E-07	-163.61	5.35E-43	1118.75
188	TJAP1	3.55E-07	-162.55	1.75E-03	2.12
189	PBX3	3.63E-07	-162.30	7.19E-21	3.92
190	WDR20(NM_001353674)	4.71E-07	-159.30	1.78E-02	2.02
191	CAMK2G(NM_001367537)	5.92E-07	-156.66	3.64E-15	377.21
192	FANCC	6.30E-07	-155.95	1.12E-11	284.12
193	CYP4F3	7.79E-07	-153.56	1.09E-28	737.58
194	AKAP12	8.73E-07	-152.24	2.01E-07	3.78
195	EME1	1.03E-12	-16.21	4.99E-03	2.37

196	HIC1	5.31E-08	-13.27	6.33E-06	3.12
197	NDUFS2	9.65E-11	-12.79	1.06E-33	31.55
198	FOXRED1	1.24E-07	-11.81	1.86E-04	2.78
199	MYBL1	9.21E-18	-10.67	3.87E-06	2.97
200	ERBIN	1.46E-10	-10.60	1.19E-04	2.61
201	EBAG9	1.19E-07	-9.78	4.62E-06	3.90
202	ABHD3	5.92E-06	-9.10	1.18E-02	2.41
203	ENOSF1	6.97E-05	-8.64	2.89E-14	353.21
204	SLC35G1	2.69E-09	-8.61	3.25E-05	2.42
205	MED20	1.67E-06	-8.18	1.34E-08	4.05
206	NRP1	7.04E-11	-8.11	7.39E-15	9.33
207	RPA1	6.79E-11	-8.09	2.23E-04	2.05
208	TRABD(NR_135275)	6.38E-07	-7.94	3.09E-07	8.18
209	ZNF623	1.59E-27	-7.53	2.00E-33	863.58
210	MON2	1.12E-21	-7.40	1.00E-15	392.21
211	METTL6	5.06E-08	-7.25	1.75E-07	3.51
212	KMT5B	1.67E-16	-7.18	8.60E-23	10.81
213	DPH7	2.62E-11	-7.18	2.32E-15	382.44
214	FASTKD2	2.99E-07	-6.83	2.38E-05	4.72
215	ZNF544	5.13E-12	-6.64	4.12E-05	3.55
216	ATR	2.34E-21	-6.58	1.55E-30	8.09
217	CHEK2	1.62E-12	-6.52	5.28E-03	2.45
218	XPO5	1.13E-07	-6.48	2.08E-28	730.06
219	HJURP	1.00E-07	-6.38	1.81E-09	224.92
220	GJC1	5.16E-24	-6.18	3.40E-57	1497.47
221	MAP1B	1.67E-18	-6.16	2.59E-06	3.09
222	SLC25A45(NM_001352382)	3.88E-09	-6.11	3.37E-03	2.76
223	TOP3B(NR_146277)	2.57E-08	-6.01	2.33E-17	3.82
224	EFTUD2	6.08E-40	-5.77	2.65E-16	407.58
225	SMPD1(NM_001318087)	7.55E-04	-5.71	4.61E-05	3.67
226	IARS1	8.55E-156	-5.60	1.37E-29	761.62
227	UBE2H	5.60E-04	-5.46	6.12E-04	2.46
228	CNN2	1.16E-03	-5.42	2.14E-14	5.43
229	OGA	3.03E-36	-5.35	2.24E-23	3.56
230	SMIM11A	4.41E-28	-5.22	5.04E-05	2.08
231	CAP2	2.54E-10	-5.21	3.02E-09	6.64
232	POLK	6.54E-17	-5.18	2.00E-64	1690.37
233	FAM222B	8.29E-05	-5.13	5.79E-10	2.31
234	THOC5	1.82E-07	-5.13	4.49E-15	3.22
235	DENND2B	5.63E-08	-5.12	4.53E-13	321.29
236	CCDC120(NM_033626)	7.80E-11	-5.07	5.75E-99	2618.13
237	MFAP3	7.36E-04	-4.89	5.82E-08	7.15
238	PMS2	2.44E-13	-4.88	2.53E-05	2.60
239	DMTN(NM_001323385)	1.67E-20	-4.84	2.79E-130	3462.82
240	TMF1	2.13E-13	-4.72	1.27E-08	202.21
241	FAM239A	5.72E-08	-4.60	4.01E-11	4.45
242	TPM4	1.42E-11	-4.59	6.99E-04	2.13
243	C19orf48	5.10E-12	-4.59	4.33E-05	5.41
244	KCTD20	2.80E-07	-4.57	2.17E-11	2.86
245	CRELD2	1.23E-03	-4.48	3.33E-05	3.45
246	CCDC163	2.27E-05	-4.46	9.20E-06	5.38
247	ABHD18	3.13E-08	-4.45	2.43E-03	2.04
248	TOR1AIP2(NM_001349937)	4.78E-10	-4.44	6.01E-23	584.66
249	ZGPAT	2.12E-09	-4.41	6.77E-03	2.13
250	FAM214B	9.02E-04	-4.38	9.08E-04	2.02
251	POLA1	4.98E-42	-4.27	7.73E-175	4672.49
252	G2E3	2.66E-04	-4.24	5.05E-20	2.82
253	SRRM1	3.53E-10	-4.22	8.99E-17	420.14
254	ZC3H11A(NM_001350263)	7.86E-14	-4.21	2.57E-37	14.03
255	USP47	1.39E-09	-4.19	1.29E-26	682.42

256	PMS1	7.76E-08	-4.16	3.12E-14	2.29
257	RPS6KA5	4.60E-15	-4.13	9.79E-15	365.74
258	ZNF566	1.11E-05	-4.13	1.24E-26	682.89
259	TRIP10	1.37E-09	-4.09	5.16E-10	3.48
260	ARID4B	5.03E-04	-4.07	6.02E-05	4.21
261	ARL6	2.43E-03	-4.03	1.77E-08	198.41
262	CCM2	4.40E-05	-4.02	1.04E-03	2.08
263	SUPT20HL2	2.93E-03	-4.00	2.50E-05	2.29
264	ARSA	3.62E-05	-3.97	5.53E-07	6.85
265	TMEM183A	3.31E-07	-3.92	8.08E-29	7.59
266	C2orf42	2.10E-14	-3.91	3.76E-15	376.84
267	CD46(NM_172351)	5.68E-20	-3.86	8.61E-72	3.04
268	GAA	7.61E-10	-3.83	1.84E-18	2.32
269	DDX21	3.63E-11	-3.83	3.59E-04	2.08
270	UBAP1	4.33E-08	-3.82	4.36E-09	6.31
271	TMCO4	1.20E-08	-3.80	1.22E-08	3.36
272	RHOT2(NR_147956)	1.26E-07	-3.78	5.38E-16	399.38
273	ETF1	6.33E-10	-3.75	3.69E-20	510.43
274	BTBD7	2.22E-12	-3.73	1.11E-11	2.83
275	RIC8A	5.61E-10	-3.62	7.14E-06	3.54
276	SETDB2(NM_001320699)	1.38E-15	-3.61	1.02E-21	551.89
277	ZNF761(NM_001289951)	7.96E-14	-3.58	3.20E-17	2.25
278	CNOT8	3.54E-08	-3.54	1.89E-09	3.40
279	AKNA(NM_001317950)	5.44E-03	-3.53	9.36E-04	2.39
280	CPNE7	1.55E-08	-3.49	1.12E-17	444.25
281	CBFB	3.13E-29	-3.47	1.16E-25	2.00
282	TCF7L2(NM_001146283)	7.47E-05	-3.46	1.43E-14	4.44
283	SWT1(NM_017673)	1.56E-03	-3.37	2.12E-38	996.22
284	ABCB9	1.74E-07	-3.32	4.53E-03	2.55
285	ABCB8(NM_001282293)	7.07E-04	-3.29	2.51E-06	3.20
286	TXNRD2	4.75E-04	-3.27	7.83E-03	2.72
287	SFXN1	6.89E-03	-3.24	2.24E-08	195.63
288	THAP5	5.77E-03	-3.21	7.03E-10	4.92
289	PTDSS2	2.44E-05	-3.20	4.05E-05	2.01
290	ST3GAL4	1.58E-07	-3.19	9.39E-09	205.78
291	RNF38	3.94E-06	-3.19	5.94E-14	12.05
292	ORC3	2.68E-14	-3.16	6.01E-05	3.37
293	NDUFAF7(NR_146409)	1.34E-04	-3.14	1.46E-03	2.53
294	DENND4A	1.40E-03	-3.13	2.59E-22	567.76
295	ITGA7(NM_002206)	1.49E-07	-3.10	8.85E-15	366.91
296	PIP5K1A	9.93E-13	-3.09	3.16E-08	5.56
297	EI24	1.19E-02	-3.09	8.36E-10	233.90
298	WWTR1	4.40E-05	-3.07	5.36E-20	6.64
299	MSH5	2.84E-05	-3.03	4.13E-12	13.15
300	WDR25	2.41E-06	-3.02	1.53E-05	3.73
301	GORASP2	8.94E-07	-3.02	7.78E-11	5.61
302	KDELR2	8.38E-03	-3.02	5.15E-06	3.32
303	SUSD1	2.62E-04	-3.01	2.33E-10	3.46
304	CYBC1(NM_001193655)	3.55E-05	-3.00	2.29E-07	2.18
305	AKT1S1	4.88E-04	-2.99	5.55E-08	4.74
306	CNEP1R1	6.12E-07	-2.97	1.51E-06	4.24
307	TSC1	4.89E-05	-2.95	4.73E-09	213.76
308	ZBTB17(NM_001324138)	4.28E-16	-2.91	1.26E-04	3.10
309	MTDH	1.23E-12	-2.89	1.12E-25	657.38
310	TBL1XR1	9.36E-24	-2.89	1.46E-46	1213.90
311	HMCES	1.20E-02	-2.87	1.39E-03	2.61
312	SENP5	1.49E-14	-2.87	2.65E-10	5.55
313	CIZ1	5.06E-09	-2.84	1.92E-16	3.16
314	ACTR3B	4.76E-03	-2.83	5.71E-37	958.15
315	NUDT1	1.34E-02	-2.81	1.97E-03	2.06

316	DHX8	9.83E-22	-2.78	1.97E-29	9.91
317	DOHH	3.57E-03	-2.78	1.23E-09	12.18
318	PI4KB	1.58E-18	-2.76	2.19E-41	1075.88
319	DENND1B	1.33E-04	-2.76	5.71E-23	585.25
320	MINDY3	4.96E-06	-2.75	1.51E-03	2.03
321	DGKA	5.57E-04	-2.74	2.31E-13	329.06
322	RNF41	7.40E-04	-2.72	1.04E-28	738.09
323	ZBTB25	9.68E-04	-2.69	1.71E-08	198.79
324	CLTA	9.73E-06	-2.68	1.30E-30	788.74
325	ZFP30	2.80E-03	-2.68	3.23E-10	6.43
326	VPS37A	1.54E-02	-2.67	1.79E-10	9.96
327	RAD17	2.11E-03	-2.65	3.50E-05	2.28
328	CPEB2(NM_182485)	5.60E-05	-2.64	8.38E-26	10.52
329	AHDC1	3.65E-17	-2.62	9.49E-09	2.19
330	TTC3	2.92E-04	-2.62	2.62E-48	1260.46
331	DEDD2	1.45E-03	-2.61	4.29E-06	6.26
332	SGSH	2.89E-03	-2.61	5.66E-03	2.25
333	RBM39	1.84E-08	-2.60	8.55E-06	2.38
334	ALS2CL	1.62E-06	-2.60	6.87E-09	2.36
335	ZNF391(NM_001076781)	2.69E-04	-2.58	1.22E-10	4.42
336	L3MBTL3	5.03E-03	-2.57	1.10E-02	2.52
337	PIBF1	2.22E-02	-2.56	1.76E-03	2.42
338	BAZ2B	4.31E-05	-2.55	2.55E-13	3.63
339	LSM4	1.82E-03	-2.54	4.23E-04	2.26
340	USP19(NM_001351107)	1.30E-18	-2.53	2.17E-56	5.05
341	RPUSD1(NM_001324413)	1.40E-24	-2.53	3.08E-22	565.73
342	ATP2C1	2.30E-06	-2.52	4.39E-07	2.37
343	CLIP2	1.37E-04	-2.52	1.79E-11	3.79
344	ANKRD11	2.07E-68	-2.51	1.94E-90	2.42
345	NSMCE2	3.48E-02	-2.50	1.55E-02	2.23
346	XPO4	4.36E-06	-2.50	3.18E-13	3.04
347	C2CD2	6.66E-09	-2.50	1.24E-10	2.41
348	VPS11	8.57E-12	-2.50	2.68E-10	2.54
349	EZH1(NM_001991)	7.77E-19	-2.49	1.93E-23	4.02
350	ZNF692	1.80E-04	-2.49	4.31E-04	2.40
351	PRPF4	2.75E-28	-2.47	1.96E-28	2.53
352	ALDH2	4.89E-02	-2.47	6.00E-04	2.07
353	BCS1L	2.52E-05	-2.47	8.02E-11	2.49
354	ARNT(NM_001286035)	1.30E-04	-2.44	1.14E-03	2.52
355	FAM185A(NR_146989)	2.94E-03	-2.41	5.77E-06	8.06
356	ALDH3A2	1.06E-03	-2.40	5.18E-09	212.70
357	BSG	1.57E-15	-2.40	6.16E-07	2.27
358	DUS4L	7.13E-06	-2.39	2.55E-08	4.94
359	BCL2L11	3.13E-08	-2.38	2.16E-15	4.00
360	RBM34	9.42E-03	-2.36	8.91E-03	2.33
361	GIGYF1	1.18E-05	-2.36	2.32E-09	2.02
362	NCF2	4.79E-05	-2.31	1.47E-07	3.01
363	SNX11	1.62E-04	-2.30	1.53E-05	3.82
364	TTC9C	7.11E-05	-2.30	4.80E-17	12.19
365	DDX19A(NM_001320525)	6.50E-04	-2.29	2.08E-19	11.17
366	WSB2	1.92E-03	-2.27	5.29E-09	2.88
367	NSRP1	2.23E-02	-2.27	8.97E-10	2.77
368	MSTO1(NM_001350779)	4.33E-03	-2.26	1.08E-31	817.53
369	AURKB	1.00E-02	-2.26	3.79E-06	2.19
370	HMGCS1(NM_001330663)	1.35E-03	-2.26	6.30E-16	8.47
371	RINT1	1.42E-02	-2.24	1.22E-04	4.74
372	ZNF136	4.95E-03	-2.21	9.08E-07	2.64
373	DOK4	7.51E-06	-2.21	1.15E-28	2.57
374	BACH1	4.71E-04	-2.20	2.20E-05	2.45
375	KCTD18(NM_001321547)	8.91E-04	-2.17	9.08E-15	2.46

376	ZNF410	2.21E-03	-2.17	4.65E-06	3.20
377	TP53BP1	1.97E-07	-2.16	1.07E-13	338.07
378	CPEB3(NM_001178137)	3.41E-05	-2.13	2.38E-12	302.08
379	HSP90AB1	3.15E-07	-2.13	7.58E-20	4.88
380	TCERG1	2.09E-04	-2.12	3.19E-55	6.49
381	FIGNL1	5.65E-12	-2.12	4.21E-25	2.71
382	RSRP1(NR_135793)	4.81E-04	-2.10	1.59E-09	226.41
383	OSBPL2	1.04E-02	-2.09	2.84E-16	406.78
384	VPS45	2.67E-04	-2.09	4.21E-03	2.58
385	KAT5(NM_182710)	2.40E-10	-2.09	7.82E-55	1434.51
386	NT5C2(NM_001351181)	3.12E-02	-2.09	4.79E-16	400.71
387	COX18	3.71E-02	-2.08	6.58E-05	2.71
388	MDM1	7.88E-03	-2.08	2.17E-06	2.14
389	CTNNB1	8.69E-04	-2.07	1.12E-10	2.25
390	MARCHF7	1.05E-03	-2.07	5.58E-11	6.82
391	MUTYH	1.32E-02	-2.07	1.43E-08	3.85
392	SH3GLB1	5.38E-03	-2.07	9.49E-10	3.73
393	MIGA2	1.27E-03	-2.07	4.69E-10	2.54
394	CNOT6L	1.00E-04	-2.07	4.45E-05	2.82
395	TOP2B	1.02E-12	-2.06	3.37E-17	2.19
396	WASHC2C	5.04E-06	-2.06	3.63E-12	9.24
397	PAM(NM_001364593)	1.39E-09	-2.06	3.56E-63	1656.95
398	ORC2	2.87E-02	-2.06	5.56E-05	2.10
399	RPL10	6.86E-04	-2.05	2.32E-10	4.64
400	SH3GL1	3.72E-03	-2.05	3.25E-10	2.30
401	MEIS2(NM_002399)	1.17E-03	-2.05	6.46E-14	2.78
402	PHB	1.17E-02	-2.04	1.82E-04	3.62
403	BTBD3	4.87E-02	-2.04	4.48E-03	2.43
404	SNHG17	8.86E-03	-2.03	1.75E-05	4.04
405	ENSA	6.44E-09	-2.03	3.38E-33	857.57
406	RNF34	1.65E-05	-2.03	1.39E-07	2.13
407	UBE2E1	8.97E-03	-2.02	1.92E-04	2.15
408	CXCL16	2.16E-02	-2.01	2.05E-03	2.56
409	ADAMTSL5	1.95E-04	-2.01	5.42E-10	7.50

Table S8. Genes in the VEGFA pathway whose expression is deregulated when si-circGPC3 is introduced into Huh7 and HepG2

No.	GeneName	Huh7- circGPC3 knockdown		HepG2- circGPC3 knockdown	
		FDR	FC	FDR	FC
1	TRIB3	7.47E-85	-2327.07	2.53E-37	2.30
2	VEGFA	2.70E-43	-1171.39	4.65E-48	1347.84
3	ABI2	7.91E-18	-465.95	3.06E-14	379.78
4	CTNNA1	3.70E-11	-280.79	6.37E-08	198.22
5	PTK2B	7.44E-10	-244.47	6.99E-09	9.97
6	CTNND1	3.53E-09	-225.64	1.33E-13	361.53
7	CYFIP1	1.32E-12	-3.94	9.82E-07	164.08
8	NRP1	3.67E-03	-2.22	6.43E-40	1114.52
9	PIK3R2	5.76E-07	-2.14	4.24E-07	5.15
10	KRAS	1.30E-45	-1235.84	4.12E-02	-1.15
11	CAV1	1.86E-14	-372.37		
12	SPHK1	3.30E-10	-254.31	7.10E-01	1.04
13	PRKACB	3.78E-08	-197.00	5.69E-01	-1.13
14	ELMO1	1.65E-08	-7.49		
15	HRAS	8.26E-12	-3.10	8.46E-01	1.06
16	NCK1	2.83E-12	-2.69	9.84E-01	-1.01
17	MAPK11	6.44E-03	-2.65		
18	AHCYL1	1.53E-10	-2.22	1.55E-07	-1.29
19	VEGFD	1.70E-03	-2.14		
20	ITPR1	7.94E-04	1.51	3.36E-61	1724.61
21	BCAR1	4.81E-01	1.03	7.45E-42	1170.12
22	PTK2	9.46E-01	-1.01	4.32E-39	1090.76
23	JUP	5.28E-01	-1.04	3.78E-35	977.84
24	PRKCD	5.35E-02	-1.14	1.98E-34	957.33
25	AXL			1.12E-32	907.27
26	PXN	8.69E-01	1.06	4.29E-12	318.15
27	NRP2	5.16E-01	1.07	1.39E-09	246.11
28	MLST8	5.29E-03	-1.23	3.10E-08	207.28
29	MAPKAPK3	1.91E-01	1.11	6.89E-07	168.48
30	VAV2	1.91E-02	-1.07	4.72E-05	6.75
31	PIK3CB	1.68E-04	1.29	2.24E-32	4.22
32	PRR5	2.78E-04	-1.26	3.92E-02	2.43
33	PRKACA	6.45E-01	-1.02	1.26E-77	2.37
34	MAPKAP1	7.03E-01	1.02	3.26E-08	2.35

FOOTNOTE: Grey cell: No expression.
